# The CspC:CspA heterodimer transduces germinant and co-germinant signals during *Clostridioides difficile* spore germination

**DOI:** 10.1371/journal.pbio.3003610

**Published:** 2026-02-02

**Authors:** Morgan E. McNellis, Gonzalo González-Del Pino, Juan A. Serrano-Jiménez, Emily R. Forster, Anca Ioana Stoica, Ekaterina E. Heldwein, Aimee Shen

**Affiliations:** 1 Department of Molecular Biology and Microbiology, Tufts University School of Medicine, Boston, Massachusetts, United States of America; 2 Graduate Program in Molecular Microbiology, Graduate School of Biomedical Sciences, Tufts University School of Medicine, Boston, Massachusetts, United States of America; Johns Hopkins University School of Medicine, UNITED STATES OF AMERICA

## Abstract

The clinically significant pathogen *Clostridioides difficile* lacks the transmembrane nutrient germinant receptors conserved in almost all spore-forming bacteria. Instead, *C. difficile* initiates spore germination using a unique mechanism that requires two signals: a bile acid germinant and a co-germinant, which can be either an amino acid or a divalent cation. While two soluble pseudoproteases, CspC and CspA, were initially identified as the germinant and co-germinant receptors, respectively, in *C. difficile*, we previously identified residues in an unstructured region of CspC that regulate the sensitivity of *C. difficile* spores to both signals. However, the mechanism by which CspC transduces these signals remained unclear. Here, we demonstrate that CspC forms a stable complex with CspA and determine the crystal structure of the CspC:CspA heterodimer. The structure reveals extensive interactions along the binding interface, including direct interactions between the unstructured region of CspC and CspA. Using structure-function analyses, we identify CspC:CspA interactions that regulate the sensitivity of *C. difficile* spores to germinant signals and show that CspA regulates the response of *C. difficile* to not only co-germinant but also germinant signals. While we show that CspA can form a homodimer and determine its crystal structure, CspA homodimerization appears unimportant for *C. difficile* spore germination. Collectively, our analyses establish the CspC:CspA heterodimer, rather than its individual constituents, as a critical signaling node for sensing both germinant and co-germinant signals. They also suggest a new mechanistic model for how *C. difficile* transduces germinant signals, which could guide the development of therapeutics against this important pathogen.

## Abstract 2

Unlike other bacteria, C. difficile must detect both a germinant signal and a co-germinant signal to trigger spore germination, but how these cues are sensed is unknown. This study finds that the CspC:CspA complex serves as a key signaling node that integrates environmental cues to regulate Clostridioides difficile spore germination.

## Introduction

*Clostridioides difficile* is a Gram-positive, spore-forming gastrointestinal pathogen that is a leading cause of hospital-acquired infections in many countries around the world [1–4]. Since *C. difficile* is an obligate anaerobe, its aerotolerant spores are its major transmissive form [5–7]. Accordingly, *C. difficile* infections begin when spores are ingested and encounter germinants in the gut that signal an environment favorable for *C. difficile* growth [5,8–10]. Germinant sensing initiates spore germination, whereby metabolically dormant spores degrade their protective cortex layer and transform into metabolically active vegetative cells [5,8]. Germination is thus essential for the ability of *C. difficile* to colonize hosts and establish infection.

*C. difficile* spore germination differs markedly from most spore-forming bacteria because this process does not require nutrient signals, such as amino acids or sugars, to initiate germination [5,8]. Instead, *C. difficile* germinates in response to gut-specific bile acids, particularly taurocholate (TA) [5,8,9]. In addition to this germinant signal, *C. difficile* spores require a second, co-germinant signal in the form of an amino acid or divalent cation, typically glycine or calcium, respectively [9,11–13]. Furthermore, *C. difficile* is only one of two spore-forming organisms that do not encode the transmembrane Ger receptors used to sense amino acid and sugar germinant signals [14]*.* Ger receptors are nutrient-gated channels [15] that bind to their nutrient germinant signals and initiate a signaling cascade that leads to spore germination [15,16]. Instead, *C. difficile* uses two soluble proteins located within the spore cortex, CspC and CspA. These two proteins are thought to sense germinant and co-germinant signals, respectively, [17,18] and activate the CspB protease, which then proteolytically activates the cortex lytic enzyme SleC [19,20]. Activated SleC degrades the protective cortex, allowing germination to proceed [19].

CspA, CspB, and CspC are members of the clostridial-specific subtilisin-like serine protease (Csp) family, which was originally identified in *Clostridium perfringens* as being responsible for the cleavage and activation of the cortex lytic enzyme SleC during germination [20,21]. Whereas the CspA, CspB, and CspC homologs encoded by *C. perfringens* and other Clostridiaceae and Lachnospiraceae family members are all predicted to be catalytically active, the only active Csp in *C. difficile* and other members of the Peptostreptococcaceae family is CspB [19,22,23]. *C. difficile* CspA and CspC harbor inactivating mutations in their catalytic triad and are thus pseudoproteases [19,22–24]. In *C. difficile*, the Csp proteins are encoded by the *cspBA-cspC* locus, with *cspBA* encoding a fusion of CspB and CspA (CspBA). During sporulation, CspBA undergoes interdomain processing by the YabG protease such that the individual CspB and CspA proteins are assembled into the mature spore [18,22]. Notably, CspC depends on CspAind to be stably incorporated into the mature spore during sporulation [22,24].

Direct binding between germinants and their receptors has not yet been demonstrated directly in any system (*C. difficile* included) due to the difficulty in recapitulating dormant spore conditions in vitro [25,26]. While prior genetic screens implicated CspC and CspA as the likely germinant and co-germinant receptors, respectively [17,18], we previously showed that mutations within an unstructured region of CspC sensitize *C. difficile* spores to both signals [27]. However, the mechanism by which CspC senses the germinant and co-germinant signals and the role of CspA in this process remained unclear. Here, we demonstrate that CspC and CspA form a stable heterodimer and determine its crystal structure. By mutating residues at the CspC:CspA interface, we identify specific regions that regulate the sensitivity of *C. difficile* spores to germinant and co-germinant signals. While we also show that CspA forms a homodimer and determine its crystal structure, mutating residues at the CspA homodimer interface minimally affects germinant and co-germinant signaling. Thus, we propose that the CspC:CspA heterodimer, rather than the individual CspC and CspA proteins, serves as a key signaling node for sensing germinant and co-germinant signals in *C. difficile*.

## Results

### CspC and CspA form a complex that does not include CspB

**Fig 1:**
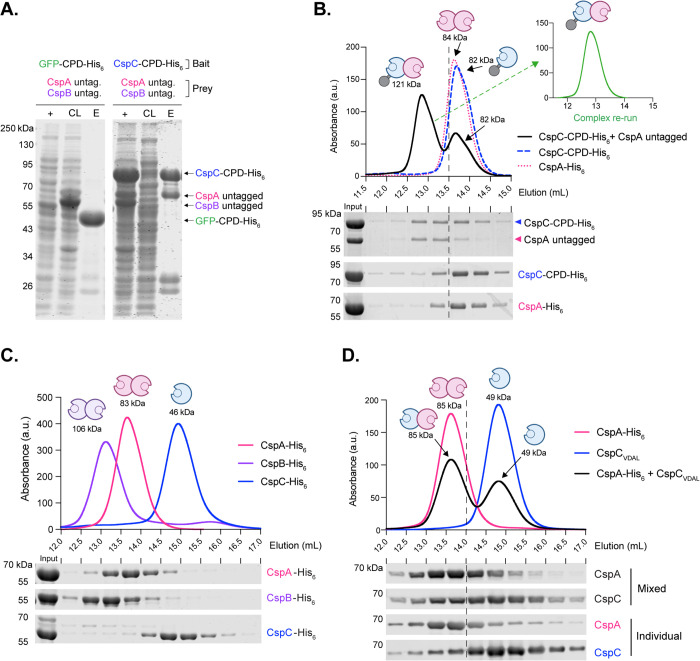
Interactions between *Clostridioides difficile* Csp proteins. **(A)** Coomassie stain of co-affinity purifications of CspC-CPD-His_6_ or GFP-CPD-His_6_ as the bait and untagged CspB and CspA as the prey. + , induced fraction; CL, cleared lysate; E, elution. **(B)** Size exclusion chromatography (SEC) analysis of the elution fraction of a CspC-CPD-His_6_:CspA co-affinity purification. The dashed line indicates the separation between the two peaks. 12.9 mL corresponds to an apparent MW of 121 kDa; 13.6 mL corresponds to an apparent MW of 84 kDa; and 13.7 mL corresponds to an apparent MW of 82 kDa. The SEC elution fractions were resolved using SDS-PAGE and stained with Coomassie. (Inset) Analysis of the stability of SEC-purified CspC-CPD-His_6_:CspA complex. The complex was purified from the 12.6–13.0 mL fraction and then re-analyzed using SEC. **(C)** SEC analyses of individual Ni^2+^-affinity-purified Csp-His_6_ proteins. 13.1 mL corresponds to an apparent MW of 106 kDa, 13.7 mL corresponds to an apparent MW of 83 kDa, and 14.9 mL corresponds to an apparent MW of 46 kDa. (bottom) Coomassie stain of SEC elution fractions resolved by SDS-PAGE. The CspB prodomain (~9 kDa) was run off the gel [19]. **(D)** Size exclusion chromatography (SEC) of separately purified CspC_VDAL_ and CspA-His_6_ variants. Separately purified proteins were mixed and incubated for 1 hr at a 1:1 stoichiometry and then resolved using SEC. (bottom) western blot analysis of the SEC fractions. All data shown are representative of three replicates. The raw gel images in this figure can be found in S1 Raw Images. The data underlying panels B, C, and D can be found in S1 Data.

Previously, we showed that CspC levels in spores are greatly reduced in the absence of CspA [22,24] and that CspC and CspA levels are also greatly reduced in the absence of CspB [24]. Therefore, we hypothesized that CspC and CspA directly interact and that this interaction may require CspB. To test these hypotheses, we co-expressed untagged CspA and CspB with His_6_-tagged CspC in *E. coli* and tested whether the untagged proteins co-purify with CspC-His_6_. To recapitulate the CspB and CspA variants generated by YabG-mediated processing of the CspBA fusion protein [18,22], we produced the individual CspB_1-582_ and CspA_583-1132_ domains [18]. Since CspC and CspA have almost identical molecular weights (MWs) of 60 kDa, we distinguished the two proteins from each other by tagging CspC with a *Vibrio cholerae* MARTX toxin cysteine protease domain (CPD) [28] fused to a C-terminal hexahistidine tag (CPD-His_6_, 24 kDa) [29,30]. CspB is readily distinguished from CspC and CspA because CspB undergoes autoprocessing of its N-terminal prodomain, and the processed CspB has a MW of ~55 kDa [19]. The pull-down analyses revealed that untagged CspA, but not untagged CspB, efficiently co-purifies with CspC-CPD-His_6_, suggesting that CspC and CspA form a complex (Fig 1A). CspA failed to co-purify with a GFP fusion protein (GFP-CPD-His_6_), used as a negative control, indicating that the CspC:CspA interaction is specific (Fig 1A). Interestingly, we found that CspC-CPD-His_6_ pulled down untagged CspBA fusion protein at considerably lower levels than untagged CspA (S1 Fig), suggesting that YabG-mediated proteolytic processing of CspBA promotes CspC binding to CspA.

To assess the stability of the CspC-CPD-His_6_:CspA complex, we used size exclusion chromatography (SEC) (Fig 1B). Two distinct peaks were observed by SEC. Peak 1 eluted with an apparent MW of 121 kDa and contained CspA and CspC-CPD-His_6_ at an apparent ratio of 1:1 (Fig 1B, SDS-PAGE), consistent with a CspC-CPD-His_6_:CspA heterodimer. Peak 2 eluted with an apparent MW of 84 kDa. This peak contained mostly monomeric CspC-CPD-His_6_, consistent with its predicted MW of 86 kDa (Fig 1B). Despite CspA having a predicted MW of 60 kDa, a small amount of untagged CspA was detected in this smaller peak, suggesting that CspA may form a dimer when not in complex with CspC. Still, nearly all the untagged CspA was found in the CspC-CPD-His_6_:CspA peak, and when the complex was re-run on SEC, it eluted as a single peak at the same volume as the original CspC-CPD-His_6_:CspA complex, indicating that the complex was stable (Fig 1B, inset). SEC analyses of individually purified CspC-His_6_ and CspA-His_6_ confirmed that CspA-His_6_ formed an apparent homodimer, whereas CspC-His_6_ was monomeric, consistent with our previous study [27] (Fig 1C).

To rule out any potential contributions of the CPD tag to this interaction, we individually purified CspC and CspA, mixed the two proteins at a 1:1 ratio, and analyzed the CspC:CspA heterodimer formation by SEC. Untagged CspC was first purified by inducing the CspC-CPD-His_6_ fusion protein to undergo autoprocessing using inositol hexakisphosphate (InsP_6_) [29,30], which adds a Val-Asp-Ala-Leu (VDAL) sequence to the C terminus of CspC. When CspC_VDAL_ and CspA-His_6_ were mixed, a larger portion of the mixture eluted in peak 1, where the CspA homodimer typically elutes, consistent with CspC:CspA heterodimer formation (Fig 1D). These data indicate that CspA has a higher affinity for CspC than for itself and that the CspC:CspA heterodimer can form even when CspC and CspA are produced individually.

### CspB and CspA form homodimers, but CspC does not

Since CspA forms an apparent homodimer detected by SEC, we assessed the ability of *C. difficile* Csp proteins to interact in pairwise analyses. Untagged CspA, CspB, or CspC were co-produced with CPD-His_6_-tagged CspA, CspB, and CspC variants, respectively, in *E. coli*, and the resulting pairwise interactions were analyzed. As expected, untagged CspC co-purified with CspA-CPD-His_6_ (S2 Fig), indicating that this interaction occurs when the tags are reversed. Untagged CspA co-purified with CspA-CPD-His_6_ (S2 Fig), consistent with the ability of CspA to homodimerize. Untagged CspB also co-purified with CspB-CPD-His_6_ (S2 Fig), suggesting that CspB forms a homodimer. Consistent with these data, SEC analyses of the individually purified Csp proteins (Fig 1C) confirmed that CspB and CspA each form homodimers. In contrast, untagged CspC was not pulled down by either CspB-CPD-His_6_ or CspC-CPD-His_6_ (S2 Fig), and it eluted as a monomer when individually purified and analyzed via SEC (Fig 1C). None of the untagged Csps interacted with the GFP-CPD-His_6_ control (S2 Fig). These results are consistent with the known crystal structures of the *C. perfringens* CspB dimer^16^ and *C. difficile* CspC monomer^20^ and provide the first evidence that *C. difficile* CspA forms a homodimer.

### CspA forms an antiparallel homodimer

**Fig 2:**
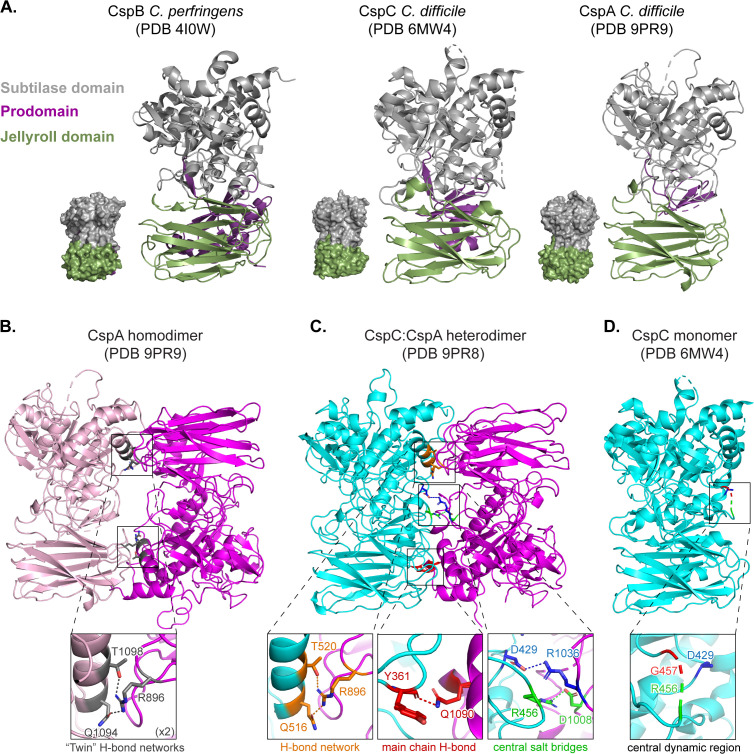
Crystal structures of the *Clostridioides difficile* CspA homodimer and CspC:CspA heterodimer. Ribbon and space-fill models of **(A)** *C. perfringens* CspB (PDB RI0W [19]), *C. difficile* CspC (PDB 6MW4 [27]), and *C. difficile* CspA (chain B) (this work—PDB 9PR9). The subtilase domains are shown in gray, jellyroll domains in green, and prodomains in purple. Ribbon models of the: **(B)** Crystal structure of the CspA homodimer solved to 3.2 Å (PDB 9PR9). Inset shows a close-up of one of two tripartite hydrogen bond networks found at the interface of the CspA homodimer. **(C)** Crystal structure of the CspC:CspA_F944E/Y1092E_ (CspC:CspA_EE_) heterodimer solved to 3.4 Å (PDB 9PR8). CspC is shown in cyan, and CspA is shown in magenta. Inset (left): tripartite hydrogen bond network between residues Q516_CspC_, T520_CspC_, and R896_CspA_ with distances of 2.7 Å and 3.1 Å, respectively. Inset (center): hydrogen bond between the Y361_CspC_ main chain and the Q1090_CspA_ residue with a distance of 2.8 Å. Inset (right): two salt bridges between CspC and CspA. The D429_CspC_, R1036_CspA_ salt bridge interaction is a distance of 3.6 Å, while the R456_CspC_, D1008_CspA_ is 2.9 Å. **(D)** Crystal structure of CspC monomer (PDB 6MW4 [27]). Inset shows unstructured residues in the CspC monomer that form salt-bridges in the CspC:CspA heterodimer.

To gain insight into the functional importance of the CspA homodimer uncovered by our co-affinity purification analyses, we determined the crystal structure of CspA. The structure was phased by molecular replacement using the known structure of the *C. difficile* CspC [27] and refined to 3.2 Å resolution (S1 Table). The *C. difficile* CspA structure is very similar to those of *C. difficile* CspC and *C. perfringens* CspB [19,27] (Fig 2A) and can be superimposed with RMSDs of 1.22 and 1.44 Å, respectively. All three proteins are composed of an N-terminal prodomain followed by the subtilase domain that contains the central jellyroll domain insertion unique to the Csp subtilisin-like serine protease family [19,27] (Figs 2A and S3).

The asymmetric unit of the CspA crystals consists of two antiparallel, asymmetric homodimers, CspA_AB_ and CspA_CD_ (S4 Fig). The four copies of CspA are very similar and can be superimposed with RMSDs ranging between 0.30 and 0.42 Å. Chains A and C are similar to each other and the most complete, with 534 residues resolved out of 559 total residues. Chains B and D are also similar to each other but less complete, with 489 and 487 residues, respectively, resolved out of 559 total residues. The two CspA homodimers can be superimposed with an RMSD of 0.32 Å. The N-terminal prodomains in chains A and C are better resolved than their counterparts in chains B and D in two regions (S4 Fig). The A and C protomers are better resolved likely because of crystal contacts with neighboring protomers (S5 Fig).

To map residues at the CspA homodimer interface, we analyzed the buried surface area and interactions (hydrogen bonds and salt bridges) using PiSA interface analysis [31] (S2 Table). In the CspA homodimer, the interface features two symmetrical tripartite hydrogen-bond networks formed between the jellyroll and subtilase domains, termed twin H-bond networks (Fig 2B). Each H-bond network is formed by three residues, Arg896, Gln1094, and Thr1098 (for consistency, the numbering for CspA is based on the CspBA fusion protein) (Fig 2B, inset). Notably, there were no interactions at the center of the homodimer (Fig 2B), so the twin H-bond networks make up the majority of the CspA homodimer interface.

### The CspC:CspA heterodimer has an extensive interface

To investigate the functional importance of the CspC:CspA heterodimer, we determined its crystal structure. Initial attempts to crystallize CspC_VDAL_:CspA failed to yield diffraction-quality crystals, so we hypothesized that the CspA homodimer in the CspC_VDAL_:CspA heterodimer preparations was interfering with the crystallization, based on a prior report that showed that homodimerization of Munc13-1 hindered crystallization of the Munc13-1:RIM2α heterodimer [32]. To mitigate this issue, we used a CspA_F944E/Y1092E_ (CspA_EE_) mutant that increases CspC_VDAL_:CspA_EE_ heterodimer formation (S6 Fig). By working with a CspA mutant favoring the formation of the CspC:CspA heterodimer, we were able to crystallize it. The crystal structure of the CspC_VDAL_:CspA_EE_ heterodimer was determined to 3.35 Å resolution (Fig 2C and S1 Table).

There are many similarities between the CspC:CspA heterodimer and the CspA homodimer. Like the CspA homodimer (Figs 2B and S4), the CspC:CspA heterodimer has an antiparallel orientation (Fig 2C and S7 Fig). Likewise, there are two crystallographically independent CspC:CspA heterodimers in the asymmetric unit, with small variations between their structures (RMSD of 0.40 Å between the two CspC:CspA heterodimers, 0.41 Å between CspC_B_ and CspC_D_, and 0.45 Å between CspA_A_ and CspA_C_) (S7 Fig). Remarkably, the H-bond network formed by Arg896, Gln1094, and Thr1098 at the interface of the CspA homodimer is conserved at the CspC:CspA heterodimeric interface. Specifically, the same residue, Arg896_CspA_, forms hydrogen bonds with Gln516_CspC_ and Thr520_CspC_, which are homologous to Gln1094_CspA_ and Thr1098_CspA_ (Fig 2B, inset and 2C, left inset).

However, there are also notable differences. First, the CspC:CspA heterodimer has a more extensive interface than the CspA homodimer (Fig 2B and 2C). Indeed, the heterodimeric interface of the CspC:CspA buries a significantly larger area, 2,024 Å^2^ on average, compared to the homodimeric interface of CspA, which buries 1,610 Å^2^ on average (S2 Table). Second, in the CspC:CspA heterodimer, there is only one H-bond network at the interface (Fig 2C). Instead of the second H-bond network, a series of interactions tether the CspC jellyroll domain to the CspA subtilase domain, including a hydrogen bond between the carbonyl group of CspC residue Tyr361 and the amide of CspA residue Gln1090 (Fig 2C, center inset). Lastly, there are two salt bridges at the center of the interfaces of both heterodimers, between Asp429_CspC_ and Arg1036_CspA_ and between Arg456_CspC_ and Asp1008_CspA_ (Fig 2C, right inset). Interestingly, the CspC residues forming these salt bridges are unstructured in the CspC monomer structure [27] (Fig 2C and 2D, insets). Notably, the D chain Arg456_CspC_ is unstructured in the CspC_D_:CspA_C_ heterodimer, so one of these salt bridges (Arg456_CspC_ and Asp1008_CspA_) is absent (S7C Fig, inset). Previously, we showed that mutations of residues Asp429_CspC_, Arg456_CspC_, and Gly457_CspC_ enhance the sensitivity of spores to germinant and/or co-germinant signals [27]. These mutations would be expected to disrupt the salt bridges at the binding interface, potentially destabilizing the CspC:CspA heterodimer. Thus, the CspC:CspA heterodimer may be important for sensing both germinant and co-germinant signals.

### Mutation of the two salt bridges at the CspC:CspA heterodimer interface increases the sensitivity of *C. difficile* spores to TA germinant but has no effect on heterodimer stability

**Fig 3:**
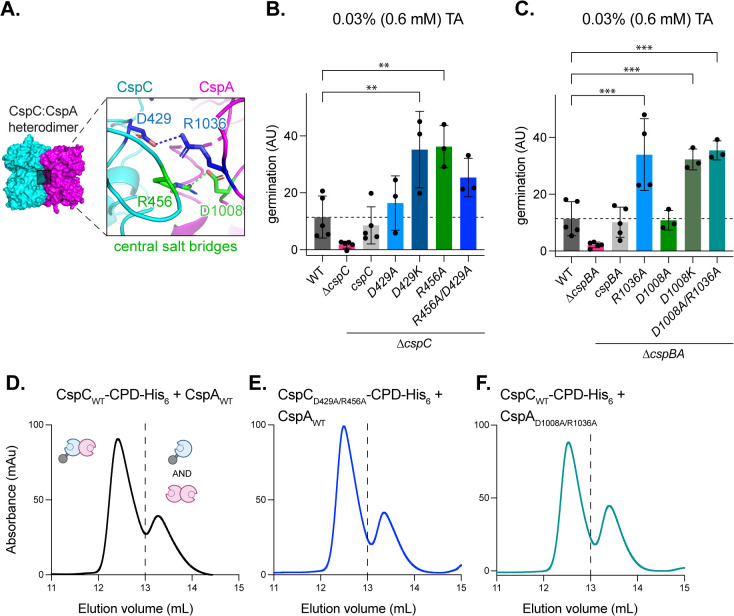
Mutation of two salt bridges at the CspC:CspA heterodimer interface increases the sensitivity of *Clostridioides difficile* spores to TA germinant. **(A)** Two salt bridges at the center of the CspC:CspA heterodimer interface. **(B, C)** Gemination levels based on the change in optical density (OD_600_) of purified spores suspended in rich medium over time following the addition of 0.6 mM taurocholate (TA). Germination in arbitrary units (AU) was calculated using the area below inverted OD_600_ curves (S8 Fig). *cspC* complementation mutants were constructed in a *∆cspC* background **(B)**. *cspBA* complementation mutants were constructed in a *∆cspBA* background **(C)**. CspA residue numbers are based on the full-length CspBA fusion protein. Statistical significance relative to WT was determined using a one-way ANOVA and Dunnett’s multiple comparisons test. *** *p* < 0.001, ** *p* < 0.01. **(D–F)** Size exclusion chromatography analysis of CspC-CPD-His_6_ and CspA co-affinity purifications with the indicated mutants. The dashed line indicates the separation between the two peaks. All *C. difficile* data shown **(B, C)** are representative of a minimum of three independent replicates. The SEC data shown were obtained once. The data underlying panels B–F can be found in S1 Data.

To dissect the functional roles of the aforementioned salt bridges, we mutated Asp429_CspC_, Arg456_CspC,_ Arg1036_CspA,_ and Asp1008_CspA_ (Figs 2C and 3A) and tested the effect of the point mutations on *C. difficile* germination using an optical density (OD_600_)-based germination assay, which measures the characteristic OD_600_ drop of germinating spores that occurs due to spore cortex degradation and core rehydration [17,18,27] (S8 Fig). The point mutations were introduced into *cspC* or *cspBA* complementation constructs expressed from an ectopic locus [33], and the resulting mutants were tested for their ability to rescue the germination of *∆cspC* and ∆*cspBA* mutants.

We found that *cspC*_D429A_ spores were only slightly more sensitive to TA germinant, whereas *cspC*_R456A_ spores were significantly more sensitive to TA germinant (Fig 3B, *p* < 0.01). These results are consistent with our prior report showing that substitution at residue R456_CspC_ (*cspC*_R456G_) resulted in spores that were more sensitive to TA germinant than a substitution at residue D429_CspC_ (*cspC*_D429K_) [27]. The double mutant *cspC*_D429A/R456A_ also produced spores with increased TA germinant sensitivity, albeit slightly less than spores carrying the single *cspC*_R456A_ mutation (Fig 3B). *cspA*_R1036A_ and *cspA*_D1008K_ spores were significantly more sensitive to TA germinant (Fig 3C, *p* < 0.001), whereas *cspA*_D1008A_ spores exhibited WT TA germinant sensitivity (Fig 3C). *cspA*_D1008A/R1036A_ double mutant spores germinated similarly to the single *cspA*_R1036A_ mutant spores (Fig 3C).

Since we previously showed that mutations in this region affected co-germinant sensitivity [27], we tested the response of each of these mutants to the co-germinant calcium. To do this, we performed the OD-based germination assay in a buffer rather than rich medium, keeping the TA concentration constant as we varied the concentration of CaCl_2_ [27]. We did not use glycine for this assay since endogenous calcium released from the spore during germination acts as a co-germinant; therefore, glycine-enhanced germination cannot be uncoupled from the contribution of calcium [12]. These analyses revealed that substitutions in the central dynamic region of the heterodimer also increased the sensitivity of spores to calcium, with the mutant spores exhibiting similar phenotypic trends to those observed in the TA-based assay (Figs 3B, 3C, and S9).

Western blot analyses confirmed that none of the salt-bridge substitutions altered Csp levels in spores relative to WT, although slight differences in mobility were observed with the CspC and CspA substitutions (S10 Fig). These differences in mobility are likely due to the altered charge of the Csp variants. Of note, increasing the resolution of the SDS-PAGE revealed multiple isoforms of CspA (S10 Fig). The three distinct bands observed may reflect the ability of YabG to target multiple sites during its interdomain processing of CspBA [18,22].

The location of these residues at the interface of the CspC:CspA heterodimer suggested that their substitution may destabilize the complex. To test the effect of these mutations on heterodimer stability in vitro, we used co-affinity purification followed by SEC analysis. We found that substituting both the CspC and CspA residues involved in the two salt bridges for alanine (CspC_D429A/R456A_ or CspA_D1008A/R1036A_) had no effect on heterodimer stability in vitro, with both mutant heterodimers eluting like WT (Fig 3D–3F). Taken together, these data show that mutations in the central salt bridge of the CspC:CspA heterodimer increase TA germinant sensitivity but have no effect on heterodimer stability in vitro.

### Mutations in the CspC:CspA heterodimer peripheral H-bonds reduce the sensitivity of *C. difficile* spores to TA germinant and have a minimal effect on heterodimer stability

**Fig 4:**
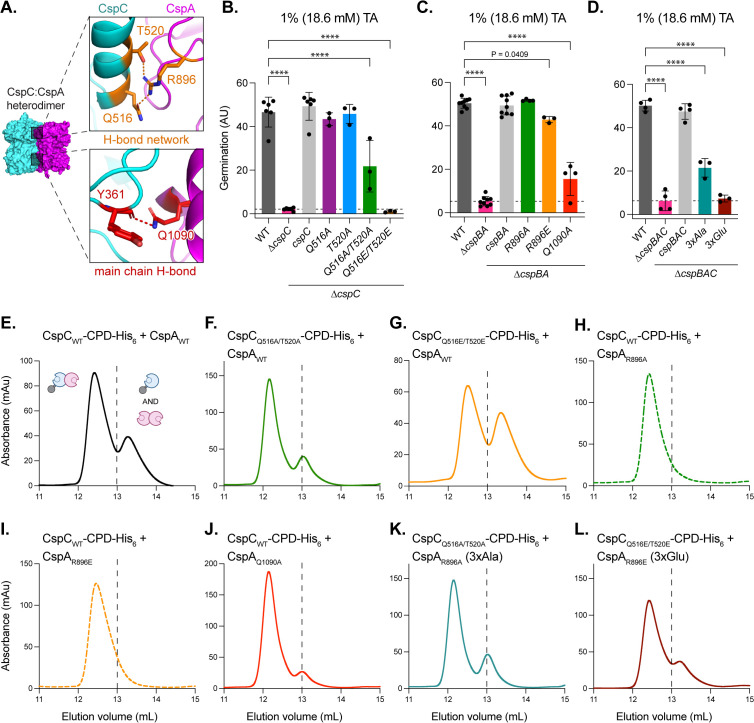
Mutations in the CspC:CspA heterodimer peripheral H-bonds impair germination and impact heterodimer formation. **(A—top)** Tripartite hydrogen bond network in the CspC:CspA heterodimer, and **(A—bottom)** hydrogen bond between the Y361_CspC_ main chain and the Q1090_CspA_ residue. **(B, C)** Germination levels based on the change in optical density (OD_600_) of purified spores suspended in rich medium over time following the addition of the 18.6 mM taurocholate (TA). Germination in arbitrary units (AU) was calculated using the area below inverted OD_600_ curves (S8 Fig). *cspC* complementation mutants were constructed in a *∆cspC* background **(B)**. *cspBA* complementation mutants were constructed in a *∆cspBA* background **(C)**. *cspBAC* complementation mutants were constructed in a *∆cspBAC* background **(D)**. CspA residue numbers are based on the full-length CspBA fusion protein. *∆cspBAC/3xAla* = triple-Ala substitution *cspBA*_*R896A*_*-cspC*_*Q516A/T520A*_, ∆*cspBAC/3xGlu* = triple-Glu substitution *cspBA*_*R896E*_*-cspC*_*Q516E/T520E*_. Statistical significance relative to WT was determined using a one-way ANOVA and Dunnett’s multiple comparisons test. **** *p* < 0.0001. **(E–L)** Size exclusion chromatography (SEC) analyses of CspC-CPD-His_6_:CspA co-affinity purifications. The dashed line indicates the separation between the two peaks. The trace for CspC_WT_-CPD-His_6_:CspA_WT_ is reproduced from Fig 3D. All *Clostridioides difficile* data shown **(B–D)** are representative of a minimum of three independent replicates. E–L are representative of a minimum of two independent replicates, except for CspC_WT_-CPD-His_6_:CspA_Q1090A_, which was resolved via SEC once. The data underlying panels B–L can be found in S1 Data.

To further probe the role of the CspC:CspA heterodimeric interface in *C. difficile* germination, we next targeted the H-bond network formed by the residues Gln516_CspC_, Thr520_CspC_, and Arg896_CspA_, using mutagenesis (Figs 2C and 4A). Previously, we showed that *cspC*_Q516E_ and *cspC*_Q516R_ spores had WT sensitivity to TA germinant [27]. Here, we found that single alanine substitutions *cspC*_Q516A_, *cspC*_T520A_, and *cspA*_R896A_ also had no effect on sensitivity to TA germinant at high concentrations (18.6 mM) (Fig 4B and 4C). However, double *cspC*_Q516A/T520A_ and triple *cspBA*_R896A_:*cspC*_Q516A/T520A_ mutant spores had decreased germinant sensitivity (Fig 4B and 4D). Moreover, substituting two or three H-bond network residues with glutamates (CspC_Q516E/T520E_ and CspBA_R896E_:CspC_Q516E/T520E_), which should disrupt the H-bond network by charge repulsion, completely abrogated germinant sensing (Fig 4B and 4D); even the single glutamate substitution CspBA_R896E_ partially decreased germinant sensitivity (Fig 4C).

We next probed the role of a single hydrogen bond between the main chain carbonyl group of residue Tyr361_CspC_ and residue Gln1090_CspA_ on the reciprocal side of the heterodimer interface from the H-bond network (Figs 2C and 4A) by substituting residue Gln1090_CspA_ for alanine. Similar to the heterodimer H-bond network mutations, the *cspA*_Q1090A_ mutation greatly reduced TA germinant sensing even at high concentrations (18.6 mM) (Fig 4C, *p* < 0.0001). None of the mutations affected Csp levels in spores (S11 Fig), indicating that the observed germination defects are not due to reduced levels of germination proteins in spores. Taken together, these data imply that disrupting the H-bonds at the periphery of the CspC:CspA heterodimer interface impairs TA germinant sensing.

To test the effects of peripheral H-bond substitutions on heterodimer stability in vitro, we used co-affinity purification followed by SEC (Fig 4E–4L). Most mutations had no effect on the stability of the CspC:CspA heterodimer. However, the double glutamate mutant CspC_Q516E/T520E_ had a reduced CspC:CspA heterodimer peak by SEC (Fig 4G), and the amount of untagged CspA in the co-affinity pull-down was reduced compared to WT CspC (S12A Fig). Notably, similar to the WT heterodimer (Fig 1B), the CspC_Q516E/T520E_-CPD-His_6_:CspA heterodimer remained stable upon being re-purified using SEC (S13A Fig). These data demonstrate that the CspC_Q516E/T520E_-CPD-His_6_:CspA heterodimer is stable, indicating that the SEC analyses primarily reflect the efficiency of the co-affinity pull-down. Consistent with this conclusion, variable amounts of CspC_Q516E/T520E_-CPD-His_6_:CspA heterodimer in the co-affinity purifications resulted in different proportions of CspC_Q516E/T520E_-CPD-His_6_:CspA heterodimer to free CspC-CPD-His_6_ and CspA homodimer observed in the SEC analyses (S14 Fig). This variability may result from the sensitivity of competitive interactions, caused by the change in equilibria between the CspC:CspA heterodimer and CspA:CspA homodimer mutants, which can occur due to slight variations in the *E. coli* growth conditions or incubation times during co-affinity purification. Nonetheless, our data support the conclusion that the Q516/T520_CspC_ residues promote CspC:CspA heterodimerization. Moreover, since the *cspC*_Q516E/T520E_ mutant is one of two germination-null mutants (Fig 4B), disruption of this H-bond network reduces both heterodimer formation in vitro and TA germinant sensing.

All other mutants tested eluted similarly to the WT heterodimer, apart from the CspA_R896A_ and CspA_R896E_ variants, which eluted exclusively in complex with CspC (Fig 4H and 4I). Notably, Arg896_CspA_ also participates in H-bond networks at the CspA homodimer interface (Fig 2B, inset). Since the second peak in the WT CspC-CPD-His_6_:CspA SEC trace contains both the CspC monomer and some CspA homodimer (Figs 1B and 4E), disrupting the interactions formed by Arg896_CspA_ likely impairs CspA homodimerization, promoting CspC:CspA interaction.

Like the CspC_Q516E/T520E_ mutant, the triple glutamate substitution in the H-bond network of the CspC:CspA heterodimer (CspC_Q516E/T520E_:CspA_R896E_) abrogated germination at high concentrations of TA germinant (Fig 4D). However, unlike the double glutamate mutant CspC_Q516E/T520E_ (Fig 4G), the triple mutant CspC_Q516E/T520E_:CspA_R896E_ eluted like the WT heterodimer (Fig 4L). Since CspA_R896E_ may impair CspA homodimerization, promoting CspC:CspA interaction, it is possible that combining this mutant with CspC_Q516E/T520E_ counterbalances the destabilizing effect of the CspC mutations, resulting in an SEC trace similar to the WT heterodimer. Regardless, these data reveal that there is no direct correlation between heterodimer stability in vitro and TA germinant sensitivity in spores and that the H-bond network at the interface of CspC:CspA is important for heterodimerization and critical for germinant sensing.

### Mutations in the CspA H-bond networks disrupt CspA homodimerization but have variable effects on germination

**Fig 5:**
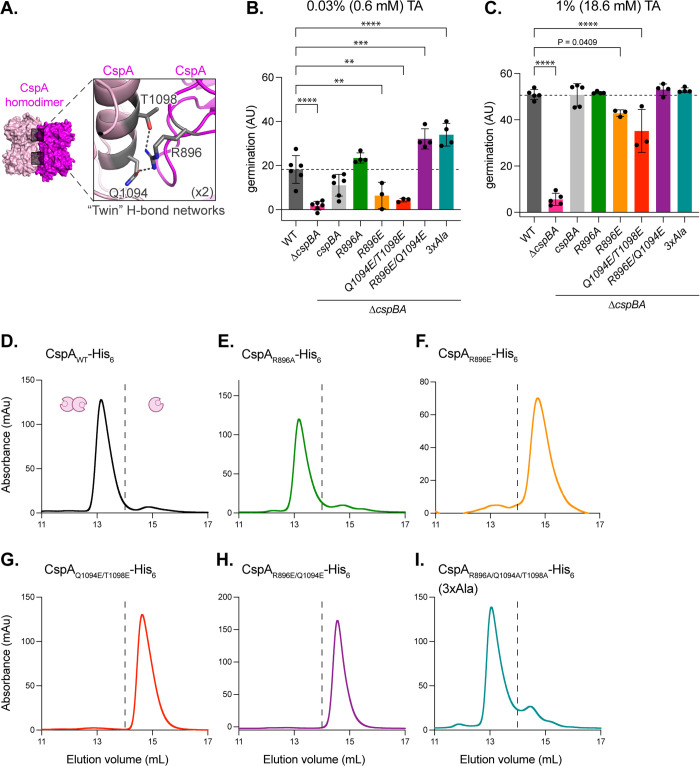
Mutations in the CspA twin H-bond networks disrupt CspA homodimerization but have variable effects on germination. **(A)** One of the two tripartite hydrogen bond networks at the CspA homodimer interface. **(B, C)** Germination levels based on the change in optical density (OD_600_) of purified spores suspended in rich medium over time following the addition of the indicated concentration of taurocholate (TA). Germination in arbitrary units (AU) was calculated using the area below inverted OD_600_ curves (S8 Fig). *cspBA* complementation mutants were constructed in a *∆cspBA* background. CspA residue numbers are based on the full-length CspBA fusion protein. ∆*cspBA/3xAla* = *cspBA*_*R896A/Q1094A/T1098A*_. The *cspBA*_*R896A*_ and *cspBA*_*R896E*_ measurements presented in **(C)** are reproduced from Fig 4C. Statistical significance relative to WT was determined using a one-way ANOVA and Dunnett’s multiple comparisons test. **** *p* < 0.0001, *** *p* < 0.001, ** *p* < 0.01. **(D–I)** Size exclusion chromatography (SEC) analyses of CspA-His_6_ variants following affinity purification. The dashed line indicates the separation between the two peaks. All *Clostridioides difficile* data shown **(B, C)** are representative of a minimum of three independent replicates. The data shown in D–I were obtained once, except for CspA_R896A_-His_6_, which was resolved via SEC using three independent replicates. The data underlying panels B–I can be found in S1 Data.

While our analyses pointed to the importance of the CspC:CspA heterodimer in *C. difficile* germinant signaling, the role of the CspA homodimer detected in vitro (Figs 1 and 2) in germination remained unclear. To address this, we designed mutations targeting the twin H-bond networks in the CspA homodimer, formed by Arg896, Gln1094, and Thr1098 (Figs 2B and 5A). Arg896_CspA_ is also involved in the analogous H-bond network in the CspC:CspA heterodimer (Fig 2B, inset and 2C, left inset), and Arg896_CspA_ mutants reduced *C. difficile* TA germinant sensing and favored CspC:CspA heterodimer formation in vitro (Fig 4). To further interrogate the role of the twin H-bond networks in the CspA homodimer, we generated *cspA* mutants encoding double and triple substitutions targeting H-bond network residues. Similar to *cspBA*_R896E_ mutant spores, *cspBA*_Q1094E/T1098E_ mutant spores were less sensitive to TA germinant at both low and high germinant concentrations (Fig 5B and 5C). While the *cspBA*_Q1094E/T1098E_ mutant spores were less sensitive to TA germinant at high concentrations of germinant (18.6 mM, Fig 5C), these phenotypes were relatively subtle compared to the analogous mutations in the CspC:CspA heterodimer H-bond network, which completely abrogated TA germinant sensing (*cspC*_Q516E/T520E_, Fig 4B). By contrast, *cspBA*_R896E/Q1094E_ and *cspBA*_R896A/Q1094A/T1098A_ (*3xAla*) mutant spores were more sensitive to low concentrations of TA germinant (0.6 mM) (Fig 5B). Since none of the CspA mutations impacted Csp levels in spores in western blot analyses (S15 Fig), these data indicate that mutation of the CspA homodimer H-bond networks had relatively minor and variable effects on germinant sensitivity compared to the analogous mutations of the H-bond network in the CspC:CspA heterodimer (Fig 4).

To determine how the H-bond network substitutions affected CspA homodimerization in vitro, we analyzed the SEC profiles of purified CspA-His_6_ variants. While alanine substitutions in H-bond network residues (CspA_R896A_-His_6_ and CspA_R896A/Q1094A/T1098A_-His_6_) did not affect CspA homodimerization (Fig 5D, 5E, and 5I), even single glutamate substitutions were sufficient to disrupt the CspA homodimer formation (CspA_R896E_-His_6_, CspA_Q1094E/T1098E_-His_6_, and CspA_R896E/Q1094E_-His_6_, Fig 5F–5H). Importantly, we found no correlation between the effect of the mutations on CspA homodimer stability and *C. difficile* spore sensitivity to TA germinant. The double glutamate substitution *cspBA*_R896E/Q1094E_ disrupted CspA homodimerization in vitro (Fig 5H) and slightly increased spore sensitivity to TA germinant (Fig 5B), whereas other glutamate substitutions (CspA_R896E_ and CspA_Q1094E/T1098E_) disrupted CspA homodimer formation (Fig 5F and 5G) and decreased spore sensitivity to TA germinant (Fig 5B and 5C). Further, CspA substitutions in the other residues of the homodimer H-bond network stabilized the CspC:CspA heterodimer in vitro in SEC analyses (S16 Fig), further supporting the hypothesis that disruption of the CspA homodimer promotes the CspC:CspA heterodimer formation. Since no notable correlation was observed between CspA homodimer formation in vitro and *C. difficile* spore TA germinant sensitivity, our data suggest that CspA homodimerization, which occurs under in vitro conditions, has unclear physiological significance.

### The CspC:CspA heterodimer is extremely stable in the presence of germinants and co-germinants

**Fig 6:**
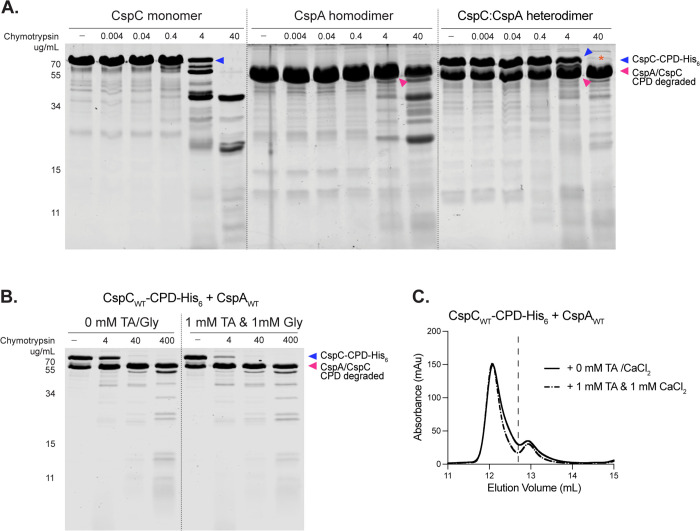
The CspC:CspA heterodimer is extremely stable in the presence and absence of germinants and co-germinants. **(A, B)** Limited proteolysis analyses of purified CspC, CspA, and the CspC-CPD-His_6_:CspA heterodimer using the indicated concentrations of chymotrypsin. CspA-His_6_ and CspC-CPD-His_6_:CspA heterodimer were affinity-purified. The CspC monomer was affinity-purified as a CspC-CPD-His_6_ fusion. Cleavage of the CspC-CPD-His_6_:CspA heterodimer at 40 μg/mL chymotrypsin (*) is due to digestion of the CPD-His_6_ tag (S17 Fig). **(B)** Limited proteolysis of purified CspC-CPD-His_6_:CspA heterodimer in the presence and absence of the indicated concentrations of taurocholate (TA) and glycine (Gly). Ca^2+^ co-germinant was not tested in this assay because high concentrations of CaCl_2_ inhibit chymotrypsin activity. **(C)** Size exclusion chromatography analyses of CspC-CPD-His_6_:CspA co-affinity purifications in the presence or absence of the indicated concentrations of TA and CaCl_2_. All data shown are representative of at least two independent replicates. The raw gel images in panels A and B can be found in S1 Raw Images. The data underlying panel C can be found in S1 Data.

Since our mutagenesis revealed that residues at the CspC:CspA heterodimer interface are important for germinant sensing (Figs 3 and 4), we considered the possibility that disruption of the CspC:CspA heterodimer is a key step in TA germinant and co-germinant sensing. However, despite extensive mutagenesis across the CspC:CspA heterodimer interface, we identified only a single CspC variant that partially disrupted the heterodimer in vitro (CspC_Q516E/T520E_, Fig 4G). This finding strongly suggests that the CspC:CspA heterodimer is extremely stable. To assess its stability, we performed limited proteolysis on the CspC-CPD-His_6_:CspA heterodimer, the CspC-CPD-His_6_ monomer, and the CspA-His_6_ homodimer. Consistent with prior work [27], the CspC monomer was sensitive to high levels of chymotrypsin (Fig 6A). The CspA homodimer was similarly sensitive to chymotrypsin (Fig 6A). By contrast, the CspC:CspA heterodimer was highly resistant to proteolysis by chymotrypsin, with the most notable degradation occurring with the top ~82 kDa CspC-CPD-His_6_ band at 40 µg/mL of chymotrypsin (Fig 6A). By analyzing the limited proteolysis reactions with CspC and CPD antibodies using western blotting, we determined that the CPD tag was susceptible to proteolysis while CspC remained largely intact (S17 Fig). Notably, substitutions in the central dynamic region (CspC-CPD-His_6_:CspA_D1008A-R1036A_) and in the H-bond network (CspC_Q516E-T520E_-CPD-His_6_:CspA) of the heterodimer did not change the susceptibility of the complex to proteolysis (S18A and S18B Fig). This is consistent with our findings that both the WT and CspC_Q516E-T520E_-CPD-His_6_:CspA heterodimer complexes remain stable upon being re-run on the SEC column (Figs 1B and S13A). Taken together, these data show that the heterodimer is a stable complex resistant to proteolysis even with the amino-acid substitutions at its interface.

We next considered the possibility that germinants and co-germinants might disrupt the heterodimer or induce conformational changes detectable by limited proteolysis. However, dual exposure to TA germinant and the co-germinant glycine did not alter the protease sensitivity of the WT or mutant CspC:CspA heterodimers (Figs 6B, S18A, and S18B). We did not test the influence of calcium on the digestion of the heterodimer, as calcium can alter the activity of chymotrypsin [34]. We next assessed whether the CspC-CPD-His_6_:CspA heterodimer might be destabilized during SEC in the presence of TA germinant and calcium co-germinant, since this method does not analyze proteins at equilibrium. No difference in the SEC profiles of the WT or mutant heterodimers was observed in the presence or absence of germinant and co-germinant (Figs 6C, S19A, and S19B). Thus, the presence of the TA germinant and the co-germinants does not affect the stability or overall conformation of the CspC:CspA heterodimer in vitro.

### Two regions of the CspC:CspA heterodimer function independently to regulate germinant and co-germinant signal integration

**Fig 7:**
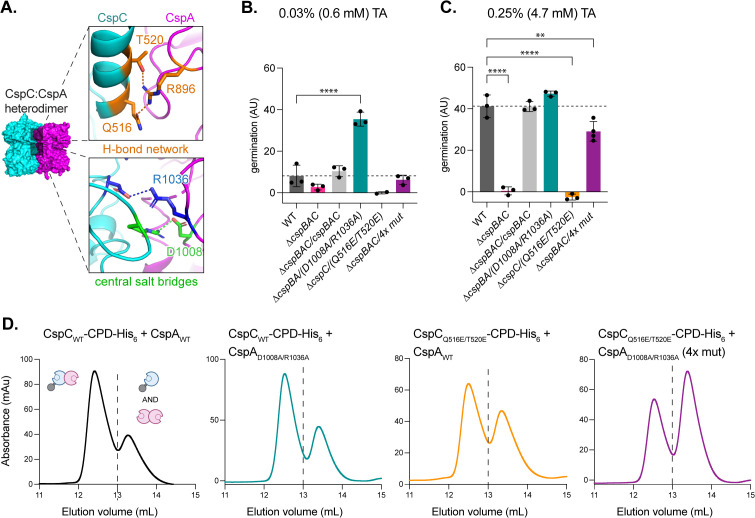
Two regions of the CspC:CspA heterodimer function independently to regulate germinant and co-germinant signal integration. **(A—top)** Tripartite hydrogen bond network in the CspC:CspA heterodimer, and **(A—bottom)** salt bridges at the center of the CspC:CspA heterodimer interface. **(B, C)** Gemination levels based on the change in optical density (OD_600_) of purified spores suspended in rich medium over time following the addition of 0.6 mM taurocholate (TA) **(B)** or 4.7 mM TA **(C)**. Germination in arbitrary units (AU) was calculated using the area below inverted OD_600_ curves (S8 Fig). Germination phenotypes of double and quadruple mutants were best resolved at 4.7 mM TA (S8 Fig). *cspBAC* complementation mutants were constructed in a *∆cspBAC* background; *cspBA* complementation mutant was constructed in a *∆cspBA* background; and *cspC* complementation mutants were constructed in a *∆cspC* background. CspA residue numbers are based on the full-length CspBA fusion protein. ∆*cspBAC/4x mut* = *cspBA*_*D1008A/R1036A*_*-cspC*_*Q516E/T520E*_. Statistical significance relative to WT was determined using a one-way ANOVA and Dunnett’s multiple comparisons test. **** *p* < 0.0001, ** *p* < 0.01. **(D)** Size exclusion chromatography (SEC) analyses of CspC-CPD-His_6_:CspA co-affinity purifications. The dashed line indicates the separation between the two peaks. The traces for CspC_WT_-CPD-His_6_:CspA_WT_, CspC_WT_-CPD-His_6_:CspA_D1008A/R1036A_, and CspC_Q516E/T520E_-CPD-His_6_:CspA_WT_ are reproduced from Figs 3D, 3F, and 4G, respectively. All *Clostridioides difficile* data shown **(B, C)** are representative of a minimum of three independent replicates, apart from *∆cspC/cspC*_*Q516E/T520E*_ at 0.03% TA, which was performed with *n* = 2. The data shown in D were obtained twice, except for CspC-CPD-His_6_:CspA_D1008A/R1036A_, which was resolved via SEC once. The data underlying panels B–D can be found in S1 Data.

Our mutational analyses revealed that disrupting interactions across two distinct regions of the heterodimer interface results in markedly distinct phenotypes: the salt bridge substitutions enhance germinant sensitivity [27] (Fig 3), while the peripheral H-bond substitutions impair germination (Fig 4). However, it remained unclear whether each region plays a functionally distinct, independent role in signaling or whether the regions signal through a linear, i.e., hierarchical, pathway. To distinguish between these possibilities, we combined mutations with the strongest germination phenotypes in a quadruple mutant *∆cspBAC/cspBA*_D1008A/R1036A_:*cspC*_Q516E/T520E_ (*4x mut*) (Figs 2C and 7A). We then used this mutant to assess whether one region functionally constrains the other—in other words, is epistatically dominant—or if the two regions function independently, in parallel [35,36].

If one region exhibits epistasis over the other, then a mutant containing substitutions in both regions would have the same phenotype as the mutant containing substitutions only in the dominant region [35]. Instead, we found that 4× mutant spores exhibited an intermediate germinant phenotype: they germinated better than the germination-null *cspC*_Q516E/T520E_ mutant spores but worse than the hypersensitive *cspA*_D1008A/R1036A_ mutant spores (Fig 7B and 7C). Notably, the 4x mutations resulted in slightly decreased levels of Csps in dormant spores (~30%, S20 Fig). While this modest decrease in Csp levels could partially contribute to reduced germination, the *cspC*_Q516E/T520E_ and *cspA*_D1008A/R1036A_ mutations had no impact on Csp levels in spores (S10B and S11A Figs), and their phenotypes reflect changes in CspC:CspA function rather than abundance. Therefore, the 4× mutant phenotype likely reflects a partial contribution from both regions, suggesting that neither region is epistatically dominant over the other. Thus, signal transduction through these regions of the CspC:CspA heterodimer does not occur in a hierarchical fashion and, instead, the two regions likely signal through independent parallel pathways.

We next analyzed the effect of the 4x substitutions on the stability of the CspC:CspA heterodimer using recombinant proteins. We found that combining the 4× substitutions decreased CspC:CspA heterodimer stability even more than the CspC_Q516E/T520E_ variant, with less untagged CspA_D1008A/R1036A_ being pulled down by the CspC_Q516E/T520E_-CPD-His_6_ variant during affinity purification compared with WT and the constituent CspA or CspC mutants alone (S12B Fig). Furthermore, the 4× mutant heterodimer was less stable in SEC analyses than the CspC_Q516E/T520E_ mutant (Fig 7D). Despite targeting two regions of the CspC:CspA heterodimer, the 4× mutant did not fully disrupt heterodimer formation in vitro (Fig 7D). This finding suggests that the Y361_CspC_:Q1090_CspA_ main chain H-bond contributes to the stability of the CspC:CspA heterodimer (Figs 2C, middle inset and 4A, lower inset). Like the CspC_Q516E/T520E_ mutant, the 4× mutant displayed variability across replicates in the amount of untagged CspA pulled down in the co-affinity purifications and the relative amount of CspC_Q516E/T520E_-CPD-His_6_:CspA_D1008A/R1036A_ heterodimer observed in SEC analyses (S14B, S14D, and S14E Fig). Nevertheless, re-running the SEC-purified 4× mutant (CspC_Q516E/T520E_-CPD-His_6_:CspA_D1008A/R1036A_) complex on SEC resulted in a stable heterodimer (S13B Fig), similar to the CspC_Q516E/T520E_ mutant (Fig 13A), highlighting the stability of the mutant complexes. Accordingly, the 4× mutant exhibited the same chymotrypsin digestion pattern with or without TA and glycine as WT and the previously tested mutants (Figs 6A, 6B, and S18). TA and calcium also had no effect on the elution of the 4× mutant by SEC (S19C Fig).

Collectively, we found no direct correlation between germinant sensitivity and CspC:CspA heterodimer stability in vitro (Fig 7B–7D). Thus, our data suggest that, while the CspC:CspA heterodimer plays a central role in germinant sensing, its stability in vitro does not determine germinant sensitivity. Indeed, the behavior of this complex in vitro may not accurately reflect what occurs in the *C. difficile* spore. These data also support the idea that different regions at the heterodimer interface function independently to mediate germinant signal transduction.

## Discussion

Genetic screens originally implicated the CspC and CspA pseudoenzymes as the germinant and co-germinant receptors in *C. difficile* [17,18], respectively. However, our subsequent structure-function analyses suggested that CspC is involved in sensing both signals during germination [27], so it was unclear how CspC cooperates with CspA to regulate both germinant and co-germinant sensing. Here, we addressed this question by demonstrating that CspC and CspA form a highly stable heterodimer. Our extensive mutational analysis of the CspC:CspA heterodimer interface, informed by the crystal structure of the heterodimer determined here, revealed that the CspC:CspA heterodimer plays a key role in sensing germinant and co-germinant signals in *C. difficile*. Specifically, we uncovered key interaction sites that regulate *C. difficile* germinant sensing: a central dynamic region that controls the sensitivity of *C. difficile* spores to germinant signals and peripheral interactions that are critical for germinant signal transduction. Moreover, our structure-function analyses identified CspA residues that regulate the sensitivity of *C. difficile* spores to TA germinant (Figs 3–5). Thus, both CspC and CspA, rather than the individual proteins [27], determine the sensitivity of *C. difficile* spores to *both* germinant and co-germinant signals because the CspC:CspA complex, rather than the individual proteins, integrates and transduces germinant and co-germinant signals.

While we also demonstrated that CspA forms a homodimer in vitro, our structure-function analyses, guided by our newly determined crystal structure, strongly suggest that disrupting CspA homodimerization has only subtle effects on *C. difficile* TA germinant sensing compared to analogous mutations in the CspC:CspA heterodimer (Figs 4 and 5). Therefore, we propose that CspA homodimerization is less physiologically relevant than CspC:CspA heterodimerization during *C. difficile* spore germination.

**Fig 8:**
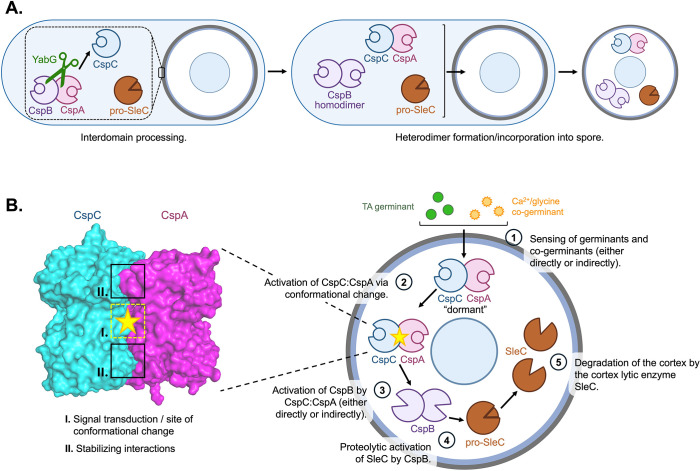
Model for *Clostridioides difficile* germination. **(A)** Model of the *C. difficile* proteins required for germination during sporulation. From left to right: interdomain processing of the CspBA fusion protein by the protease YabG occurs in the spore coat layer [61]; heterodimerization of CspC and CspA and homodimerization of CspB; and loading of key germination proteins into the cortex region of the mature spore [62]. **(B)** Model of a *C. difficile* spore undergoing germination. Sensing of germinant and co-germinant signals (either directly or indirectly) by the CspC:CspA heterodimer (1) leads to the heterodimer adopting an active conformation (2). Inset: CspC:CspA heterodimer space-filling model. Residues at the center of the CspC:CspA heterodimer binding interface (I.) play important roles in transducing germinant and co-germinant signals, while residues at the periphery of the binding interface (II.) help stabilize the complex. Signal transduction by the CspC:CspA heterodimer initiates the activation of CspB (either directly or indirectly) (3). Active CspB then proteolytically activates the cortex-lytic enzyme SleC (4), which will go on to degrade the spore cortex (5), allowing for rehydration of the spore core and resumption of metabolic activity.

Our interrogation of the CspC:CspA binding interface has yielded key insights into the mechanism by which the heterodimer transduces signals during *C. difficile* spore germination. Notably, we found that every amino acid that was targeted in this study was conserved across the five *C. difficile* clades (34 genotypes aligned, S5 Table) [37,38]. This suggests high conservation across these regions of CspC and CspA, highlighting the importance of these residues for *C. difficile* germination. While we previously showed that the CspC residues, Asp429, Arg456, and Gly457—which are unstructured in the CspC monomer—influence the sensitivity of spores to both germinant and co-germinant signals [27], we now show that these residues are part of a central dynamic region where Asp429_CspC_ and Arg456_CspC_ form two salt bridges with residues in CspA (Fig 2C). Similar to our prior analyses of the CspC residues that participate in these interactions [27], we found that mutation of the CspA residues engaged in these salt-bridge interactions hypersensitizes spores to TA germinant (Fig 3). This suggests that the central salt bridges in the CspC:CspA heterodimer normally inhibit germinant and co-germinant signaling in dormant spores. Moreover, both salt bridges are present only in the CspC_C_:CspA_A_ heterodimer, whereas the CspC_D_:CspA_B_ heterodimer contains only one interaction in this region (S7 Fig), further supporting the flexibility in this region. Consistently, B-factor analyses of these structures showed that CspC_D_:CspA_B_ exhibits greater conformational flexibility than CspC_C_:CspA_A_ in the central region and elsewhere in the complex (S21 Fig, left). Together, these structural data, along with prior work [27], suggest that this region undergoes conformational changes during germinant and co-germinant sensing to facilitate signal transduction (Fig 8). We propose that the two heterodimer structures may represent “dormant” versus “active” or “primed” conformational states (Figs 8, S7, and S21) that regulate *C. difficile* germination.

By contrast, the H-bonds at the periphery of the heterodimeric interface appear to be less conformationally dynamic (Fig 2 and S2 Table). Their disruption abrogates germinant sensing and reduces heterodimer formation (Fig 4), suggesting that heterodimerization is crucial for germinant sensing and subsequent signal transduction. Interestingly, we found that the central dynamic region and the peripheral H-bonds function independently during germinant signal transduction (Fig 7B), with neither region exhibiting protein epistasis over the other. These findings suggest a model where the peripheral H-bonds stabilize the pseudoproteases in the heterodimeric state, which is required for the central dynamic region to transduce the germination signal (Fig 8B). Signal transduction through this central region by the CspC:CspA heterodimer then initiates the activation of the CspB protease, which in turn proteolytically activates the cortex lytic enzyme SleC [19,20] (Fig 8), triggering cortex degradation and the resumption of metabolic activity [5,8].

Our discovery of the CspC:CspA heterodimer also suggests an explanation for why CspC requires CspA for its stable incorporation into spores [22,24]. Given the extensive interactions between CspC and CspA revealed by the CspC:CspA crystal structure, we hypothesize that CspA binding to CspC promotes its stable incorporation into spores. Since we were unable to identify mutants that fully disrupt heterodimer formation, it was not possible to test this hypothesis, but our finding that Csp levels are slightly reduced (~30%) in the 4× mutant (S20 Fig) is consistent with this hypothesis, since these four substitutions can decrease CspC:CspA heterodimer formation in vitro (Fig 7).

Regardless, with the identification of the CspC:CspA heterodimer as a key signaling node, our findings raise three key questions: (1) Does the CspC:CspA heterodimer directly sense germinants and co-germinants? (2) What conformational changes occur in the CspC:CspA heterodimer in response to germinant and co-germinant sensing? (3) How does the CspC:CspA heterodimer activate its cognate protease, CspB? Studies of pseudoenzyme function in diverse systems provide a useful framework for considering these questions because pseudoenzymes, like CspC and CspA, often modulate the activity of their cognate enzymes using a variety of mechanisms [39–41].

As for the first question, pseudoenzymes often act as molecular switches that bind small-molecule ligands, which alter their conformation and allow them to activate their cognate enzymes [41]. According to this model, germinant and co-germinant binding to the CspC:CspA heterodimer would cause the conformational changes that trigger germination. While we were unable to detect any effect of the germinants and co-germinants on the CspC:CspA heterodimer formation or stability in vitro (Fig 6), these assays did not measure binding directly. However, detecting direct binding between germinants and their receptors has been challenging even in *B. subtilis*, despite strong genetic evidence that germinants are recognized by nutrient-gated channel-like Ger receptors [15,42,43]. Moreover, these in vitro assays could be missing key regulatory factors found within *C. difficile* spores or the environmental signals necessary for these interactions. For example, the gel-forming GerG protein regulates Csp levels in spores and spore germinant sensitivity [44], so proper CspC:CspA function may depend on GerG. Indeed, the absence of regulatory proteins like GerG could explain why our in vitro analyses of CspC:CspA heterodimer stability do not correlate strongly with our germination phenotypes (Fig 7). We aim to address the involvement of other important proteins, like GerG, during *C. difficile* germination in future studies.

For the second question, our analyses identified key residues in the heterodimer that are likely involved in the conformational changes driving germination. Our crystal structures reveal several conformationally dynamic regions in both the CspC:CspA heterodimer and the CspA homodimer that may contribute to this pathway. Notably, the N-terminal prodomain of CspA is very dynamic in both of the CspC:CspA heterodimers and partially unstructured in two of the chains in the CspA homodimer asymmetric unit, yet structured in the other two chains (S4 and S21 Figs). Interestingly, the N-terminal prodomain of CspA is generated when the YabG protease separates CspB from CspA via CspBA interdomain processing [18,19,22]. Altering the length of this prodomain (due to loss in YabG or mutation) alters *C. difficile*’s response to co-germinant signals [18]. Since the affinity of CspC for CspA is greater following CspBA interdomain processing (S1 Fig), altering CspA’s N-terminal prodomain could impact CspA binding to CspC and thus its signaling function. Intriguingly, a similar subtilisin-like serine protease, PCSK9, uses an intrinsically disordered sequence in its N-terminal prodomain to allosterically regulate its affinity for lipoproteins versus the low-density lipoprotein receptor [45–49]. Future analyses of the role of CspA’s N-terminal prodomain on CspC:CspA heterodimer function could provide further insight into how this complex transduces germinant and co-germinant signals.

Finally, to address the third question, pseudoenzymes frequently regulate their cognate enzymes by forming heterodimeric complexes with their active counterparts [39,40]. Thus, the CspC:CspA heterodimer could, in principle, activate the CspB protease via a “partner swap” whereby one pseudoprotease would dissociate from the heterodimer and interact with CspB to regulate its activity. However, the extreme stability of the CspC:CspA heterodimer in vitro, both in the absence and presence of germinants and co-germinants, makes dissociation of the complex less likely (Figs 4, 6, and 7).

Alternatively, pseudoenzymes can also serve as scaffolds that orchestrate the assembly and spatial organization of signaling complexes [39,40,50]. The germinant receptors in *B. subtilis* form germinosomes, where germination proteins spatially cluster [51], so the CspC:CspA heterodimer may be similarly clustered near key germination proteins like CspB and SleC, potentially in partnership with GerG. By clustering germination components within a defined locus, the system could facilitate the efficient activation of CspB by the “active” conformation of the CspC:CspA heterodimer. Testing this possibility by localizing *C. difficile* germination proteins with the CspC:CspA heterodimer is the subject of further investigation.

The identification of the CspC:CspA heterodimer as a central signaling node during *C. difficile* germination may also inform studies of the germination mechanisms used by other clostridial organisms encoding Csp homologs. Notably, *C. perfringens* and *C. septicum* spores were recently shown to respond to bile acids [52,53], and *C. septicum* CspC homologs have been genetically implicated in regulating bile acid-mediated germination [53]. Therefore, clostridial species may use a similar CspC:CspA heterodimer for inducing germination. Our finding that CspB forms a homodimer in *C. difficile* (Figs 1 and S2), similarly to *C. perfringens* CspB [19], supports the notion that features of the germination machinery may be shared across a wide range of clostridial species. Future analyses of Csps and their interactions across other species will uncover the mechanisms underlying germination in the *Clostridia* and facilitate the development of strategies for inhibiting the germination of diverse clostridial pathogens.

## Materials and methods

### Bacterial strains and growth conditions

*Escherichia coli* DH5α strains are listed in S1 Table and were grown in Luria-Bertani (LB) broth at 37°C with 225 rpm shaking. For protein production, *E. coli* BL21(DE3) strains were grown in autoinduction broth (Terrific broth [Thermo Fisher] supplemented with 0.5% glycerol, 0.05% glucose, and 0.1% α-lactose monohydrate). Media were supplemented with 20 μg/mL chloramphenicol, 100 μg/mL ampicillin, or 30 μg/mL kanamycin as needed.

*C. difficile* strains are listed in S2 Table and were grown on brain heart infusion medium supplemented with 0.5% w/v yeast extract and 0.1% w/v L-cysteine (BHIS) with TA (0.1% w/v; 1.9 mM), thiamphenicol (10–15 μg/mL), kanamycin (50 μg/mL), or cefoxitin (8 μg/mL) as needed. *C. difficile* defined medium (CDDM) was used for constructing complementation strains [54]. Cultures were grown swirling at 37°C under anaerobic conditions using a gas mixture containing 85% N_2_, 5% CO_2_, and 10% H_2_.

### *E. coli* strain construction

All plasmids used in this study are compiled in S1 Table, containing external links to plasmid maps with primer sequences used during cloning. *C. difficile* genes were codon-optimized (co) for *E. coli,* and plasmids were cloned via Gibson assembly and transformed into *E. coli* (DH5α). All plasmids were confirmed with whole plasmid sequencing by Plasmidsaurus using Oxford Nanopore Technology. Confirmed plasmids were transformed into *E. coli* HB101 for conjugation with *C. difficile* or *E. coli* BL21(DE3) for protein expression.

BL21(DE3) *E. coli* strains were designed to encode Csps under the control of a pT7_lac_ promoter (Novagen). CspC-CPD-His_6_ and CspA-His_6_ were expressed in pET22b plasmids containing an ampicillin resistance cassette (Novagen), while untagged CspA was expressed in a pRSFDuet1 plasmid containing a kanamycin resistance cassette (Novagen).

### Recombinant protein affinity purification

BL21(DE3) *E. coli* strains were grown in 20 mL LB with antibiotics as needed, back-diluted 1:1,000 into 1 L autoinduction broth with antibiotics as needed and grown for ~60 hours at 20°C with 225 rpm shaking. Cultures were pelleted, resuspended in 50 mL low imidazole buffer (LIB; 500 mM NaCl, 50 mM Tris-HCl pH 7.5, 15 mM imidazole, 10% glycerol, 2 mM β-mercaptoethanol), and flash frozen in liquid nitrogen. Once thawed, cells were probe sonicated [Branson] in 3 × 45-s rounds at 40% amplitude with 5 min on ice between. The insoluble material was pelleted, and tagged proteins were affinity-purified from cleared lysates using Ni-NTA agarose beads [Qiagen] with gentle rocking at 4°C for 2 hours. The beads were washed three times with LIB before elution with either high imidazole buffer (HIB; 500 mM NaCl, 50 mM Tris-HCl pH 7.5, 200 mM imidazole, 10% glycerol, 2 mM β-mercaptoethanol) or by inducing cleavage of the CPD tag [29,30] with 200 μM inositol hexakisphosphate (InsP_6_, Sigma) in LIB. Samples of whole cells before purification (induced), after lysis and removal of insoluble material (cleared lysate), and elution were analyzed by SDS-PAGE with Coomassie staining.

### Size exclusion chromatography

Affinity (or co-affinity) purified protein was buffer-exchanged into SEC buffer (200 mM NaCl, 10 mM Tris-HCl pH 7.5, 5% glycerol, 1 mM dithiothreitol) and concentrated using an Amicon Ultra-15 30 kDa cutoff centrifugal filter [Millipore Sigma]. Proteins were separated using a Superdex 200 Increase 10/300 GL column [GE] on an AKTA Pure high-pressure liquid chromatography instrument [GE] with the following parameters: 500 μL loop volume, empty loop with 1 mL; 0.2 mL/min flow rate for 1 column volume. Apparent MWs were calculated using a BioRad gel filtration standard. Fractions were collected every 0.5 mL for SDS-PAGE analyses using Coomassie staining or Western blotting.

### CspA and CspC mixing experiments

CspA and CspC were purified as described above, mixed in a 1:1 ratio (w:w) in SEC buffer, and incubated for 2 hours at room temperature. The mixture was analyzed by SEC as described above. SEC fractions were analyzed by SEC followed by Western blotting. Western blots were imaged using LiCOR Odyssey instrument. Blots were quantified in LiCOR Image Studio software and each band was normalized to the sum of all bands in the respective channel.

### Protein purification for crystallography

BL21(DE3) strains #3330 (S1 Table) containing pET22b-CspC-CPD-His_6_ and pRSFDuet1-CspA_F944E_/_Y1092E_ plasmids, and strain #3127 (S1 Table) containing pET22b-CspA-His_6_ were used for crystallography. For the case of the CspA homodimer, Ni-NTA agarose beads were eluted using high imidazole buffer. For the CspA:CspC heterodimer, instead of eluting the proteins using high imidazole, InsP_6_ was used to cleave the C-terminally CPD-His_6_ tag [29,30] from CspC to purify the untagged version of the CspC-CspA heterodimer. Ni-NTA agarose beads were incubated with 200 µM InsP_6_ in LIB buffer with gentle shaking at 4°C overnight. For both protein complexes, after the incubation with high imidazole or InsP_6_, Ni-NTA agarose beads were pelleted, and the supernatant collected. Pooled proteins were concentrated and injected into the Superdex 200 Increase 10/300 GL column. Fractions from the SEC peaks containing the pure CspA homodimer and CspA:CspC heterodimer, respectively, were collected and re-concentrated using Amicon Ultra-15 30 kDa cutoff centrifugal filter [Millipore Sigma].

### Crystallization and structure determination

#### CspA homodimer.

One μL of purified CspA homodimer, concentrated to 10 mg/mL, was mixed 1:1 with reservoir solution containing 0.2 M lithium sulfate, 0.1 M morpholinoethane sulfonic acid pH 6.0, and 20% w/v polyethylene glycol (PEG) 4,000. Vapor diffusion in hanging drops yielded large stacks of rhomboidal plates after two weeks at room temperature. These crystal stacks were harvested and broken up for a microseed stock using glass beads. This microseed stock was diluted 1,000,000-fold in reservoir solution and combined with soluble CspA at 4 mg/mL concentration. These conditions yielded individual rhomboidal plates between 50 and 100 μm across. Crystals were harvested and flash frozen using 20% glycerol as a cryoprotectant. X-ray diffraction data were collected at 100 K using NE-CAT beam line ID-24-C at the Advanced Photon Source, Argonne National Laboratory, at a wavelength of 0.9786 Å. Data were preprocessed with the Xia2 pipeline using the Dials and Aimless modules. The initial structure of the CspA homodimer was phased by molecular replacement in PHASER using the CspC structure from *C. difficile* (PDBID: 6MW4) [27] as an initial search model. The model was manually rebuilt in COOT [55] and refined in PHENIX [56] to a resolution of 3.2 Å. The atomic coordinates and structure factors were deposited to the RCSB Protein Data Bank under the accession number (PDBID): 9PR9.

#### CspC:CspA heterodimer.

100 nL of purified CspC-CspA_EE_ heterodimer, concentrated to 10 mg/mL, was mixed 1:1 with reservoir solution containing 0.2 M magnesium chloride, 0.1 M Tris-HCl pH 8.5, and 20% w/v PEG 8,000. Vapor diffusion in hanging drops yielded rhomboidal plates ~100 μm in length after two weeks at room temperature. Crystals were harvested and flash frozen using 15% ethylene glycol as a cryoprotectant. X-ray diffraction data were collected at 100 K using NE-CAT beam line ID-24-C at the Advanced Photon Source, Argonne National Laboratory, at a wavelength of 0.9786 Å. Data were preprocessed with the Xia2 pipeline [57] using the Dials and Aimless modules. The initial structure of the CspC:CspA heterodimer was phased by molecular replacement in PHASER using the CspC structure from *C. difficile* (PDBID: 6MW4) [27] as an initial search model. The model was manually rebuilt in COOT [55] and refined in PHENIX [56] to a resolution of 3.3 Å. The atomic coordinates and structure factors were deposited to the RCSB Protein Data Bank under the accession number (PDBID): 9PR8.

### C*. difficile* strain construction

Complementation strains were constructed as previously described [27,29,30]. Briefly, mutant *csp* genes were assembled in *pMTL-YN1C* plasmids containing the truncated *pyrE* gene [33,58], cloned into DH5α cells, and sequence-confirmed using Plasmidsaurus. Sequence-confirmed plasmids were transformed into HB101 *E. coli* strains containing the necessary genes for conjugation. HB101 strains containing mutant *csp* complementation plasmids were combined with the associated *∆csp C. difficile* strain to construct the *C. difficile* complementation strains. CDDM media was used to select for recombination of the construct into the *pyrE* locus, which restores the uracil prototrophy. Two independent clones from each conjugation were phenotypically characterized.

### Sporulation and spore purification

Sporulation and spore purification were performed as previously described [27,44]. Briefly, *C. difficile* strains were streaked from frozen glycerol stocks onto BHIS plates and grown overnight. Liquid BHIS cultures were grown until they reached an OD_600_ between 0.35 and 0.75. To induce sporulation, 100–120 μL of these cultures were spread onto 70:30 agar plates. Spores were collected from 70:30 plates a minimum of 60 hours later and resuspended in ice-cold sterile water. To confirm proper levels of sporulation, a small sample was removed and examined via phase-contrast microscopy. Spore samples were washed with ice-cold water 5–8 times and incubated on ice overnight. The next day, the samples were treated with DNAse I [New England Biolabs] for 1 hr at 37°C. After treatment, the spores were washed 1–2 times in ice-cold water and then purified on a 20%−50% Histodenz [Sigma Aldrich] gradient. After 2 more washes with water, spore purity was confirmed using phase-contrast microscopy. The samples were stored at 4°C until use.

### OD_600_ kinetics assay

The OD_600_ kinetics assay protocol was adapted from previous works [11,27]. Spores were resuspended in BHIS media to an OD_600_ of 0.89 (~7 × 10^5^ spore-forming units/μL), and 180 µL were added into wells of a 96-well flat-bottom tissue culture plate [Falcon] for each condition tested. The spores were exposed to different concentrations of TA (1%, 18.6 mM; 0.5%, 9.3 mM; 0.25%, 4.7 mM; 0.125%, 2.3 mM; 0.06%, 1.2 mM; 0.03%, 0.6 mM) or water (untreated) to a final volume of 200 µL per well (final spore OD_600_ of 0.8). The OD_600_ was measured every 3 min using a Synergy H1 microplate reader [Biotek] at 37°C with constant shaking between readings. Readings were subtracted by background OD_600_ of spore-free media, and the values were normalized to the first measurements (time 0) and plotted as OD_600_ versus time (S8 Fig). Germination (arbitrary units) for each strain was calculated by first subtracting each of OD_600_ values from 1 to invert the curves (S8 Fig). The area under the inverted curves was plotted for each strain as a bar graph (S8 Fig). Area under the curve calculations were performed using Prism. All germination assays were initially performed using all 7 concentrations of TA to determine the concentration that best displays a given germinant sensitivity phenotype. This assay was performed for each strain at a minimum of three biological replicates, with three individually prepared spore isolates.

For OD_600_ kinetics assays using TA and calcium, spores were resuspended in 50 mM HEPES buffer + 100 mM NaCl to an OD_600_ of 0.89 and aliquoted into wells of a 96-well plate. The spores were exposed to 0.25% (4.7 mM) TA and different concentrations of CaCl_2_ (0.6 mM or 1.25 mM). The rest of the assay was performed as described above.

### Western blot analysis

Spore samples for immunoblotting were prepared as previously described [18,22,27]. Spores were diluted to an OD_600_ of 1.4 in 100 μL of sterile water (1.5 × 10^8^ spore-forming units), pelleted, and resuspended in 50 μL EBB buffer (8 M urea, 2 M thiourea, 4% (w/v) SDS, 2% (v/v) β-mercaptoethanol). The samples were boiled for 20 min with vigorous vortexing during incubation, pelleted, and resuspended. Final sample buffer with bromophenol blue was added to 1X to stain the sample. To maximally solubilize spore proteins, the samples were boiled again for 5–10 min, vortexed vigorously, and pelleted. The samples were resolved by 12% SDS-PAGE gels and transferred to Millipore Immobilon-FL PVDF membranes. The membranes were blocked with Odyssey Blocking Buffer with 0.1% (v/v) Tween 20 and probed with mouse monoclonal anti-CspC [22] or anti-SleC [19] and/or rabbit polyclonal anti-CspB [19] or anti-CspA that was raised against SEC-purified CspA-His_6_ in rabbits (CoCalico Biologicals). The anti-CspC and anti-CspA antibodies were used at 1:2,000 dilutions, the anti-CspB antibody was used at 1:3,000, and the anti-SleC antibody was used at 1:5,000. The membranes were probed with primary antibodies for 4 hours, washed 3× with PBS with 1.5× Tween 20, and probed with IRDye 680CW and 800CW infrared dye-conjugated secondary antibodies at 1:12,000. After incubation for 1hour in the dark, the membranes were washed twice with PBS with 1.5× Tween 20, and secondary antibody infrared fluorescence emissions were visualized using an Odyssey LiCOR CLx. The results shown are representative of three independently prepared spore isolates.

For western blot analysis of limited proteolysis samples, the samples were probed with mouse anti-CspC antibody and rabbit anti-CPD antibody [59] at a 1:2,000 dilution. The rest of the assay was performed as described above.

### Limited proteolysis

Limited proteolysis of purified *C. difficile* proteins was performed as previously described in *Rohlfing and colleagues [27]*. Purified *C. difficile* Csp proteins were diluted to 15 µM in 10 mM Tris pH 7.5 buffer. Twenty-four µL of the protein solution was aliquoted into 0.2 mL PCR tubes. A 1 mg/mL chymotrypsin stock solution was diluted in 10 mM Tris pH 7.5 buffer to generate 10-fold dilutions. 1 µL of the appropriate chymotrypsin dilution was added to the protein solution to achieve the indicated concentration of chymotrypsin. 1 µL of 10 mM Tris pH 7.5 buffer was added to the protein samples as an untreated control. The protein and chymotrypsin solutions were incubated at 37°C for 1 hour. Chymotrypsin activity was then quenched with the addition of NuPAGE 4× LDS Sample Buffer [Invitrogen] and boiled for 3 min at 98°C. The samples were resolved on a 15% SDS-PAGE gel and visualized using Coomassie staining.

For limited proteolysis experiments with the addition of TA and glycine, chymotrypsin was diluted as above, while the proteins were diluted to a final concentration of 7.5 µM in 10 mM Tris buffer. Twenty-three µL of the protein solution was added to the 0.2 mL PCR tubes. A 10% (186 mM) TA stock solution and a 200 mM glycine stock solution were diluted to 25 mM in 10 mM Tris pH 7.5 buffer. 1 µL of the TA/glycine solution was added to the indicated protein samples for final concentrations of 1 mM TA and 1 mM glycine. 1 µL of the appropriate chymotrypsin dilution was added to the protein solution to achieve the indicated concentration of chymotrypsin. Incubation, protease quenching, resolution, and visualization were performed as described above.

### Sequence alignment

CspA and CspC sequences were retrieved from Lewis *and colleagues* [37] and Knight *and colleagues* [38] (S5 Table) and aligned with default parameters using MUSCLE [60]. Alignments were manually inspected in Geneious Prime 2025.2.2.

### Data visualization

All graphs were prepared using GraphPad Prism. Gels and blots were imaged using a LiCOR Odyssey instrument. Protein structure visualization was performed using Pymol.

## Supporting information

S1 Table CspA homodimer and CspC:CspA heterodimer data collection and refinement. CspA_EE_, CspA_F944E-Y1092E_ (*cspBA* fusion gene numbering), and CspA_F363E-Y511E_ (YabG-cleaved CspA numbering).
(DOCX)

S2 Table PISA interface analyses of CspC-CspA heterodimer and CspA homodimer. PISA interface analysis identified hydrogen bond and salt bridge interactions at the hetero- and homodimeric interfaces. The heterodimer has a more extensive interface, with 20 hydrogen bonds and five salt bridges across the interface, whereas the CspA homodimer has 15 hydrogen bonds and two salt bridges across the interface. Amino acid numbering based on YabG-cleaved CspA. “*cspBA* numbering” based on *cspBA* fusion gene.
(DOCX)

S3 Table *Escherichia coli* strains used in this study. Amino acid numbering based on YabG-cleaved CspA. “*cspBA* numbering” based on *cspBA* fusion gene. All *Clostridioides difficile* genes expressed in *E. coli* were designed with codon optimization for *E. coli* expression.
(DOCX)

S4 Table *Clostridioides difficile* strains used in this study. *cspBA* numbering based on *cspBA* fusion gene.
(DOCX)

S5 Table *Clostridioides difficile* clinical isolates used for *cspC* and *cspA* sequence alignment. Strain name and clade number (1–5) for C. *difficile* strains [37,38] from Lewis *and colleagues* [37] and Knight *and colleagues* [38] used for sequence alignment of *cspC* and *cspA* genes.
(DOCX)

S1 Fig CspBA binds CspC with lower affinity than the CspA variant generated by interdomain processing. Coomassie stain of co-affinity purifications using CspC-CPD-His_6_ as the bait and untagged CspBA or CspB and CspA as the prey. +, induced fraction; CL, cleared lysate; E, elution. The data shown are representative of two independent replicates. The data shown are representative of two independent replicates. The raw gel image can be found in S1 Raw Images.
(TIF)

S2 Fig CspA and CspB form homodimers, but CspC does not. Coomassie stain of co-affinity purification analyses using CPD-His_6_-tagged CspA, CspB, or CspC as the bait alongside their respective untagged Csps as prey. GFP-CPD-His_6_ is the control bait. All data shown are representative of three replicates. All data shown are representative of three replicates. The raw gel images can be found in S1 Raw Images.
(TIF)

S3 Fig Csp domain boundaries. Bounds of the prodomains, subtilase domains, and jellyroll domains of *C. perfringens* CspB [19] (top), and *Clostridioides difficile* CspC and CspA (center and bottom, respectively). Bounds are representative of every structure in this work.
(JPG)

S4 Fig Asymmetric unit of CspA. **(A, B)** The two CspA homodimers that crystallized within the asymmetric unit (PDB 9PR9). The subtilase domains are shown in gray, jellyroll domains in green, and prodomains in purple. The structure used for all other CspA homodimer figures in this manuscript is shown in (A). **(C)** Overlay of the two CspA homodimer structures (A and B). The A homodimer is shown in light pink (CspA_B_) and magenta (CspA_A_), the B homodimer is shown in gray.
(TIF)

S5 Fig Crystal contacts with symmetry mates stabilize the CspA A and C protomer prodomain. The prodomains of the A and C protomers in the CspA asymmetric unit are better resolved than their counterparts in the B and D protomers. As shown in the insets, the A protomer’s prodomain makes contacts with C and D symmetry mates in the crystal lattice. The N terminus of the A prodomain is stabilized by contacts with the subtilase domain of a C protomer (bottom left inset), while the C terminus of the A prodomain is stabilized by contacts with the D protomer jellyroll domain (top right inset). Because of the screw axis and crystal packing, the B and D protomers lack these contacts with symmetry mates. Only protomers A and B of the asymmetric unit are shown for simplicity.
(JPG)

S6 Fig Size exclusion chromatography analyses of the protein variant used for CspC:CspA crystallization. Size exclusion chromatography analysis of CspC-CPD-His_6_-CspA_F944E/Y1092E_ (CspC:CspA_EE_) co-affinity purification. The dashed line indicates the separation between the two peaks. The data shown are representative of a minimum of three independent replicates. The data underlying this figure can be found in S1 Data.
(TIF)

S7 Fig Asymmetric unit of CspC:CspA. **(A, B)** The two CspC:CspA heterodimers that crystallized within the asymmetric unit (PDB 9PR8). CspC on the left, CspA on the right. The subtilase domains are shown in gray, jellyroll domains in green, and prodomains in purple. The structure used for all other CspC:CspA heterodimer figures in this manuscript is shown in (A). **(C)** Overlay of the two CspC:CspA heterodimer structures (A and B). The A heterodimer is shown in cyan (CspC_C_) and magenta (CspA_A_); the B heterodimer is shown in gray. (Inset) Two salt bridges between CspC and CspA form within the CspC_C_:CspA_A_ heterodimer. The CspC_C_ D429:CspA_A_ R1036 salt bridge interaction is a distance of 3.6 Å, and the CspC_C_ R456:CspA_A_ D1008 is 2.9 Å. A single salt-bridge forms between CspC_D_ D429:CspA_B_ R1036 (distance of 3.1 Å). The CspC_D_ R456 residue is unstructured, and no interaction exists between CspC_D_ R456 and CspA_B_ D1008.
(TIF)

S8 Fig Schematic of OD_600_ drop assay data presentation. Spore germination was assessed using optical density (OD_600_). Spores were exposed to germinant and their OD_600_ was measured at 3-min intervals for 1.5 h. After the OD_600_ of blank wells (sterile medium) was subtracted, the values were normalized to the first measurements (time 0), and the values were plotted as OD_600_ versus time (left). (1) The resulting curves were inverted by subtracting each of the values from 1 (center). (2) The area under the inverted curves (center) was plotted for each strain as a bar graph (right). Area under the curve calculations were performed using Prism. All germination assays were performed at the presented concentrations of TA to determine the concentration that best displays a given germinant sensitivity phenotype. The data underlying this figure can be found in S1 Data.
(TIF)

S9 Fig Mutation of two salt bridges at the CspC:CspA heterodimer interface increases the sensitivity of *Clostridioides difficile* spores to the co-germinant calcium. Gemination levels based on the change in optical density (OD_600_) of purified spores suspended in buffer containing 50 mM HEPES buffer and 100 mM NaCl and the indicated concentration of calcium over time following the addition of 4.7 mM taurocholate (TA). Germination in arbitrary units (AU) was calculated using the area below inverted OD_600_ curves (S8 Fig). *cspC* complementation mutants were constructed in a *∆cspC* background **(A)**. *cspBA* complementation mutants were constructed in a *∆cspBA* background **(B)**. CspA residue numbers are based on the full-length CspBA fusion protein. Statistical significance relative to WT was determined using a one-way ANOVA and Dunnett’s multiple comparisons test. **** *p* < 0.0001, *** *p* < 0.001, ** *p* < 0.01, * *p* < 0.1. It should be noted that the specific effect of glycine co-germinant on the germination of the indicated mutants was not assayed due to the complicating effects of internal calcium being released in the form of calcium dipicolinic acid (Ca-DPA). The released calcium can synergize with glycine to promote germination [11]. The data underlying the panels in this figure can be found in S1 Data.
(TIF)

S10 Fig Mutation of two salt bridges at the CspC:CspA heterodimer interface does not impact Csp levels in mature spores. **(A, B)** western blot analyses of Csp levels in mutant spores. Multiple isoforms of CspA are observed. * indicates a non-specific band. SleC was used as a load control. The data shown are representative of a minimum of three independent replicates. The raw gel images can be found in S1 Raw Images.
(TIF)

S11 Fig Mutations in peripheral H-bonds of the CspC:CspA heterodimer interface do not impact Csp levels in mature spores. **(A, B)** western blot analyses of Csp levels in mutant spores. ∆*cspBAC/3xAla* = triple-Ala substitution *cspBA*_*R896A*_*-cspC*_*Q516A/T520A*_, ∆*cspBAC/3xGlu* = triple-Glu substitution *cspBA*_*R896E*_*-cspC*_*Q516A/T520E*_. Multiple isoforms of CspA are observed. * indicates a non-specific band. SleC was used as a load control. The data shown are representative of a minimum of three independent replicates. The raw gel images can be found in S1 Raw Images.
(TIF)

S12 Fig Inputs for CspC-CPD-His6 + CspA SEC analyses. **(A, B)** SDS-PAGE of CspC-CPD-His_6_ co-affinity purification inputs for SEC purification, stained with Coomassie. Q516A/T520A represents CspC_Q516A/T520A_-CPD-His_6_ with untagged WT CspA; Q516A/T520E represents CspC_Q516E/T520E_-CPD-His_6_ with untagged WT CspA; 3xAla represents CspC_Q516A/T520A_-CPD-His_6_ with untagged CspA_R896A_; and 3xGlu represents CspC_Q516E/T520E_-CPD-His_6_ with untagged CspA_R896E_ (A). Inputs correspond to SEC traces shown in Fig 4E–4G, 4K, and 4L. (B) CspBAC 4× mut corresponds to CspC_Q516E/T520E_-CPD-His_6_ with untagged CspA_D1008A/R1036A_; CspA DA/RA represents WT CspC-CPD-His_6_ with untagged CspA_D1008A/R1036A_; and CspC QE/TE represents CspC_Q516E/T520E_-CPD-His_6_ with untagged WT CspA. Inputs correspond to the SEC traces shown in Fig 7D. CspA/total (%) = 100 × [CspA signal intensity/(CspA + CspC signal intensities)]. The data shown are representative of a minimum of two independent replicates. The raw gel images can be found in S1 Raw Images.
(TIF)

S13 Fig Re-run of mutant CspC:CspA heterodimer complexes using SEC shows the complexes are stable when formed. Size exclusion chromatography (SEC) analysis of the CspC_Q516E/T520E_-CPD-His_6_:CspA **(A)** and CspC_Q516E/T520E_-CPD-His_6_:CspA_D1008A/R1036A_ **(B)** co-affinity purifications. The dashed line indicates the separation between the two peaks. (A and B insets) Analysis of the stability of SEC-purified CspC-CPD-His_6_:CspA mutant complexes. The complexes were purified from the 12.0 to 13.0 mL fraction and then re-analyzed using SEC. The data underlying the panels in this figure can be found in S1 Data.
(TIF)

S14 Fig Variability in co-affinity purification and SEC. (A–D) Size exclusion chromatography analyses of WT and mutant CspC-CPD-His6:CspA co-affinity purifications. SEC replicates from three independent co-affinity purifications. **(E)** (left) Inputs for replicates 1 and 2 run on SDS-PAGE and stained with Coomassie. (right) Quantification of the percentage of CspA from SEC inputs. CspA/total (%) = 100 × [CspA signal intensity/(CspA + CspC signal intensities)]. The data underlying panels A–D and E (right) can be found in S1 Data. The raw gel image in panel E (left) can be found in S1 Raw Images.
(TIF)

S15 Fig Mutations in the CspA homodimer twin H-bond networks do not impact Csp levels in mature spores. Western blot analyses of Csp levels in mutant spores. ∆*cspBA/3xAla* = *∆cspBA/cspBA*_*R896A/Q1094A/T1098A.*_ Multiple isoforms of CspA are observed. * indicates a non-specific band. SleC was used as a load control. The data shown are representative of a minimum of three independent replicates. The raw gel image can be found in S1 Raw Images.
(TIF)

S16 Fig Effect of mutations in the CspA homodimer hydrogen bond network on CspC:CspA heterodimer formation. Size exclusion chromatography analysis of CspC-CPD-His_6_ and CspA variant co-affinity purifications. The dashed line indicates the separation between the two peaks. All data shown are representative of two independent replicates. The data underlying the panels in this figure can be found in S1 Data.
(TIF)

S17 Fig CspC-CPD-His_6_ tag degradation during limited proteolysis analyses. Western blot of limited proteolysis analyses of purified CspC-CPD-His_6_:CspA heterodimer using the indicated concentrations of chymotrypsin and anti-CspC or anti-CPD antibodies. The raw gel images in this figure can be found in S1 Raw Images.
(TIF)

S18 Fig Substitutions at the CspC:CspA interface do not influence its susceptibility to protease degradation with and without germinants and co-germinants. Limited proteolysis analyses of purified CspC-CPD-His_6_:CspA heterodimer mutants in the presence and absence of the indicated concentrations of taurocholate (TA) and glycine (Gly). Cleavage of the complexes at 40 μg/mL chymotrypsin is due to digestion of the CPD-His_6_ tag (S17 Fig). The raw gel images in this figure can be found in S1 Raw Images.
(TIF)

S19 Fig Germinants and co-germinants have no influence on the stability of the CspC:CspA heterodimer interface mutants. Size exclusion chromatography analyses of mutant CspC-CPD-His_6_:CspA co-affinity purifications in the presence or absence of the indicated concentrations of TA and CaCl_2_. The data underlying the panels in this figure can be found in S1 Data.
(TIF)

S20 Fig The CspC:CspA 4× mutant has decreased levels of Csps in purified spores. **(A)** western blot analyses of Csp levels in mutant spores. ∆*cspBAC/4X mut* = *∆cspBAC/cspBA*_*D1008A/R1036A*_*-cspC*_*Q516E/T520E*_ Multiple isoforms of CspA are observed. Multiple isoforms of CspA are observed. * indicates a non-specific band. SleC was used as a load control. **(B)** Quantification of western blot analyses. Protein signal intensities were normalized to the SleC loading control intensities. Statistical significance relative to WT was determined using a one-way ANOVA and Dunnett’s multiple comparisons test. ** *p* < 0.01, * < 0.1. The data shown are representative of a minimum of three independent replicates. The raw gel image in panel A can be found in S1 Raw Images. The data underlying panel B can be found in S1 Data.
(TIF)

S21 Fig The CspC/CspA heterodimer structure is both more complete and more conformationally dynamic than the CspA homodimer. Protomers for the CspC:CspA heterodimer (left) and the CspA homodimer (right) colored and rendered by B-factor in ChimeraX reveal dynamic regions across the complexes. The CspC protomer’s subtilase and prodomains display high B-factors relative to the rest of the complex. Likewise, the CspA prodomain exhibits high B-factors in both the hetero- and homodimer. Interestingly, the CspC/CspA heterodimer displays a wider B-factor range than does the CspA homodimer. Furthermore, the two CspC:CspA heterodimers in the asymmetric unit exhibit strikingly different B-factor distributions. The C/A heterodimer has a more ordered CspA protomer than does the D/B heterodimer. On the other hand, B-factor distributions are generally similar across the two CspA asymmetric unit homodimers. The red box indicates the regions containing the central salt bridges of the CspC:CspA heterodimer (Figs 2C and 3A).
(JPG)

S1 Data Raw absorbance values for SEC analyses, raw OD600 reads for OD-drop assays, and blot quantifications. (XLSX)

S1 Raw Images Original, uncropped Coomassie and Western blot images. (DOCX)

## Abbreviations

CDDM: *C. difficile* defined medium
CPD: cysteine protease domain
MWs: molecular weights
PEG: polyethylene glycol
SEC: size exclusion chromatography
TA: taurocholate
VDAL: Val-Asp-Ala-Leu
