# Supplementary material for: The CspC:CspA heterodimer transduces germinant and co-germinant signals during Clostridioides difficile spore germination
Source: PLoS Biol. 2026 Feb 2;24(2):e3003610. doi: 10.1371/journal.pbio.3003610 (PMC12880746; doi:10.1371/journal.pbio.3003610)
Supplement: S1 Table — CspAEE, CspAF944E-Y1092E (cspBA fusion gene numbering), and CspAF363E-Y511E (YabG-cleaved CspA numbering). (DOCX) [file pbio.3003610.s001.docx]

| **Supplemental Table S1. Crystallographic data collection and refinement statistics*** | | |
| --- | --- | --- |
| **Construct (PDB ID)** | **CspA homodimer (9PR9)** | **CspC-CspA_EE_ (9PR8)** |
| **Data Collection** |  |  |
| Resolution range (Å) | 100.6  - 3.22 (3.3  - 3.22) | 99.92  - 3.35 (3.45  - 3.35) |
| Space group | P 2_1_ 2_1_ 2_1_ | P 2_1_ 2_1_ 2_1_ |
| Unit cell (Å)  α (°) | 125.98 131.93 155.327  90 | 71.143 105.505 311.325  90 |
| Multiplicity | 7.9 (8.2) | 12.3 (11.9) |
| Completeness (%) | 98.23 (86.37) | 99.67 (97.65) |
| Mean I/sigma(I) | 3.1 (0.6) | 3.6 (0.8) |
| R-merge | 0.64 (3.83) | 0.87 (4.21) |
| R-meas | 0.68 (4.09) | 0.91 (4.39) |
| R-pim | 0.24 (1.41) | 0.26 (1.25) |
| CC_1/2_ | 0.97 (0.33) | 0.97 (0.37) |
|  |  |  |
| **Refinement** |  |  |
| Reflections used in refinement | 41835 (2572) | 34565 (2745) |
| Reflections used for R-free | 1991 (122) | 1733 (145) |
| R-work | 0.2724 (0.3816) | 0.2457 (0.3531) |
| R-free | 0.3190 (0.4054) | 0.2777 (0.3761) |
| Number of non-hydrogen atoms | 15717 | 15999 |
| macromolecules | 15717 | 15997 |
| ligands | 0 | 0 |
| solvent | 0 | 2 |
| Protein residues | 2076 | 2165 |
| RMS_bonds_ (Å) | 0.005 | 0.011 |
| RMS_angles_ (°) | 1.08 | 1.36 |
| Average B-factor | 67.75 | 81.22 |
| macromolecules | 67.75 | 81.23 |
| ligands |  |  |
| solvent |  | 68.84 |
| ***Values in parentheses are for highest-resolution shell.** | | |

**Supplemental Table 1.** **CspA homodimer and CspC:CspA heterodimer data collection and refinement.** CspA_EE_ = CspA_F944E-Y1092E_ (*cspBA* fusion gene numbering), and CspA_F363E-Y511E_ (YabG-cleaved CspA numbering).
