# Supplementary material for: The CspC:CspA heterodimer transduces germinant and co-germinant signals during Clostridioides difficile spore germination
Source: PLoS Biol. 2026 Feb 2;24(2):e3003610. doi: 10.1371/journal.pbio.3003610 (PMC12880746; doi:10.1371/journal.pbio.3003610)
Supplement: S2 Table — PISA interface analysis identified hydrogen bond and salt bridge interactions at the hetero- and homodimeric interfaces. The heterodimer has a more extensive interface, with 20 hydrogen bonds and five salt bridges across the interface, whereas the CspA homodimer has 15 hydrogen bonds and two salt bridges across the interface. Amino acid numbering based on YabG-cleaved CspA. “cspBA numbering” based on cspBA fusion gene. (DOCX) [file pbio.3003610.s002.docx]

**Supplemental Table 2.** **PISA interface analyses of CspC-CspA heterodimer and CspA homodimer.**

| **CspC:CspA Heterodimer** | | | | |
| --- | --- | --- | --- | --- |
|  | Chain:Residue # [Atom] | Distance (Å) | Chain:Residue # [Atom] | CspBA numbering |
| Hydrogen Bonds | | | | |
| 1 | C:ARG 510[ NE ] | 3.80 | A:GLN  55[ O  ] | 636 |
| 2 | C:ARG 510[ NH2] | 3.45 | A:LEU  56[ O  ] | 637 |
| 3 | C:ARG 510[ NH2] | 3.66 | A:ILE  59[ O  ] | 640 |
| 4 | C:ASN 262[ ND2] | 2.85 | A:SER 256[ OG ] | 837 |
| 5 | C:TYR 521[ OH ] | 3.49 | A:ASN 338[ OD1] | 919 |
| 6 | C:GLN 516[ NE2] | 3.33 | A:GLU 363[ O  ] | 944 |
| 7 | C:THR 520[ OG1] | 2.90 | A:GLY 365[ O  ] | 946 |
| 8 | C:LYS 528[ NZ ] | 3.54 | A:TYR 389[ OH ] | 970 |
| 9 | C:LYS 319[ NZ ] | 3.17 | A:VAL 549[ O  ] | 1130 |
| 10 | C:TYR 337[ OH ] | 2.93 | A:LEU 550[ O  ] | 1131 |
| 11 | C:ILE  50[ O  ] | 3.13 | A:ARG 265[ NH2] | 846 |
| 12 | C:VAL  52[ O  ] | 3.83 | A:ARG 265[ NH2] | 846 |
| 13 | C:ALA  53[ O  ] | 3.86 | A:ARG 265[ NE ] | 846 |
| 14 | C:TYR 361[ O  ] | 2.76 | A:GLN 509[ NE2] | 1090 |
| 15 | C:GLY 367[ O  ] | 2.69 | A:THR 517[ OG1] | 1098 |
| 16 | C:ASP 429[ OD2] | 3.39 | A:ARG 455[ NH1] | 1036 |
| 17 | C:ASP 429[ OD2] | 3.56 | A:ARG 455[ NH2] | 1036 |
| 18 | C:LEU 511[ O  ] | 3.39 | A:ASN 338[ ND2] | 919 |
| 19 | C:GLN 516[ OE1] | 2.65 | A:ARG 315[ NH2] | 896 |
| 20 | C:THR 520[ OG1] | 3.06 | A:ARG 315[ NH1] | 896 |
| Salt Bridges | | | | |
| 1 | C:ARG 456[ NH1] | 3.95 | A:ASP 427[ OD1] | 1008 |
| 2 | C:ARG 456[ NH2] | 3.84 | A:ASP 427[ OD1] | 1008 |
| 3 | C:ARG 456[ NH2] | 2.87 | A:ASP 427[ OD2] | 1008 |
| 4 | C:ASP 429[ OD2] | 3.39 | A:ARG 455[ NH1] | 1036 |
| 5 | C:ASP 429[ OD2] | 3.56 | A:ARG 455[ NH2] | 1036 |

| **CspA Homodimer** | | | | | |
| --- | --- | --- | --- | --- | --- |
|  | CspBA numbering | Chain:Residue # [Atom] | Distance (Å) | Chain:Residue # [Atom] | CspBA numbering |
| Hydrogen Bonds | | | | | |
| 1 | 846 | B:ARG 265[ NH2] | 3.11 | A:GLN  55[ OE1] | 636 |
| 2 | 1098 | B:THR 517[ OG1] | 2.77 | A:GLY 365[ O  ] | 946 |
| 3 | 896 | B:ARG 315[ NH1] | 3.81 | A:GLN 513[ O  ] | 1094 |
| 4 | 896 | B:ARG 315[ NH2] | 2.57 | A:GLN 513[ OE1] | 1094 |
| 5 | 896 | B:ARG 315[ NH1] | 3.13 | A:THR 517[ OG1] | 1098 |
| 6 | 970 | B:TYR 389[ OH ] | 3.88 | A:VAL 549[ O  ] | 1130 |
| 7 | 899 | B:LYS 318[ NZ ] | 3.27 | A:GLU 553[ OE2] | 1134 |
| 8 | 915 | B:VAL 334[ O  ] | 2.88 | A:HIS 554[ NE2] | 1135 |
| 9 | 919 | B:ASN 338[ O  ] | 3.81 | A:HIS 555[ N  ] | 1136 |
| 10 | 920 | B:TYR 339[ OH ] | 3.85 | A:HIS 556[ ND1] | 1137 |
| 11 | 946 | B:GLY 365[ O  ] | 3.15 | A:THR 517[ OG1] | 1098 |
| 12 | 1094 | B:GLN 513[ O  ] | 3.69 | A:ARG 315[ NH1] | 896 |
| 13 | 1094 | B:GLN 513[ OE1] | 2.30 | A:ARG 315[ NH2] | 896 |
| 14 | 1098 | B:THR 517[ OG1] | 2.95 | A:ARG 315[ NH1] | 896 |
| 15 | 1131 | B:LEU 550[ O  ] | 3.36 | A:ASN 338[ ND2] | 919 |
| Salt Bridges | | | | | |
| 1 | 899 | B:LYS 318[ NZ ] | 3.80 | A:GLU 553[ OE1] | 1134 |
| 2 | 899 | B:LYS 318[ NZ ] | 3.27 | A:GLU 553[ OE2] | 1134 |

PISA interface analysis identified hydrogen bond and salt bridge interactions at the hetero- and homodimeric interfaces. The heterodimer has a more extensive interface, with twenty hydrogen bonds and five salt bridges across the interface, whereas the CspA homodimer has fifteen hydrogen bonds and two salt bridges across the interface. Amino acid numbering based on YabG-cleaved CspA. “*cspBA* numbering” based on *cspBA* fusion gene.
