# Supplementary material for: The CspC:CspA heterodimer transduces germinant and co-germinant signals during Clostridioides difficile spore germination
Source: PLoS Biol. 2026 Feb 2;24(2):e3003610. doi: 10.1371/journal.pbio.3003610 (PMC12880746; doi:10.1371/journal.pbio.3003610)
Supplement: S3 Table — Amino acid numbering based on YabG-cleaved CspA. “cspBA numbering” based on cspBA fusion gene. All Clostridioides difficile genes expressed in E. coli were designed with codon optimization for E. coli expression. (DOCX) [file pbio.3003610.s003.docx]

**Supplemental Table 3. *E. coli* strains used in this study.**

| Lab Strain # | Strain back-ground | Genotype + plasmid carried | *cspBA* numbering | Source/ Reference | Link to plasmid sequence |
| --- | --- | --- | --- | --- | --- |
| 41 | DH5α | F– Φ80*lacZ*ΔM15 Δ(*lacZYA-argF*) U169 *recA1 endA1 hsdR17* (rK^–^, mK^+^) *phoA supE44* λ– *thi-1 gyrA96 relA1* |  | D. Cameron |  |
| 531 | HB101 | *F– mcrB mrr hsdS20(rB–mB–) recA13 leuB6 ara-13 proA2 lavYI galK2 xyl-6 mtl-1 rpsL20* carrying pRK24 |  | C. Ellermeier |  |
|  | BL21(DE3) | *fhuA2* [*lon*] *ompT* gal (λ DE3) [*dcm*] ∆*hsdS* λ DE3 = λ sBamHIo ∆ERI-B *int*::(*lacI*::P*lacUV5*::T7 gene1) i21 ∆*nin5* |  | C2527 NEB |  |
| 3127 | BL21(DE3) | pET22b *cspA*-His_6_ |  | This study | <https://benchling.com/s/seq-Lpyq87HVkCpwvocrw5mA> |
| 3179 | BL21(DE3) | pET22b cspB-His_6_ |  | This study | <https://benchling.com/ioana-stoica/f/lib_fml9MIsK-sequence-verified-plasmids/seq_Rj4uo05c-pet22b_cspbsr_his/edit> |
| 981 | BL21(DE3) | pET22b *cspC*-His_6_ |  | [1] |  |
| 982 | BL21(DE3) | pET22b *cspC*-CPD-His_6_ |  | This study | <https://benchling.com/s/seq-W2G3f2JJxHxhREANe1KA?m=slm-TGr0hMCIM2jLmUb1exvP> |
| 3125 | DH5α | pET22b *cspA*-CPD-His_6_ |  | This study | <https://benchling.com/s/seq-dgQ8goVh1drorLLQXPQu> |
| 3175 | DH5α | pET22b *cspB*-CPD-His_6_ |  | This study | <https://benchling.com/ioana-stoica/f/lib_fml9MIsK-sequence-verified-plasmids/seq_PX2pDgFK-pet22b_cspbsr_cpd-his/edit> |
| 196 | DH5α | pET22b GFP-CPD-His_6_ |  | [2] |  |
| 3028 | DH5α | pRSFDuet1 *cspA* |  | This study | <https://benchling.com/s/seq-1dBY4omP3AXq53vmqs8G> |
| 3026 | DH5α | pRSFDuet1 *cspB* |  | This study | <https://benchling.com/s/seq-70ZFZwG5oAKEz1Cxswdn> |
| 3177 | DH5α | pRSFDuet1 *cspC* |  | This study | [https://benchling.com/ioana-stoica/f/lib_fml9MIsK-sequence-verified-plasmids/seq_Z0miFevW-prsfduet1_cspC/edit](https://benchling.com/ioana-stoica/f/lib_fml9MIsK-sequence-verified-plasmids/seq_Z0miFevW-prsfduet1_cspc-untagged/edit) |
| 3165 | BL21(DE3) | pRSFDuet1 *cspA* + pET22b *cspA*-CPD-His_6_ |  | This study | 3028 + 3125 |
| 3181 | BL21(DE3) | pRSFDuet1 *cspA* + pET22b *cspB*-CPD-His_6_ |  | This study | 3028 + 3175 |
| 3037 | BL21(DE3) | pRSFDuet1 *cspA* + pET22b *cspC*-CPD-His_6_ |  | This study | 3028 + 1141 |
| 3036 | BL21(DE3) | pRSFDuet1 *cspA* + pET22b GFP-CPD-His_6_ |  | This study | 3028 + 196 |
| 3182 | BL21(DE3) | pRSFDuet1 *cspB* + pET22b *cspA*-CPD-His_6_ |  | This study | 3026 + 3125 |
| 3183 | BL21(DE3) | pRSFDuet1 *cspB* + pET22b *cspB*-CPD-His_6_ |  | This study | 3026 + 3175 |
| 3184 | BL21(DE3) | pRSFDuet1 *cspB* + pET22b *cspC*-CPD-His_6_ |  | This study | 3026 + 1141 |
| 3185 | BL21(DE3) | pRSFDuet1 *cspB* + pET22b GFP-CPD-His_6_ |  | This study | 3026 + 196 |
| 3186 | BL21(DE3) | pRSFDuet1 *cspC* + pET22b *cspA*-CPD-His_6_ |  | This study | 3177 + 3125 |
| 3187 | BL21(DE3) | pRSFDuet1 *cspC* + pET22b *cspB*-CPD-His_6_ |  | This study | 3177 + 3175 |
| 3188 | BL21(DE3) | pRSFDuet1 *cspC* + pET22b *cspC*-CPD-His_6_ |  | This study | 3177 + 1141 |
| 3189 | BL21(DE3) | pRSFDuet1 *cspC* + pET22b GFP-CPD-His_6_ |  | This study | 3177 +196 |
| 3032 | DH5α | pRSFDuet1 *cspB*(SR)-*cspA*(QS) (separated at YabG cleavage site) |  | This study | <https://benchling.com/s/seq-W8wbl1eK0QkJG36CDfuR> |
| 3038 | BL21(DE3) | pRSFDuet1 *cspB*(SR)-*cspA*(QS) + pET22b GFP-CPD-His_6_ |  | This study | 3032 + 196 |
| 1141 | DH5α | pET22b *cspC*-CPD-His_6_ (SacI cleavage site) |  | This study | <https://benchling.com/s/seq-TdjM67QsknU2aE5WYNNe?m=slm-AY9PlChV39vERbWksXGG> |
| 3039 | BL21(DE3) | pRSFDuet1 *cspB*(SR)-*cspA*(QS) + pET22b *cspC*-CPD-His_6_ |  | This study | 3032 + 1141 |
| 1375 | DH5α | pET28a *cspBA* |  | This study | <https://benchling.com/s/seq-MAgRIBMVnTJiLTiJLWVw?m=slm-ol2mAeJ3QrwT5yReiMVw> |
| 3062 | BL21(DE3) | pET28a *cspBA* + pET22b *cspC*-CPD-His_6_ |  | This study | 1375 + 1141 |
| 3330 | BL21(DE3) | pRSFDuet1 cspA (*F363E/Y511E*) + pET22b *cspC*-CPD-His_6_ | *cspBA (F944E/ Y1092E)* | This study | 1150 + 3322 |
| 4498 | DH5α | pET22b *cspC* (*D429A/R455A*) -CPD-His_6_ |  | This study | <https://benchling.com/s/seq-KEwmAg4VOLYyuisldFHd?m=slm-tLY8xaXWrIvHrIgC4n7x> |
| 4501 | BL21(DE3) | pRSFDuet1 *cspA* (*F363E/Y511E*) + pET22b *cspC* (*D429A/R456A*) -CPD-His_6_ | *cspBA (F944E/ Y1092E)* | This study | 3028 + 4498 |
| 4497 | DH5α | pRSFDuet1 *cspA* (*D427A/R455A*) | *cspBA (D1008A/R1036A)* | This study | <https://benchling.com/s/seq-BxuGLD0v8CWOgUSc5zBS?m=slm-6x0YWAHbS5WbBjY6dfPM> |
| 4500 | BL21(DE3) | pRSFDuet1 *cspA* (*D427A/R455A*) + pET22b *cspC*-CPD-His_6_ | *cspBA (D1008A/R1036A)* | This study | 4497 + 982 |
| 3956 | DH5α | pET22b *cspC* (*Q516A/T520A*) -CPD-His_6_ |  | This study | <https://benchling.com/s/seq-51gSajur6KosTvCzIhiP?m=slm-hO2HE0KPKY1Jut06HmRd> |
| 3959 | BL21(DE3) | pRSFDuet1 *cspA* + pET22b *cspC* (*Q516A/T520A*) -CPD-His_6_ |  | This study | 3956 + 3028 |
| 3441 | DH5α | pRSFDuet1 *cspA* (*R315A*) | *cspBA (R896A)* | This study | <https://benchling.com/s/seq-AQ7B38uOVMysgkj70cpu?m=slm-wpqSxEUwWEP80RZL3oVF> |
| 4263 | DH5α | pET22b *cspC* (*Q516E/T520E*) -CPD-His_6_ |  | This study | <https://benchling.com/s/seq-wcyDqcuawxqcFQzdDwGl?m=slm-2Vz1jUYWvGQwJjb5H6UQ> |
| 4267 | BL21(DE3) | pRSFDuet1 *cspA* + pET22b *cspC* (*Q516E/T520E*) -CPD-His_6_ |  | This study | 3028 + 4263 |
| 3443 | BL21(DE3) | pRSFDuet1 *cspA* (*R315A*) + pET22b cspC-CPD-His_6_ | *cspBA (R896A)* | This study | 3441 + 982 |
| 4355 | DH5α | pRSFDuet1 *cspA* (*R315E*) | *cspBA (R896E)* | This study | <https://benchling.com/s/seq-YnSg1x5pTs67fb2aN4Pj?m=slm-sMZUxgqHDwvu5lBO6czv> |
| 4365 | BL21(DE3) | pRSFDuet1 *cspA* (*R315E*) + pET22b *cspC*-CPD-His_6_ | *cspBA (R896E)* | This study | 4355 + 982 |
| 3771 | DH5α | pRSFDuet1 *cspA* (*Q509A*) | *cspBA (Q1090A)* | This study | <https://benchling.com/s/seq-gRcZ1E7m0MkNDgyJBXLF?m=slm-yI4A2MFzgkCC3teHecYj> |
| 3776 | BL21(DE3) | pRSFDuet1 *cspA* (*Q509A*) + pET22b *cspC*-CPD-His_6_ | *cspBA (Q1090A)* | This study | 3771 + 982 |
| 3962 | BL21(DE3) | pRSFDuet1 *cspA* (*R315A*) + pET22b *cspC* (*Q516A/T520A*) -CPD-His_6_ | *cspBA (R896A)* | This study | 3441 + 3956 |
| 4429 | BL21(DE3) | pRSFDuet1 *cspA* (*R315E*) + pET22b *cspC* (*Q516E/T520E*) -CPD-His_6_ | *cspBA (R896E)* | This study | 4355 + 4263 |
| 3420 | BL21(DE3) | pET22b *cspA* (*R315A*) -His_6_ | *cspBA (R896A)* | This study | <https://benchling.com/s/seq-jsyOg1jK7jGyg5sQHo0I?m=slm-7IoypHx3fOC6YFKsIPe3> |
| 4352 | BL21(DE3) | pET22b *cspA* (*R315E*) -His_6_ | *cspBA (R896E)* | This study | <https://benchling.com/s/seq-RuiIOpXG0UWlZTB69ZNs?m=slm-zZ3NBsGDayHOlzd232yG> |
| 4266 | BL21(DE3) | pET22b *cspA* (*Q513A/T517A*) -His_6_ | *cspBA (Q1094A/T1098A)* | This study | <https://benchling.com/s/seq-raQi6SZWCxyaVgeXT4La?m=slm-3pVMnJNoSwwGGPHRciwY> |
| 3738 | BL21(DE3) | pET22b *cspA* (*R315E/Q513E*) -His_6_ | *cspBA (R896E/Q1094E)* | This study | <https://benchling.com/s/seq-Ih6mswO7BivuNGcnviwm?m=slm-82TejOkXZDbyLlyi0RpC> |
| 3739 | BL21(DE3) | pET22b *cspA* (*R315A/Q513A/T517A*) -His_6_ | *cspBA (R896A/ Q1094A/ T1098A)* | This study | <https://benchling.com/s/seq-Brz71yP6632s3EGZw6dJ?m=slm-BjK8uOsww5MYRjNyzc5T> |
| 4497 | DH5α | pET22b *cspA* (*D427A-R455A*) -His_6_ | *cspBA (D1008A/R1036A)* | This study | <https://benchling.com/s/seq-BxuGLD0v8CWOgUSc5zBS?m=slm-6x0YWAHbS5WbBjY6dfPM> |
| 4607 | BL21(DE3) | pRSFDuet1 cspA (*D427A/R455A*) + pET22b cspC (Q516E/T520E-CPD-His6 | *cspBA (D1008A/R1036A)* | This study | 4497 + 4263 |
| 4276 | DH5α | pET22b *cspA* (*Q513E/T517E*)-His_6_ | *cspBA (Q1094E/T1098E)* | This study | <https://benchling.com/s/seq-hEJelfZrRoELjQ38hQ5I?m=slm-6mK6qwxM9gZBeHGo1NIc> |
| 4279 | BL21(DE3) | pRSFDuet1 *cspA* (*Q513E/T517E*) + pET22b *cspC*-CPD-His_6_ | *cspBA (Q1094E/T1098E)* | This study | 4276 + 982 |
| 3775 | DH5α | pRSFDuet1 *cspA* (*R315A/Q513A/T517A*) | *cspBA (R896A/ Q1094A/ T1098A)* | This study | <https://benchling.com/s/seq-HXk9UtTKw2JWCWaHvH03?m=slm-TOARN4mFSALsNAxQstnT> |
| 3780 | BL21(DE3) | pRSFDuet1 *cspA* (*R315A/Q513A/T517A*) + pET22b *cspC*-CPD-His_6_ | *cspBA (R896A/ Q1094A/ T1098A)* | This study | 3775 + 982 |
| 3773 | DH5α | pRSFDuet1 *cspA* (*R315E/Q513E*) | *cspBA (R896E/ Q1094E)* | This study | <https://benchling.com/s/seq-uQgmmCN2f7Yh3i7adqL7?m=slm-0NzdgOzjURSyRt1O5Mf9> |
| 3778 | BL21(DE3) | pRSFDuet1 *cspA* (*R315E/Q513E*) + pET22b *cspC*-CPD-His_6_ | *cspBA (R896E/ Q1094E)* | This study | 3773 + 982 |
| 2319 | HB101 | pMTL-YN1C ∆*cspBA*-*cspC* (*D429A*) |  | This study | <https://benchling.com/s/seq-z2G3ruS2lvcH80u1Rwvw?m=slm-kowGZjHPxwbhqKGYdJfl> |
| 2323 | HB101 | pMTL-YN1C ∆*cspBA*-*cspC* (*R456A*) |  | This study | <https://benchling.com/s/seq-p0Xk1hs7ycryfKYg448A?m=slm-M2VGBcvRvh1nRP6wpYZM> |
| 3415 | HB101 | pMTL-YN1C *cspBA* (*F944E/Y1092E*) -*∆cspC* |  | This study | <https://benchling.com/s/seq-SGhWAlBC6TCiMRS9FSFF?m=slm-RYkChzCvXZMp9dNE5rye> |
| 3464 | HB101 | pMTL-YN1C *cspBA* (*R896A*) -*∆cspC* |  | This study | <https://benchling.com/s/seq-0Am25X49EgDC56tglBH2?m=slm-9DqffC4yzxDy08mB1HQd> |
| 3631 | HB101 | pMTL-YN1C *cspBA* (*Q1094A*) -*∆cspC* |  | This study | <https://benchling.com/s/seq-jmABTGrcShpWPwyJ4HdS?m=slm-gDmjN3Vr5cOo8btXFOWm> |
| 3756 | HB101 | pMTL-YN1C *cspBA* (*R896E*/*Q1094E*) -*∆cspC* |  | This study | <https://benchling.com/s/seq-EWvyHHMB45ge4gZiPBba?m=slm-482nJo5t9NHbdpJkY7L0> |
| 3763 | HB101 | pMTL-YN1C *cspBA* (*R896A/Q1094A/T1098A*) -*∆cspC* |  | This study | <https://benchling.com/s/seq-VukOqyJsJTOEOLuRXb7e?m=slm-LeDo42Le6kICuWmG34Cy> |
| 3948 | HB101 | pMTL-YN1C ∆*cspBA*-*cspC* (*T520A*) |  | This study | <https://benchling.com/s/seq-XgCD8RKy40cjXHqSzzTt?m=slm-Hp3jde5y6gsUjjjrpwg8> |
| 3950 | HB101 | pMTL-YN1C ∆*cspBA*-*cspC* (Q516A/*T520A*) |  | This study | <https://benchling.com/s/seq-N7pUIsnVL1299FjC7dvx?m=slm-Ij8xrpfJT4AtpiWeVREw> |
| 3977 | HB101 | pMTL-YN1C ∆*cspBA*-*cspC* (*Q516A*) |  | This study | <https://benchling.com/s/seq-LVL7jxhC22osyzLDwZAd?m=slm-WIKaBO0OwxFuzIs62dSA> |
| 4025 | HB101 | pMTL-YN1C *cspBA* (*R1036A*) -*∆cspC* |  | This study | <https://benchling.com/s/seq-dDd98vjXQFPE4jaPycbp?m=slm-NnvDgiR9wEJ7JoLkqa4r> |
| 4140 | HB101 | pMTL-YN1C *cspBA* (*R896A*) *cspC* (*Q516A/T520A*) |  | This study | <https://benchling.com/s/seq-bQLdxDH6lXVe2qTRUwbG?m=slm-o8T5kVcwNytoCNX2cydc> |
| 4246 | HB101 | pMTL-YN1C *cspBA* (*D1008K*) -∆*cspC* |  | This study | <https://benchling.com/s/seq-lYGerWVu2UN2BiTXueD0?m=slm-bigJ3kschjFCEt0aygD3> |
| 4270 | HB101 | pMTL-YN1C *∆cspBA*-*cspC* (*Q516E/T520E*) |  | This study | <https://benchling.com/s/seq-oujgMjwxRnx4ovFlugsB?m=slm-Hz4o9U6iLax1w9g9IqDw> |
| 4327 | HB101 | pMTL-YN1C *cspBA* (*R896E*) -*∆cspC* |  | This study | <https://benchling.com/s/seq-B4bRcv0YCy2UsvpLN3CD?m=slm-0hWXU9YwxlTgENlI5rQp> |
| 4329 | HB101 | pMTL-YN1C *cspBA* (*Q1094E/T1098E*) -*∆cspC* |  | This study | <https://benchling.com/s/seq-0WqJsTmQrmcox4b59Apo?m=slm-7haJImSdyVbxwXTkTEuX> |
| 4435 | HB101 | pMTL-YN1C *cspBA* (*R896E*) -*cspC* (*Q516E/T520E*) |  | This study | <https://benchling.com/s/seq-KtkadpfKaFdzIwk8chZx?m=slm-QErY1Fj9FTljEOKXwcWT> |
| 4437 | HB101 | pMTL-YN1C *cspBA* (*D1008A*) -*∆cspC* |  | This study | <https://benchling.com/s/seq-YE0Y9yIp8iNHCDNOIdfC?m=slm-8mxVWB7kK5tZhq2mXQvd> |
| 4450 | HB101 | pMTL-YN1C *cspBA* (*D1008A/R1036A*) -*∆cspC* |  | This study | <https://benchling.com/s/seq-WJWSGgtW3WqZrYc7eu5n?m=slm-Z0hpFI4VHcK56fc6Zhn2> |
| 4496 | HB101 | pMTL-YN1C ∆*cspBA*-*cspC* (*D429A/R456A*) |  | This study | <https://benchling.com/s/seq-Rcy1o5FpLAv5upkampYz?m=slm-mL2904knLST8hTYS81kg> |
| 4503 | HB101 | pMTL-YN1C *cspBA* (*D1008A/R1036A*) -cspC (*Q516E/T520E*) |  | This study | <https://benchling.com/s/seq-UnANzT4jJE6RBwhKM9U0?m=slm-WMaGOMYG5bC40OGTqIga> |

Amino acid numbering based on YabG-cleaved CspA. “*cspBA* numbering” based on *cspBA* fusion gene. All *C. difficile* genes expressed in *E. coli* were designed with codon optimization for *E. coli* expression.

**References**

1. Rohlfing AE, Eckenroth BE, Forster ER, Kevorkian Y, Donnelly ML, Benito de la Puebla H, et al. The CspC pseudoprotease regulates germination of *Clostridioides difficile* spores in response to multiple environmental signals. PLoS Genet. 2019;15(7):e1008224. Epub 2019/07/06. doi: 10.1371/journal.pgen.1008224. PubMed PMID: 31276487; PubMed Central PMCID: PMCPMC6636752 following competing interests: AS is a paid consultant of BioVector, a start-up company focused on diagnostics.

2. Shen A, Lupardus PJ, Morell M, Ponder EL, Sadaghiani AM, Garcia KC, et al. Simplified, enhanced protein purification using an inducible, autoprocessing enzyme tag. PLoS One. 2009;4(12):e8119. Epub 2009/12/04. doi: 10.1371/journal.pone.0008119. PubMed PMID: 19956581; PubMed Central PMCID: PMCPMC2780291.
