# Supplementary material for: The CspC:CspA heterodimer transduces germinant and co-germinant signals during Clostridioides difficile spore germination
Source: PLoS Biol. 2026 Feb 2;24(2):e3003610. doi: 10.1371/journal.pbio.3003610 (PMC12880746; doi:10.1371/journal.pbio.3003610)
Supplement: S4 Table — cspBA numbering based on cspBA fusion gene. (DOCX) [file pbio.3003610.s004.docx]

**Supplemental Table 4. *C. difficile* strains used in this study.**

| Lab Strain # | Strain name | Relevant genotype | Source/Reference |
| --- | --- | --- | --- |
| 789 | 630*∆erm∆pyrE∆cspBA* | *630∆erm∆pyrE* with *cspBA* deleted | [1] |
| 799 | 630*∆erm∆pyrE∆cspC* | *630∆erm∆pyrE* with *cspC* deleted | [1] |
| 831 | 630*∆erm∆pyrE∆cspC/cspC* | *630∆erm∆cspC* with *cspC* in the *pyrE* locus | [1] |
| 846 | 630*∆erm*-P | *erm*-sensitive derivative of 630 with *pyrE* restored | [1] |
| 859 | 630*∆erm∆cspBA*-P | *630∆erm∆cspBA* with *pyrE* restored | [1] |
| 928 | 630*∆erm∆pyrE∆cspBAC* | *630∆erm∆pyrE* with *cspBAC* deleted | [1] |
| 1150 | 630*∆erm∆cspBAC*-P | *630∆erm∆cspBAC* with *pyrE* restored | [1] |
| 1153 | 630*∆erm∆pyrE∆cspBAC/cspBAC* | *630∆erm∆cspBAC* with *cspBAC* in the *pyrE* locus | [2] |
| 1205 | 630*∆erm∆pyrE∆cspBA/cspBA* | *630∆erm∆cspBA* with *cspBA* in the *pyrE* locus | [1] |
| 1239 | 630*∆erm∆cspC*-P | *630∆erm∆cspC* with *pyrE* restored | [1] |
| 1920 | 630*∆erm∆pyrE∆cspC/cspC (D429K)* | *630∆erm∆cspC* with *cspC (D429K)* in the *pyrE* locus | [3] |
| 4168 | 630*∆erm∆pyrE∆cspBA/cspBA (F944E/Y1092E)* | *630∆erm∆cspBA* with *cspBA (F944E/Y1092E)* in the *pyrE* locus | This study |
| 4241 | 630*∆erm∆pyrE∆cspBA/cspBA (R896A)* | *630∆erm∆cspBA* with *cspBA (R896A)* in the *pyrE* locus | This study |
| 4377 | 630*∆erm∆pyrE∆cspBA/cspBA (Q1090A)* | *630∆erm∆cspBA* with *cspBA (R896A)* in the *pyrE* locus | This study |
| 4499 | 630*∆erm∆pyrE∆cspBA/cspBA (R896E/Q1094E)* | *630∆erm∆cspBA* with *cspBA (R896E/Q1094E)* in the *pyrE* locus | This study |
| 4617 | 630*∆erm∆pyrE∆cspBA/cspBA (R896A/Q1094A/T1098A)* | *630∆erm∆cspBA* with *cspBA (R896A/Q1094A/T1098A)* in the *pyrE* locus | This study |
| 4847 | 630*∆erm∆pyrE∆cspC/cspC (T520A)* | *630∆erm∆cspC* with *cspC (T520A)* in the *pyrE* locus | This study |
| 4850 | 630*∆erm∆pyrE∆cspC/cspC (Q516A/T520A)* | *630∆erm∆cspC* with *cspC (Q516A/T520A)* in the *pyrE* locus | This study |
| 4877 | 630*∆erm∆pyrE∆cspC/cspC (Q516A)* | *630∆erm∆cspC* with *cspC (Q516A)* in the *pyrE* locus | This study |
| 4880 | 630*∆erm∆pyrE∆cspBA/cspBA (R1036A)* | *630∆erm∆cspBA* with *cspBA (R1036A)* in the *pyrE* locus | This study |
| 4975 | 630*∆erm∆pyrE∆cspBAC/cspBA (R896A) cspC (Q516A/T520A)* | *630∆erm∆cspBAC* with *cspBA (R896A)* *cspC (Q516A/T520A)* in the *pyrE* locus | This study |
| 5077 | 630*∆erm∆pyrE∆cspBA/cspBA (D1008K)* | *630∆erm∆cspBA* with *cspBA (D1008K)* in the *pyrE* locus | This study |
| 5091 | 630*∆erm∆pyrE∆cspC/cspC (Q516E/T520E)* | *630∆erm∆cspC* with *cspC (Q516E/T520E)* in the *pyrE* locus | This study |
| 5147 | 630*∆erm∆pyrE∆cspBA/cspBA (R896E)* | *630∆erm∆cspBA* with *cspBA (R896E)* in the *pyrE* locus | This study |
| 5150 | 630*∆erm∆pyrE∆cspBA/cspBA (Q1094E/T1098E)* | *630∆erm∆cspBA* with *cspBA (Q1094E/T1098E)* in the *pyrE* locus | This study |
| 5276 | 630*∆erm∆pyrE∆cspC/cspC (D429A)* | *630∆erm∆cspC* with *cspC (D429A)* in the *pyrE* locus | This study |
| 5278 | 630*∆erm∆pyrE∆cspC/cspC (R456A)* | *630∆erm∆cspC* with *cspC (R456A)* in the *pyrE* locus | This study |
| 5287 | 630*∆erm∆pyrE∆cspBAC/cspBA (R896E) cspC (Q516E/T520E)* | *630∆erm∆cspBAC* with *cspBA (R896E)* *cspC (Q516E/T520E)* in the *pyrE* locus | This study |
| 5328 | 630*∆erm∆pyrE∆cspBA/cspBA (D1008A)* | *630∆erm∆cspBA* with *cspBA (D1008A)* in the *pyrE* locus | This study |
| 5330 | 630*∆erm∆pyrE∆cspBA/cspBA (D1008A/R1036A)* | *630∆erm∆cspBA* with *cspBA (D1008A/R1036A)* in the *pyrE* locus | This study |
| 5451 | 630*∆erm∆pyrE∆cspC/cspC (D429A/R456A)* | *630∆erm∆cspC* with *cspC (D429A/R456A)* in the *pyrE* locus | This study |
| 5455 | 630*∆erm∆pyrE∆cspBAC/cspBA (D1008A/R1036A) cspC (Q516E/T520E)* | *630∆erm∆cspBAC* with *cspBA (D1008A/R1036A)* *cspC (Q516E/T520E)* in the *pyrE* locus | This study |

*cspBA* numbering based on *cspBA* fusion gene.

**References**

1. Kevorkian Y, Shen A. Revisiting the Role of Csp Family Proteins in Regulating Clostridium difficile Spore Germination. J Bacteriol. 2017;199(22). Epub 2017/09/07. doi: 10.1128/JB.00266-17. PubMed PMID: 28874406; PubMed Central PMCID: PMCPMC5648855.

2. Donnelly ML, Li W, Li YQ, Hinkel L, Setlow P, Shen A. A *Clostridium difficile*-Specific, Gel-Forming Protein Required for Optimal Spore Germination. mBio. 2017;8(1). Epub 2017/01/18. doi: 10.1128/mBio.02085-16. PubMed PMID: 28096487; PubMed Central PMCID: PMCPMC5241399.

3. Rohlfing AE, Eckenroth BE, Forster ER, Kevorkian Y, Donnelly ML, Benito de la Puebla H, et al. The CspC pseudoprotease regulates germination of *Clostridioides difficile* spores in response to multiple environmental signals. PLoS Genet. 2019;15(7):e1008224. Epub 2019/07/06. doi: 10.1371/journal.pgen.1008224. PubMed PMID: 31276487; PubMed Central PMCID: PMCPMC6636752 following competing interests: AS is a paid consultant of BioVector, a start-up company focused on diagnostics.
