# Supplementary material for: The CspC:CspA heterodimer transduces germinant and co-germinant signals during Clostridioides difficile spore germination
Source: PLoS Biol. 2026 Feb 2;24(2):e3003610. doi: 10.1371/journal.pbio.3003610 (PMC12880746; doi:10.1371/journal.pbio.3003610)
Supplement: S5 Table — Strain name and clade number (1–5) for C. difficile strains [37,38] from Lewis and colleagues [37] and Knight and colleagues [38] used for sequence alignment of cspC and cspA genes. (DOCX) [file pbio.3003610.s005.docx]

**Supplemental Table 5. *C. difficile* clinical isolates used for *cspC* and *cspA* sequence alignment.**

| **Strain** | **Clade** | **Reference** |
| --- | --- | --- |
| CD306 | 3 | [1] |
| TL178 | 3 | [1] |
| 112C | 1 | [2] |
| 139B | 5 | [2] |
| 167A | 1 | [2] |
| 179G | 1 | [2] |
| 190B | 1 | [2] |
| 205A | 1 | [2] |
| 217B | 1 | [2] |
| 503A | 1 | [2] |
| 557A | 1 | [2] |
| 610B | 1 | [2] |
| 615H | 1 | [2] |
| 617F | 1 | [2] |
| 678B | 1 | [2] |
| 733B | 1 | [2] |
| 1002 | 4 | [2] |
| BBL1 | 1 | [2] |
| BBL2 | 1 | [2] |
| BBL3 | 2 | [2] |
| BBL4 | 2 | [2] |
| HV115 | 1 | [2] |
| WU13 | 1 | [2] |
| WU14 | 2 | [2] |
| WU38 | 1 | [2] |
| WU42 | 1 | [2] |
| WU66 | 5 | [2] |
| WU82 | 1 | [2] |
| WUp4 | 2 | [2] |
| WUp8 | 2 | [2] |
| WUp9 | 1 | [2] |
| WUp19 | 1 | [2] |
| WUp26 | 1 | [2] |
| WUp43 | 1 | [2] |

**References**

1. Knight DR, Imwattana K, Kullin B, Guerrero-Araya E, Paredes-Sabja D, Didelot X, et al. Major genetic discontinuity and novel toxigenic species in *Clostridioides difficile* taxonomy. eLife. 2021;10. Epub 20210611. doi: 10.7554/eLife.64325. PubMed PMID: 34114561; PubMed Central PMCID: PMCPMC8241443.

2. Lewis BB, Carter RA, Ling L, Leiner I, Taur Y, Kamboj M, et al. Pathogenicity Locus, Core Genome, and Accessory Gene Contributions to *Clostridium difficile* Virulence. mBio. 2017;8(4). Epub 20170808. doi: 10.1128/mBio.00885-17. PubMed PMID: 28790208; PubMed Central PMCID: PMCPMC5550754.
