# Supplementary figures and images for: The CspC:CspA heterodimer transduces germinant and co-germinant signals during Clostridioides difficile spore germination
Source: PLoS Biol. 2026 Feb 2;24(2):e3003610. doi: 10.1371/journal.pbio.3003610 (PMC12880746; doi:10.1371/journal.pbio.3003610)

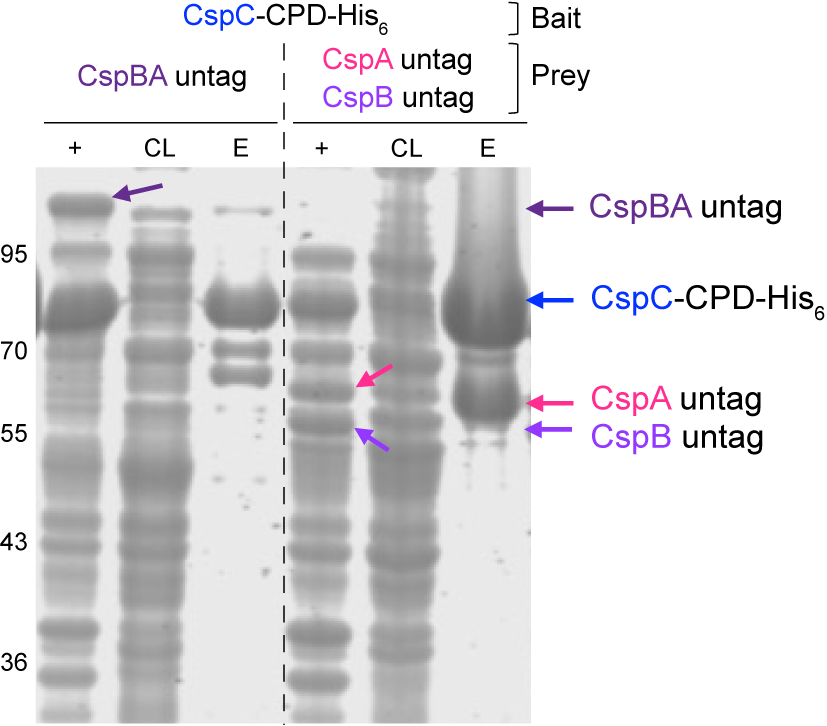

Supplement: S1 Fig — Coomassie stain of co-affinity purifications using CspC-CPD-His6 as the bait and untagged CspBA or CspB and CspA as the prey. +, induced fraction; CL, cleared lysate; E, elution. The data shown are representative of two independent replicates. The data shown are representative of two independent replicates. The raw gel image can be found in S1 Raw Images. (TIF) [file pbio.3003610.s006.tif]

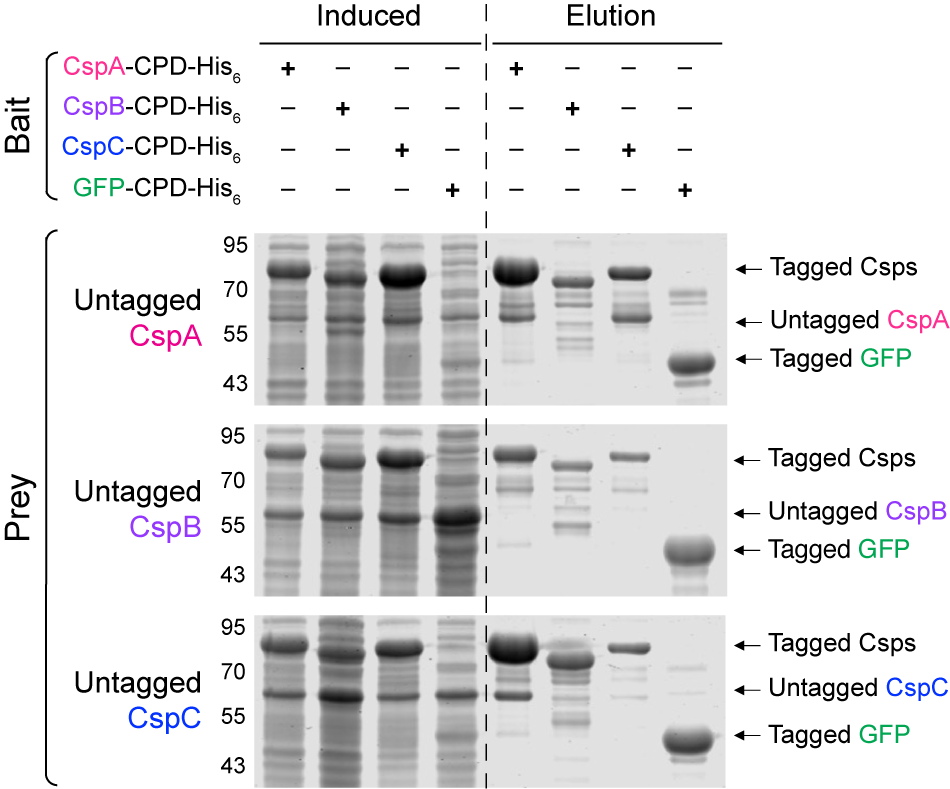

Supplement: S2 Fig — Coomassie stain of co-affinity purification analyses using CPD-His6-tagged CspA, CspB, or CspC as the bait alongside their respective untagged Csps as prey. GFP-CPD-His6 is the control bait. All data shown are representative of three replicates. All data shown are representative of three replicates. The raw gel images can be found in S1 Raw Images. (TIF) [file pbio.3003610.s007.tif]

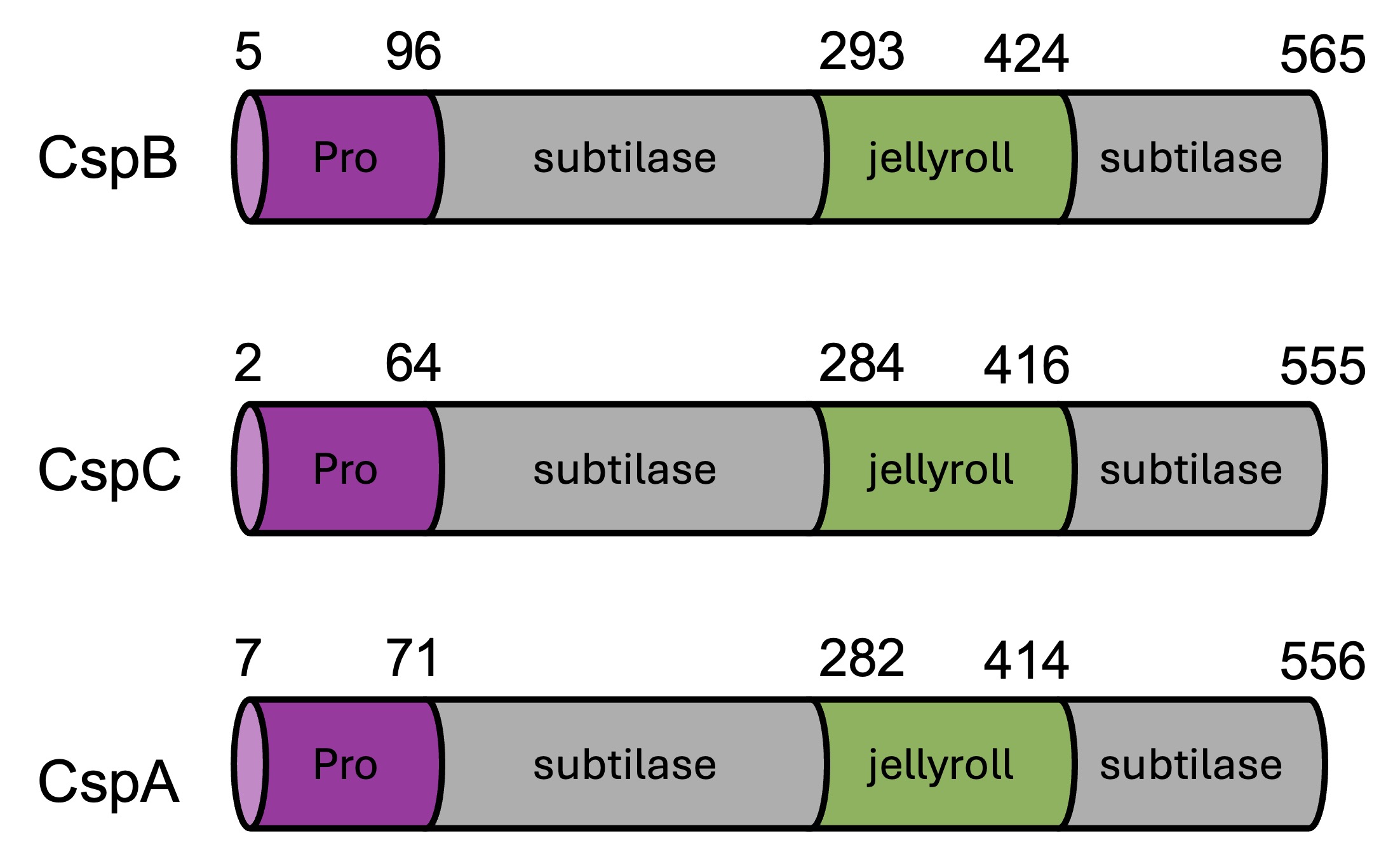

Supplement: S3 Fig — Bounds of the prodomains, subtilase domains, and jellyroll domains of C. perfringens CspB [19] (top), and Clostridioides difficile CspC and CspA (center and bottom, respectively). Bounds are representative of every structure in this work. (JPG) [file pbio.3003610.s008.jpg]

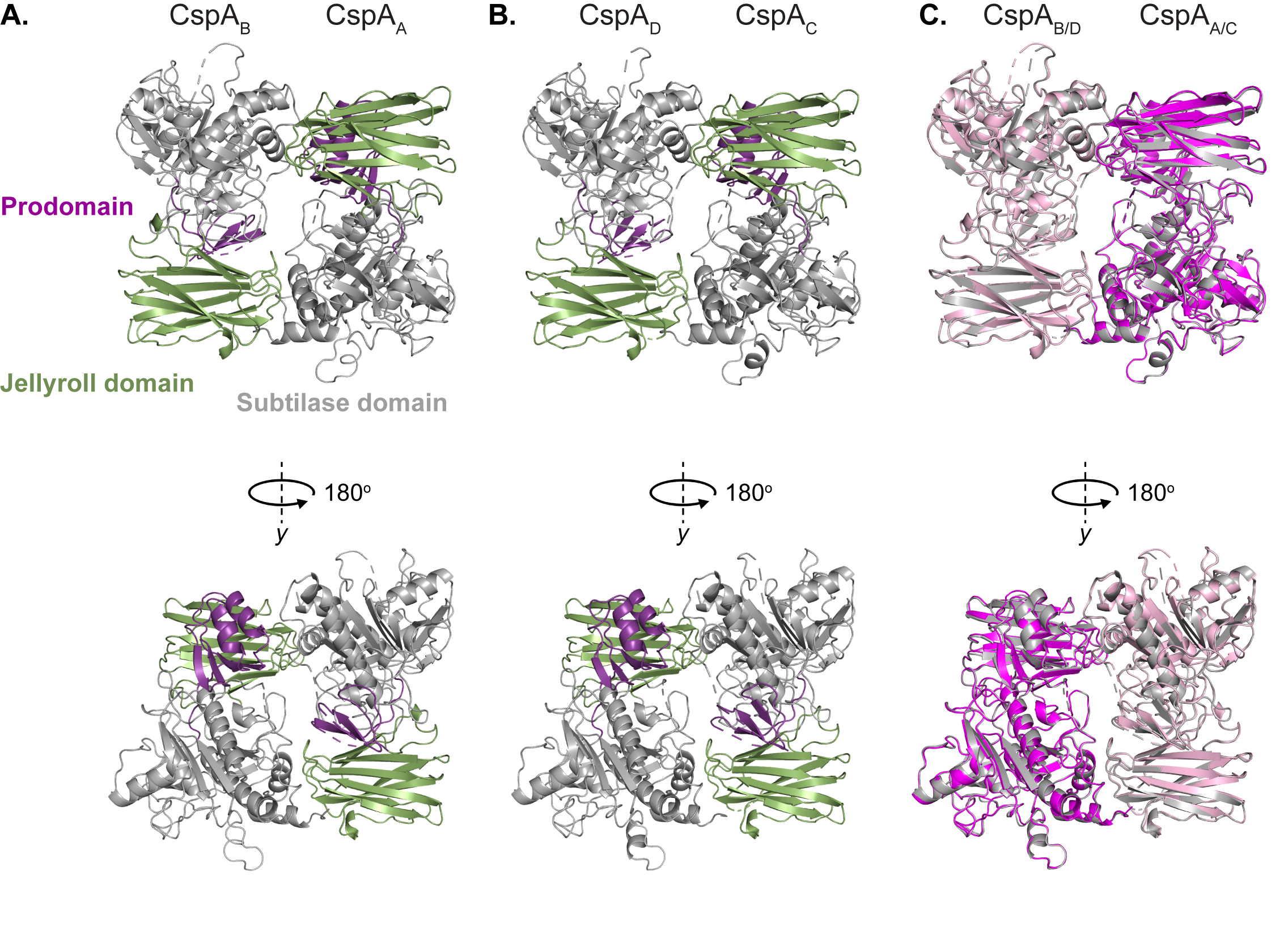

Supplement: S4 Fig — (A, B) The two CspA homodimers that crystallized within the asymmetric unit (PDB 9PR9). The subtilase domains are shown in gray, jellyroll domains in green, and prodomains in purple. The structure used for all other CspA homodimer figures in this manuscript is shown in (A). (C) Overlay of the two CspA homodimer structures (A and B). The A homodimer is shown in light pink (CspAB) and magenta (CspAA), the B homodimer is shown in gray. (TIF) [file pbio.3003610.s009.tif]

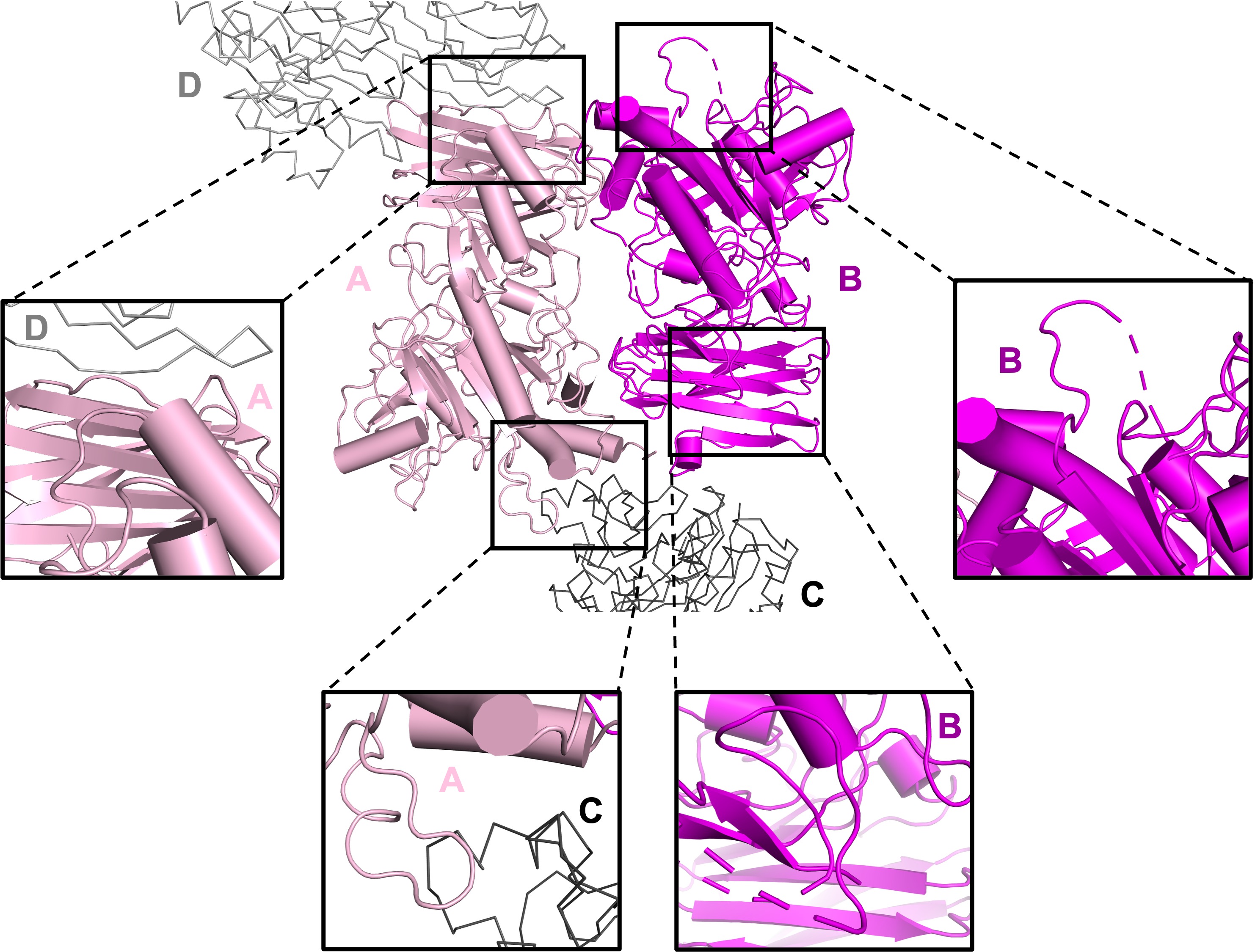

Supplement: S5 Fig — The prodomains of the A and C protomers in the CspA asymmetric unit are better resolved than their counterparts in the B and D protomers. As shown in the insets, the A protomer’s prodomain makes contacts with C and D symmetry mates in the crystal lattice. The N terminus of the A prodomain is stabilized by contacts with the subtilase domain of a C protomer (bottom left inset), while the C terminus of the A prodomain is stabilized by contacts with the D protomer jellyroll domain (top right inset). Because of the screw axis and crystal packing, the B and D protomers lack these contacts with symmetry mates. Only protomers A and B of the asymmetric unit are shown for simplicity. (JPG) [file pbio.3003610.s010.jpg]

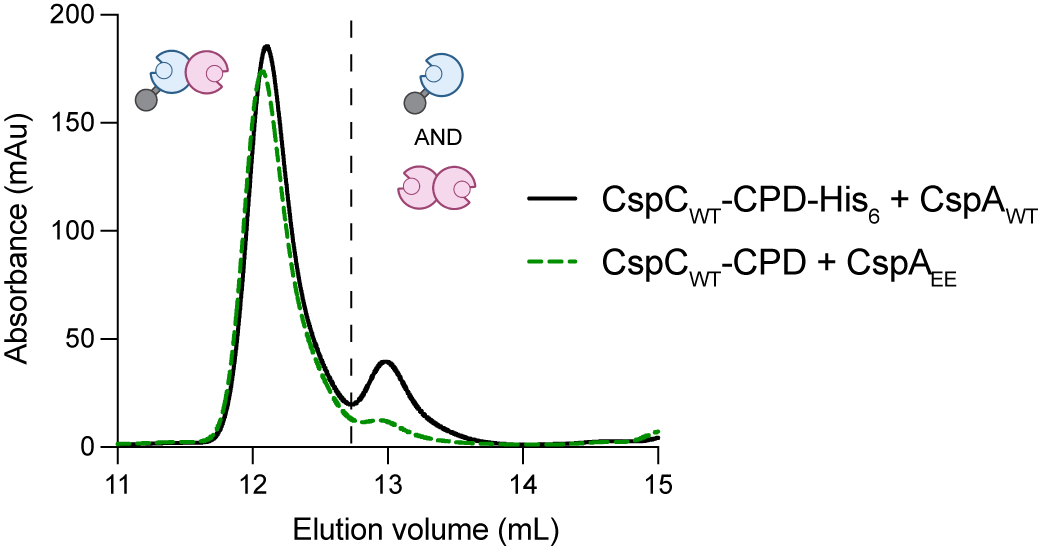

Supplement: S6 Fig — Size exclusion chromatography analysis of CspC-CPD-His6-CspAF944E/Y1092E (CspC:CspAEE) co-affinity purification. The dashed line indicates the separation between the two peaks. The data shown are representative of a minimum of three independent replicates. The data underlying this figure can be found in S1 Data. (TIF) [file pbio.3003610.s011.tif]

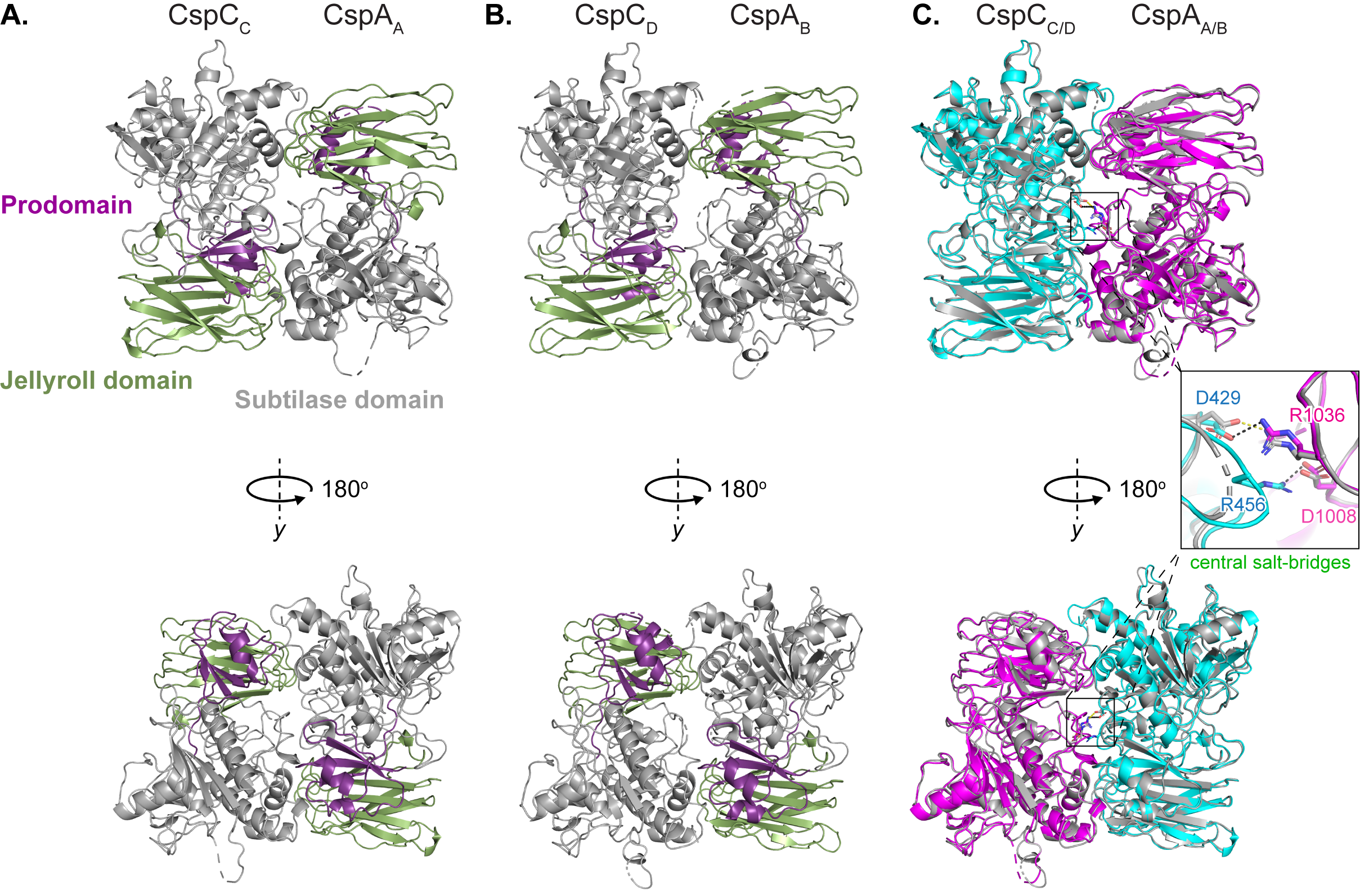

Supplement: S7 Fig — (A, B) The two CspC:CspA heterodimers that crystallized within the asymmetric unit (PDB 9PR8). CspC on the left, CspA on the right. The subtilase domains are shown in gray, jellyroll domains in green, and prodomains in purple. The structure used for all other CspC:CspA heterodimer figures in this manuscript is shown in (A). (C) Overlay of the two CspC:CspA heterodimer structures (A and B). The A heterodimer is shown in cyan (CspCC) and magenta (CspAA); the B heterodimer is shown in gray. (Inset) Two salt bridges between CspC and CspA form within the CspCC:CspAA heterodimer. The CspCC D429:CspAA R1036 salt bridge interaction is a distance of 3.6 Å, and the CspCC R456:CspAA D1008 is 2.9 Å. A single salt-bridge forms between CspCD D429:CspAB R1036 (distance of 3.1 Å). The CspCD R456 residue is unstructured, and no interaction exists between CspCD R456 and CspAB D1008. (TIF) [file pbio.3003610.s012.tif]

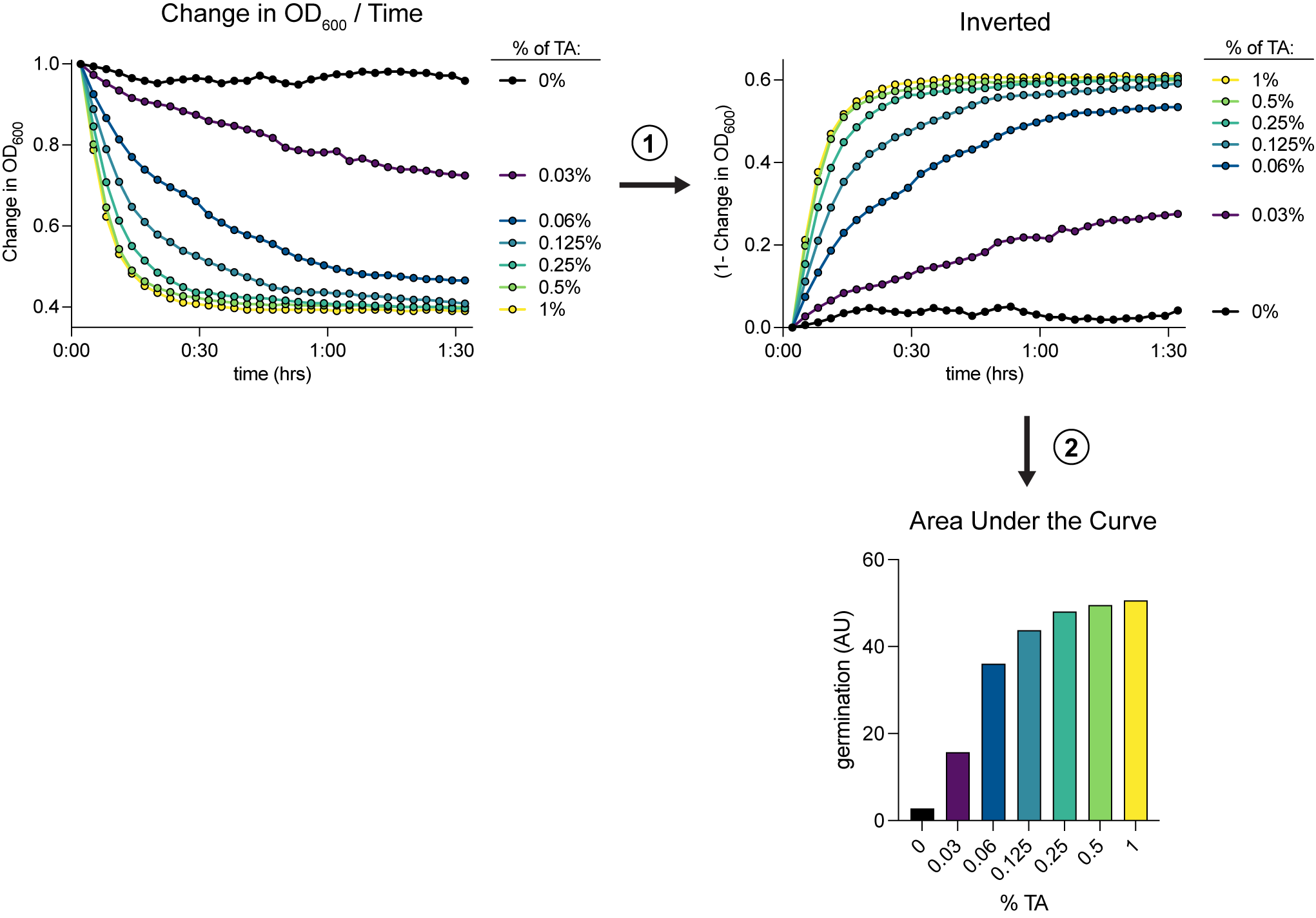

Supplement: S8 Fig — Spore germination was assessed using optical density (OD600). Spores were exposed to germinant and their OD600 was measured at 3-min intervals for 1.5 h. After the OD600 of blank wells (sterile medium) was subtracted, the values were normalized to the first measurements (time 0), and the values were plotted as OD600 versus time (left). (1) The resulting curves were inverted by subtracting each of the values from 1 (center). (2) The area under the inverted curves (center) was plotted for each strain as a bar graph (right). Area under the curve calculations were performed using Prism. All germination assays were performed at the presented concentrations of TA to determine the concentration that best displays a given germinant sensitivity phenotype. The data underlying this figure can be found in S1 Data. (TIF) [file pbio.3003610.s013.tif]

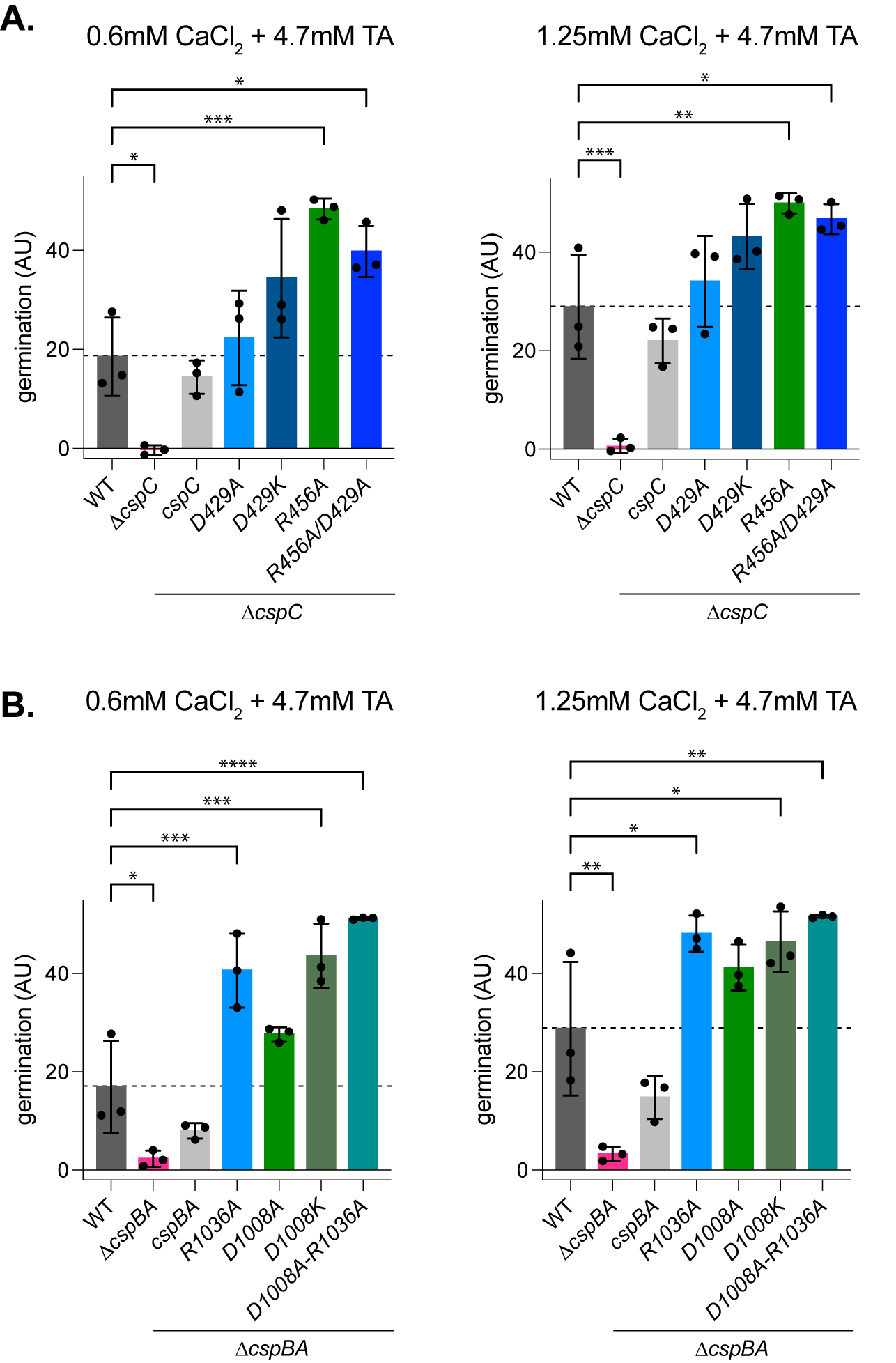

Supplement: S9 Fig — Gemination levels based on the change in optical density (OD600) of purified spores suspended in buffer containing 50 mM HEPES buffer and 100 mM NaCl and the indicated concentration of calcium over time following the addition of 4.7 mM taurocholate (TA). Germination in arbitrary units (AU) was calculated using the area below inverted OD600 curves (S8 Fig). cspC complementation mutants were constructed in a ∆cspC background (A). cspBA complementation mutants were constructed in a ∆cspBA background (B). CspA residue numbers are based on the full-length CspBA fusion protein. Statistical significance relative to WT was determined using a one-way ANOVA and Dunnett’s multiple comparisons test. **** p < 0.0001, *** p < 0.001, ** p < 0.01, * p < 0.1. It should be noted that the specific effect of glycine co-germinant on the germination of the indicated mutants was not assayed due to the complicating effects of internal calcium being released in the form of calcium dipicolinic acid (Ca-DPA). The released calcium can synergize with glycine to promote germination [11]. The data underlying the panels in this figure can be found in S1 Data. (TIF) [file pbio.3003610.s014.tif]

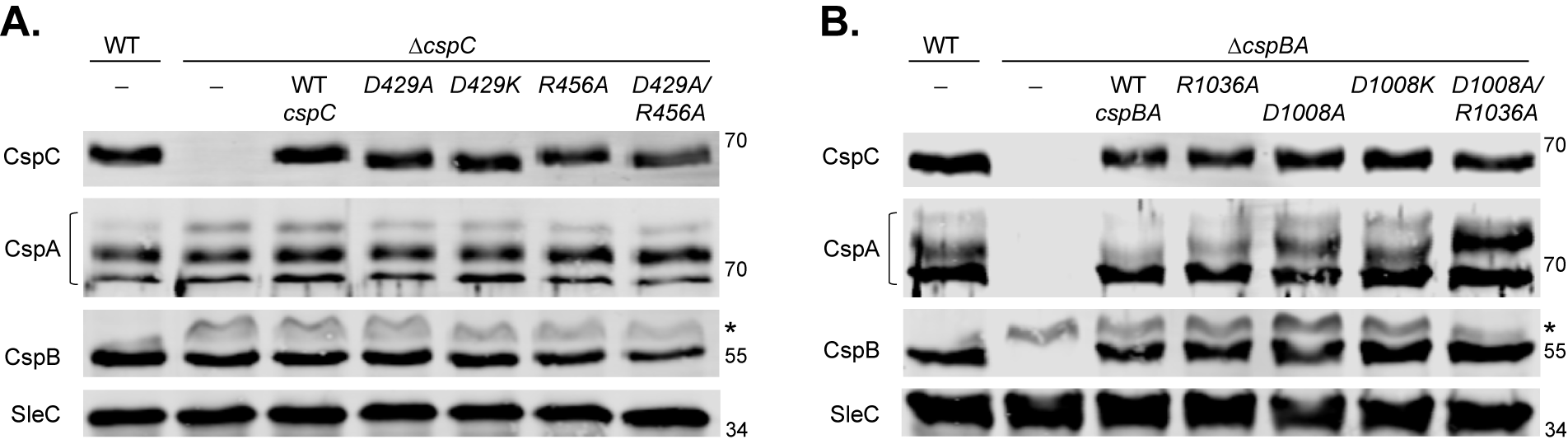

Supplement: S10 Fig — (A, B) western blot analyses of Csp levels in mutant spores. Multiple isoforms of CspA are observed. * indicates a non-specific band. SleC was used as a load control. The data shown are representative of a minimum of three independent replicates. The raw gel images can be found in S1 Raw Images. (TIF) [file pbio.3003610.s015.tif]

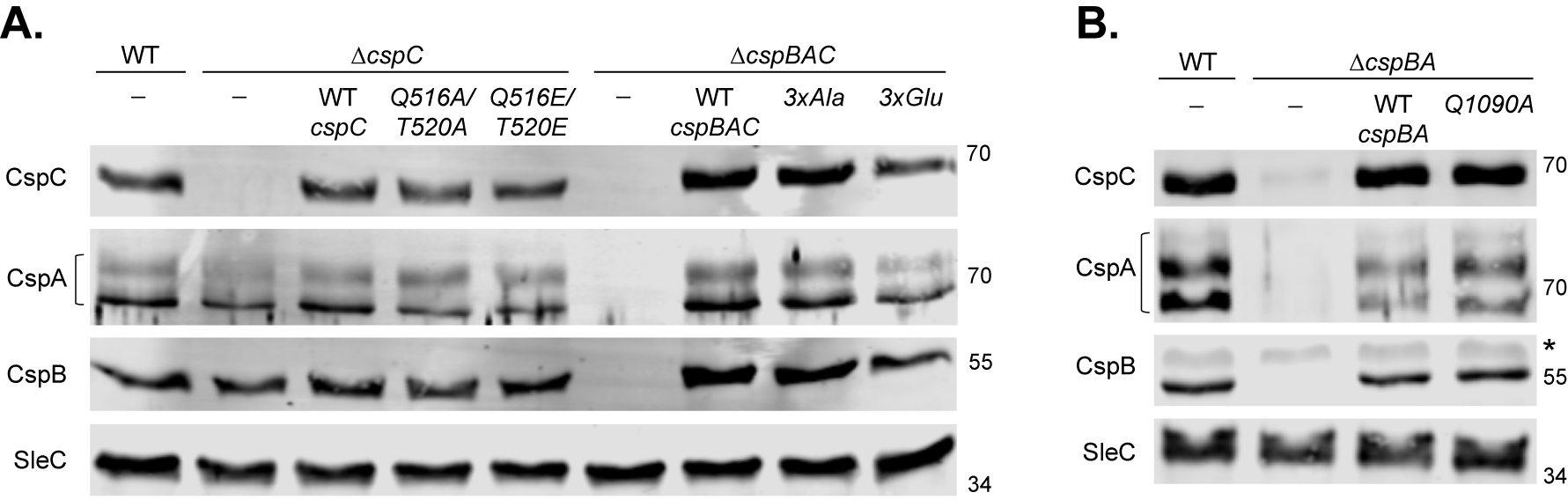

Supplement: S11 Fig — (A, B) western blot analyses of Csp levels in mutant spores. ∆cspBAC/3xAla = triple-Ala substitution cspBAR896A-cspCQ516A/T520A, ∆cspBAC/3xGlu = triple-Glu substitution cspBAR896E-cspCQ516A/T520E. Multiple isoforms of CspA are observed. * indicates a non-specific band. SleC was used as a load control. The data shown are representative of a minimum of three independent replicates. The raw gel images can be found in S1 Raw Images. (TIF) [file pbio.3003610.s016.tif]

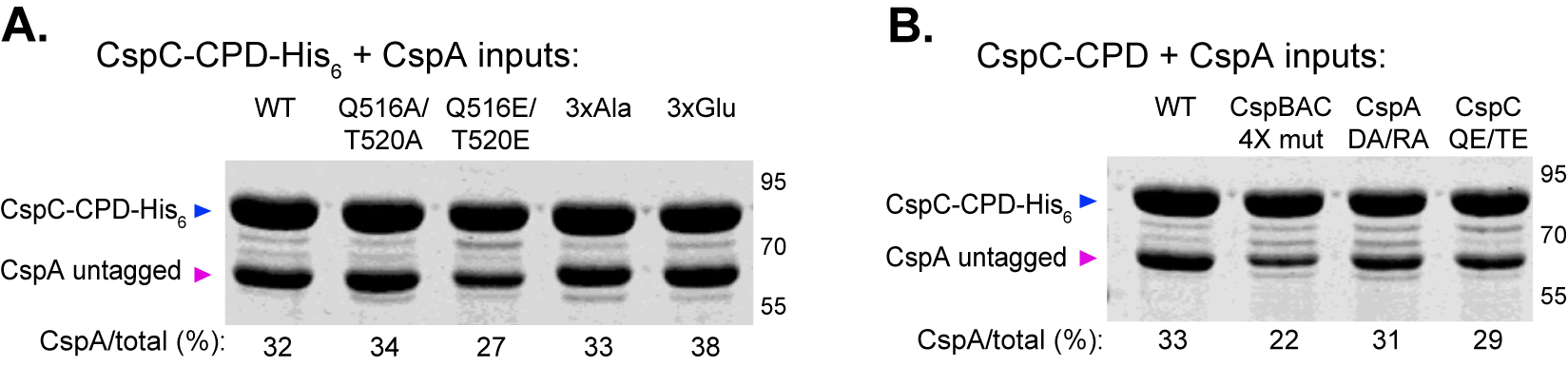

Supplement: S12 Fig — (A, B) SDS-PAGE of CspC-CPD-His6 co-affinity purification inputs for SEC purification, stained with Coomassie. Q516A/T520A represents CspCQ516A/T520A-CPD-His6 with untagged WT CspA; Q516A/T520E represents CspCQ516E/T520E-CPD-His6 with untagged WT CspA; 3xAla represents CspCQ516A/T520A-CPD-His6 with untagged CspAR896A; and 3xGlu represents CspCQ516E/T520E-CPD-His6 with untagged CspAR896E (A). Inputs correspond to SEC traces shown in Fig 4E–4G, 4K, and 4L. (B) CspBAC 4× mut corresponds to CspCQ516E/T520E-CPD-His6 with untagged CspAD1008A/R1036A; CspA DA/RA represents WT CspC-CPD-His6 with untagged CspAD1008A/R1036A; and CspC QE/TE represents CspCQ516E/T520E-CPD-His6 with untagged WT CspA. Inputs correspond to the SEC traces shown in Fig 7D. CspA/total (%) = 100 × [CspA signal intensity/(CspA + CspC signal intensities)]. The data shown are representative of a minimum of two independent replicates. The raw gel images can be found in S1 Raw Images. (TIF) [file pbio.3003610.s017.tif]

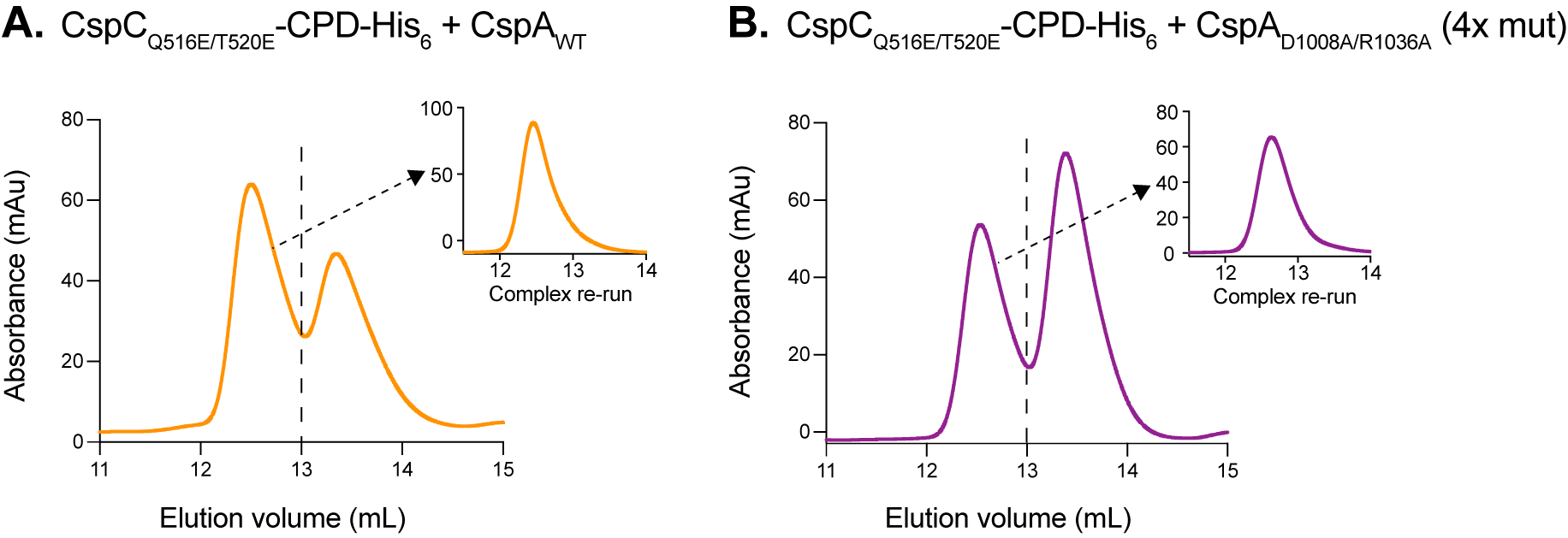

Supplement: S13 Fig — Size exclusion chromatography (SEC) analysis of the CspCQ516E/T520E-CPD-His6:CspA (A) and CspCQ516E/T520E-CPD-His6:CspAD1008A/R1036A (B) co-affinity purifications. The dashed line indicates the separation between the two peaks. (A and B insets) Analysis of the stability of SEC-purified CspC-CPD-His6:CspA mutant complexes. The complexes were purified from the 12.0 to 13.0 mL fraction and then re-analyzed using SEC. The data underlying the panels in this figure can be found in S1 Data. (TIF) [file pbio.3003610.s018.tif]

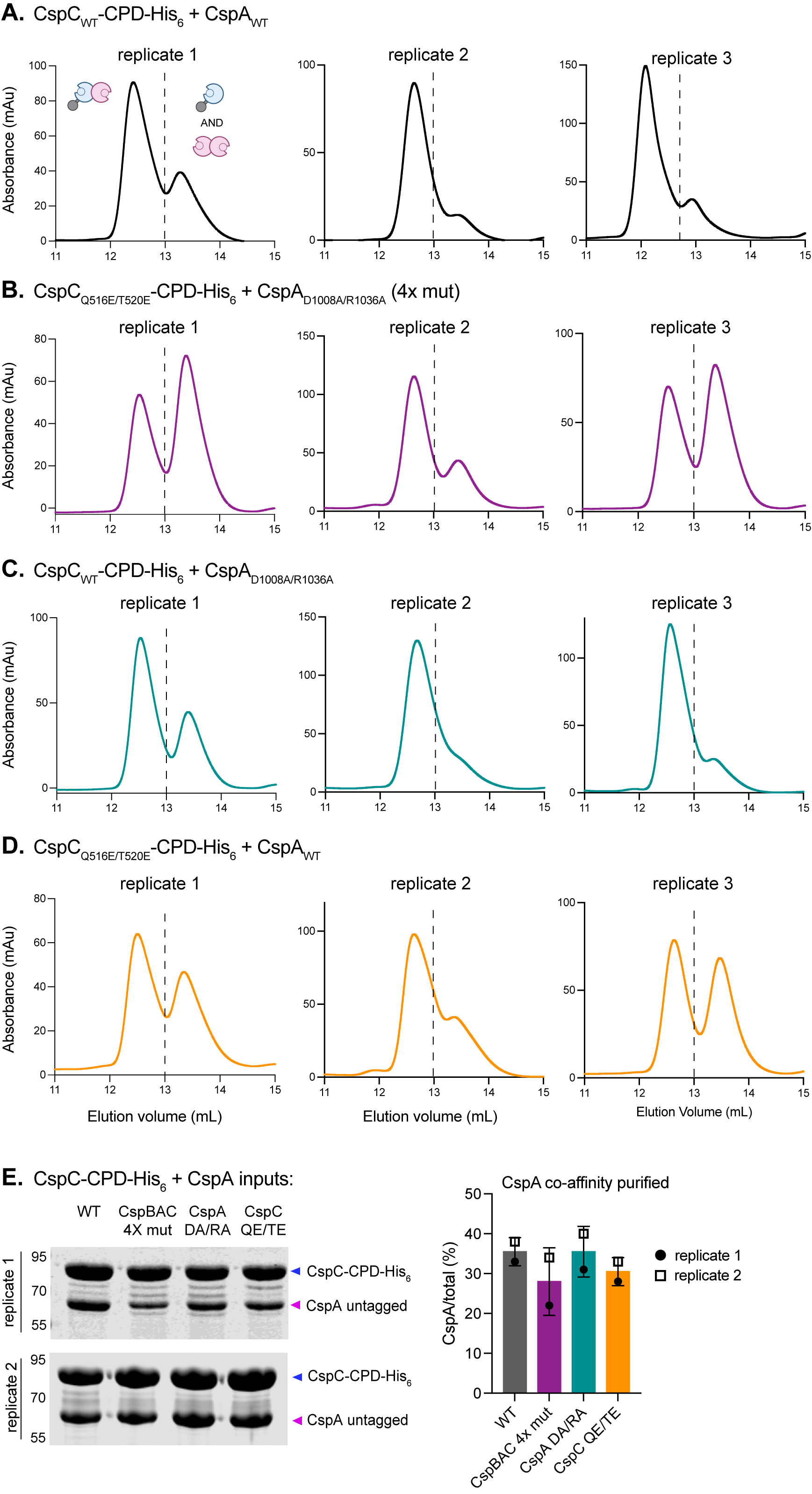

Supplement: S14 Fig — (A–D) Size exclusion chromatography analyses of WT and mutant CspC-CPD-His6:CspA co-affinity purifications. SEC replicates from three independent co-affinity purifications. (E) (left) Inputs for replicates 1 and 2 run on SDS-PAGE and stained with Coomassie. (right) Quantification of the percentage of CspA from SEC inputs. CspA/total (%) = 100 × [CspA signal intensity/(CspA + CspC signal intensities)]. The data underlying panels A–D and E (right) can be found in S1 Data. The raw gel image in panel E (left) can be found in S1 Raw Images. (TIF) [file pbio.3003610.s019.tif]

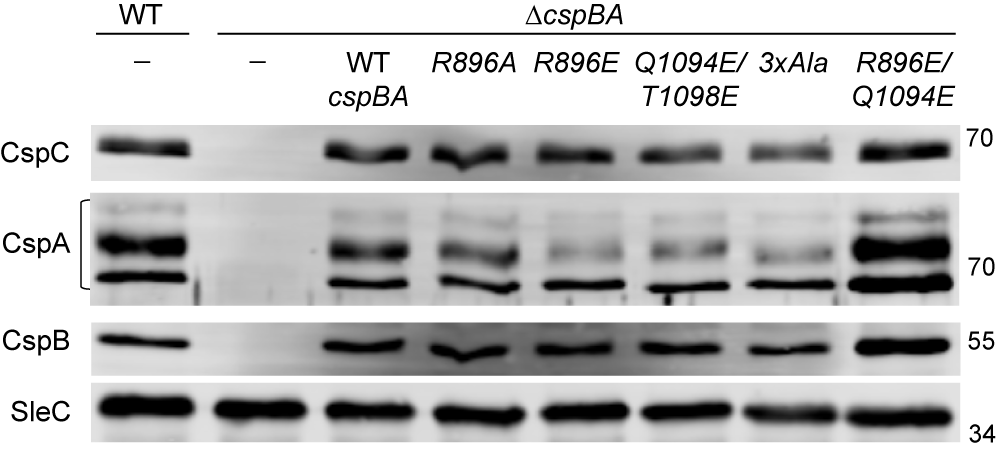

Supplement: S15 Fig — Western blot analyses of Csp levels in mutant spores. ∆cspBA/3xAla = ∆cspBA/cspBAR896A/Q1094A/T1098A. Multiple isoforms of CspA are observed. * indicates a non-specific band. SleC was used as a load control. The data shown are representative of a minimum of three independent replicates. The raw gel image can be found in S1 Raw Images. (TIF) [file pbio.3003610.s020.tif]

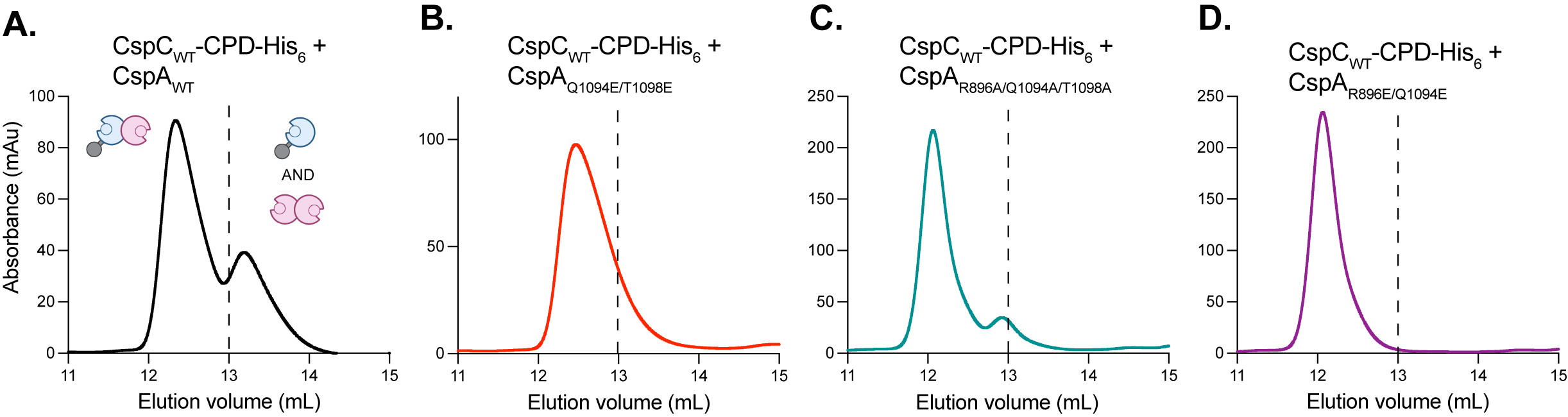

Supplement: S16 Fig — Size exclusion chromatography analysis of CspC-CPD-His6 and CspA variant co-affinity purifications. The dashed line indicates the separation between the two peaks. All data shown are representative of two independent replicates. The data underlying the panels in this figure can be found in S1 Data. (TIF) [file pbio.3003610.s021.tif]

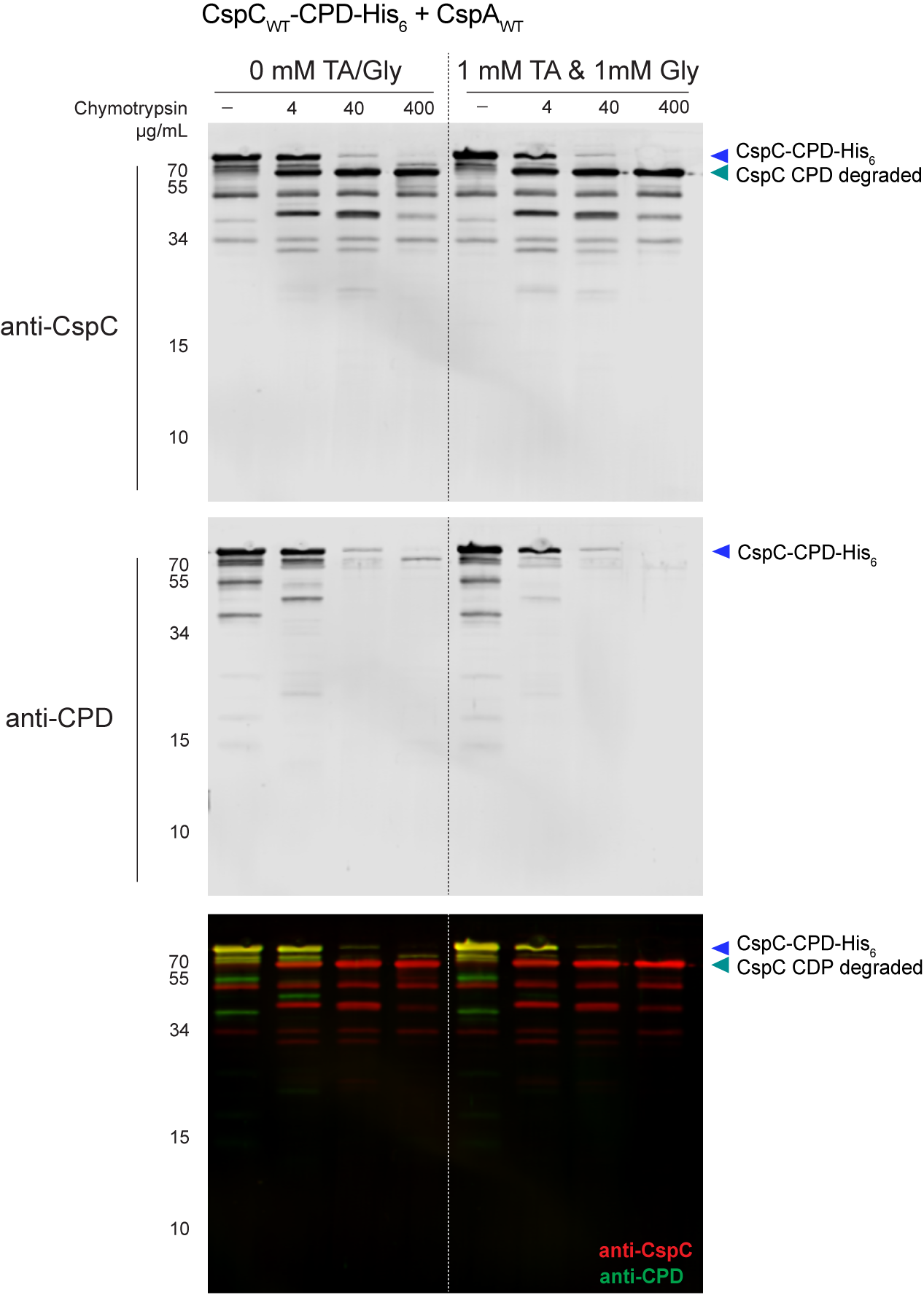

Supplement: S17 Fig — Western blot of limited proteolysis analyses of purified CspC-CPD-His6:CspA heterodimer using the indicated concentrations of chymotrypsin and anti-CspC or anti-CPD antibodies. The raw gel images in this figure can be found in S1 Raw Images. (TIF) [file pbio.3003610.s022.tif]

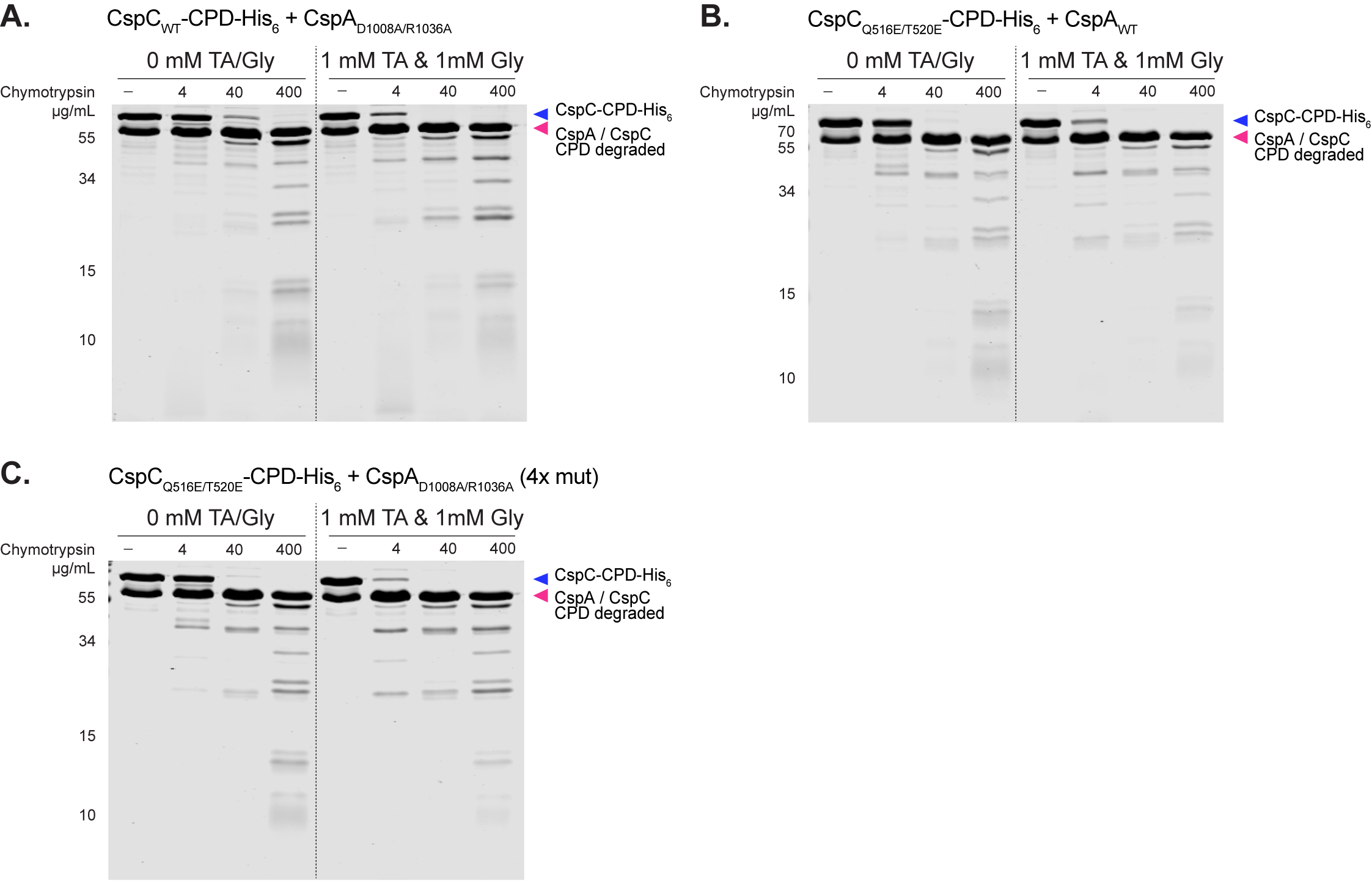

Supplement: S18 Fig — Limited proteolysis analyses of purified CspC-CPD-His6:CspA heterodimer mutants in the presence and absence of the indicated concentrations of taurocholate (TA) and glycine (Gly). Cleavage of the complexes at 40 μg/mL chymotrypsin is due to digestion of the CPD-His6 tag (S17 Fig). The raw gel images in this figure can be found in S1 Raw Images. (TIF) [file pbio.3003610.s023.tif]

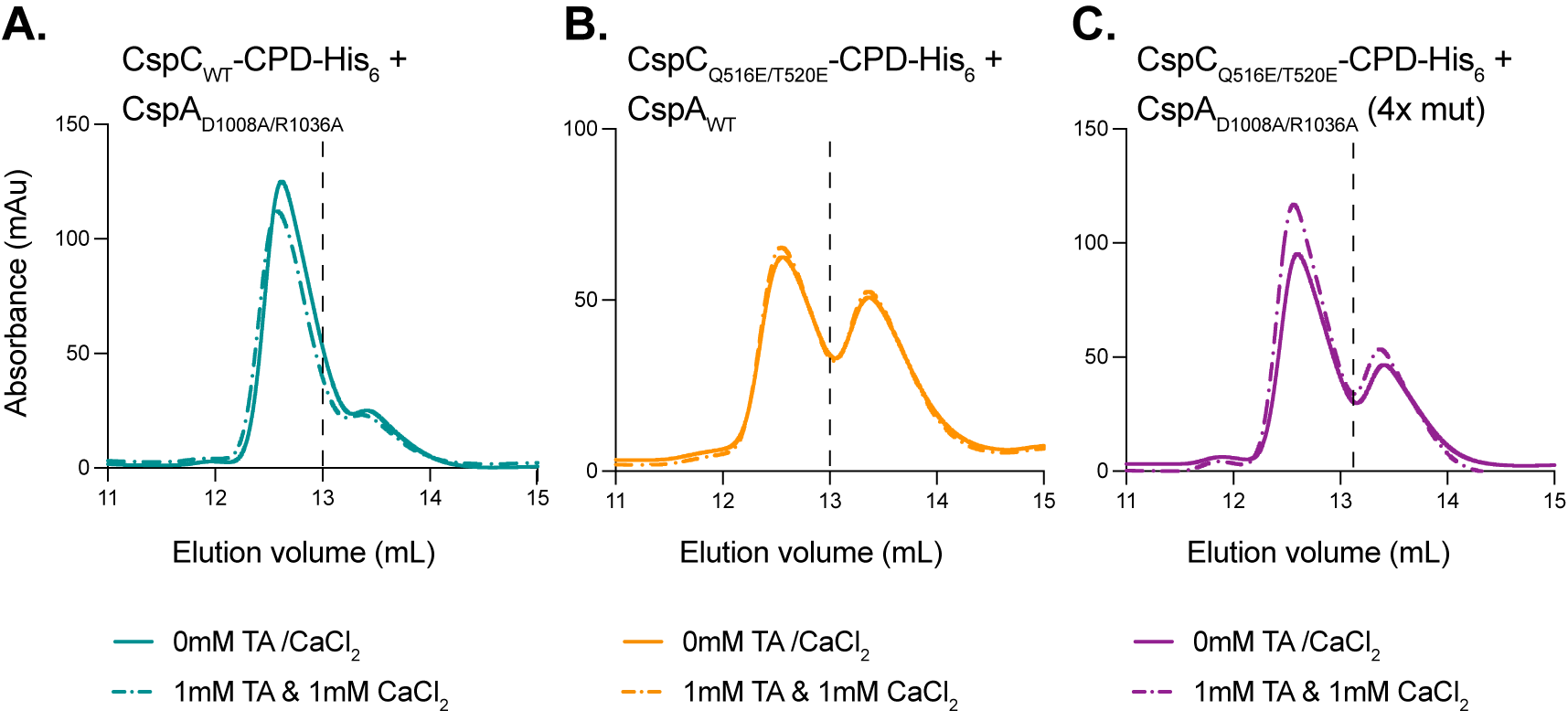

Supplement: S19 Fig — Size exclusion chromatography analyses of mutant CspC-CPD-His6:CspA co-affinity purifications in the presence or absence of the indicated concentrations of TA and CaCl2. The data underlying the panels in this figure can be found in S1 Data. (TIF) [file pbio.3003610.s024.tif]

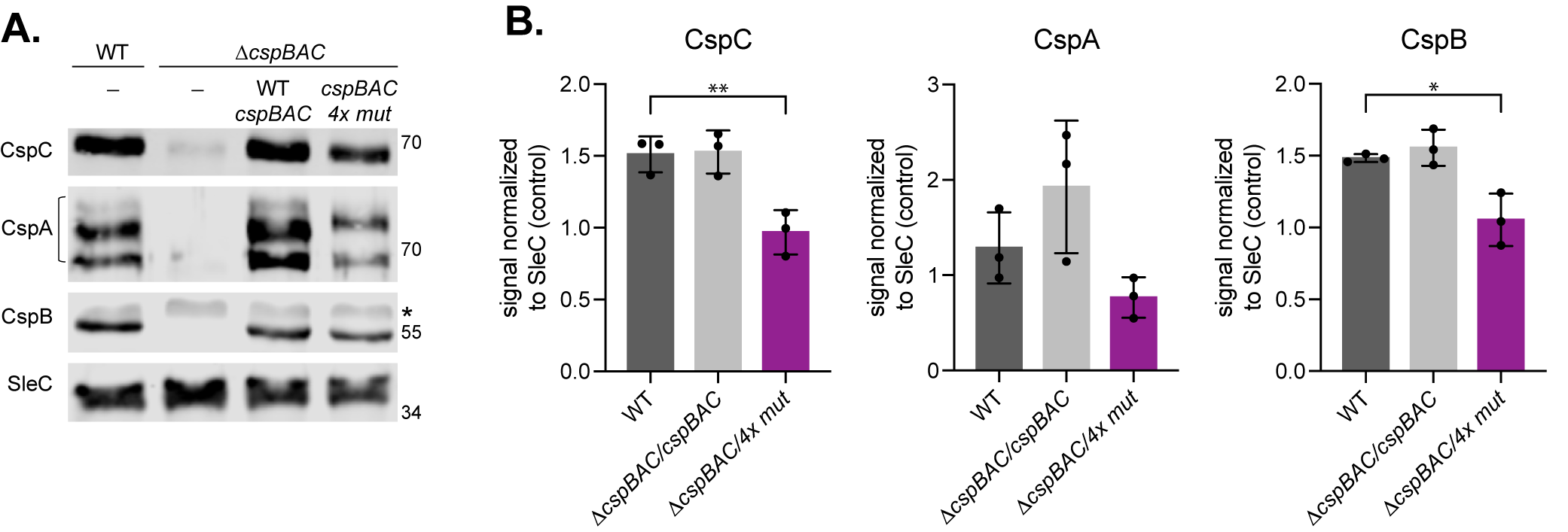

Supplement: S20 Fig — (A) western blot analyses of Csp levels in mutant spores. ∆cspBAC/4X mut = ∆cspBAC/cspBAD1008A/R1036A-cspCQ516E/T520E Multiple isoforms of CspA are observed. Multiple isoforms of CspA are observed. * indicates a non-specific band. SleC was used as a load control. (B) Quantification of western blot analyses. Protein signal intensities were normalized to the SleC loading control intensities. Statistical significance relative to WT was determined using a one-way ANOVA and Dunnett’s multiple comparisons test. ** p < 0.01, * < 0.1. The data shown are representative of a minimum of three independent replicates. The raw gel image in panel A can be found in S1 Raw Images. The data underlying panel B can be found in S1 Data. (TIF) [file pbio.3003610.s025.tif]

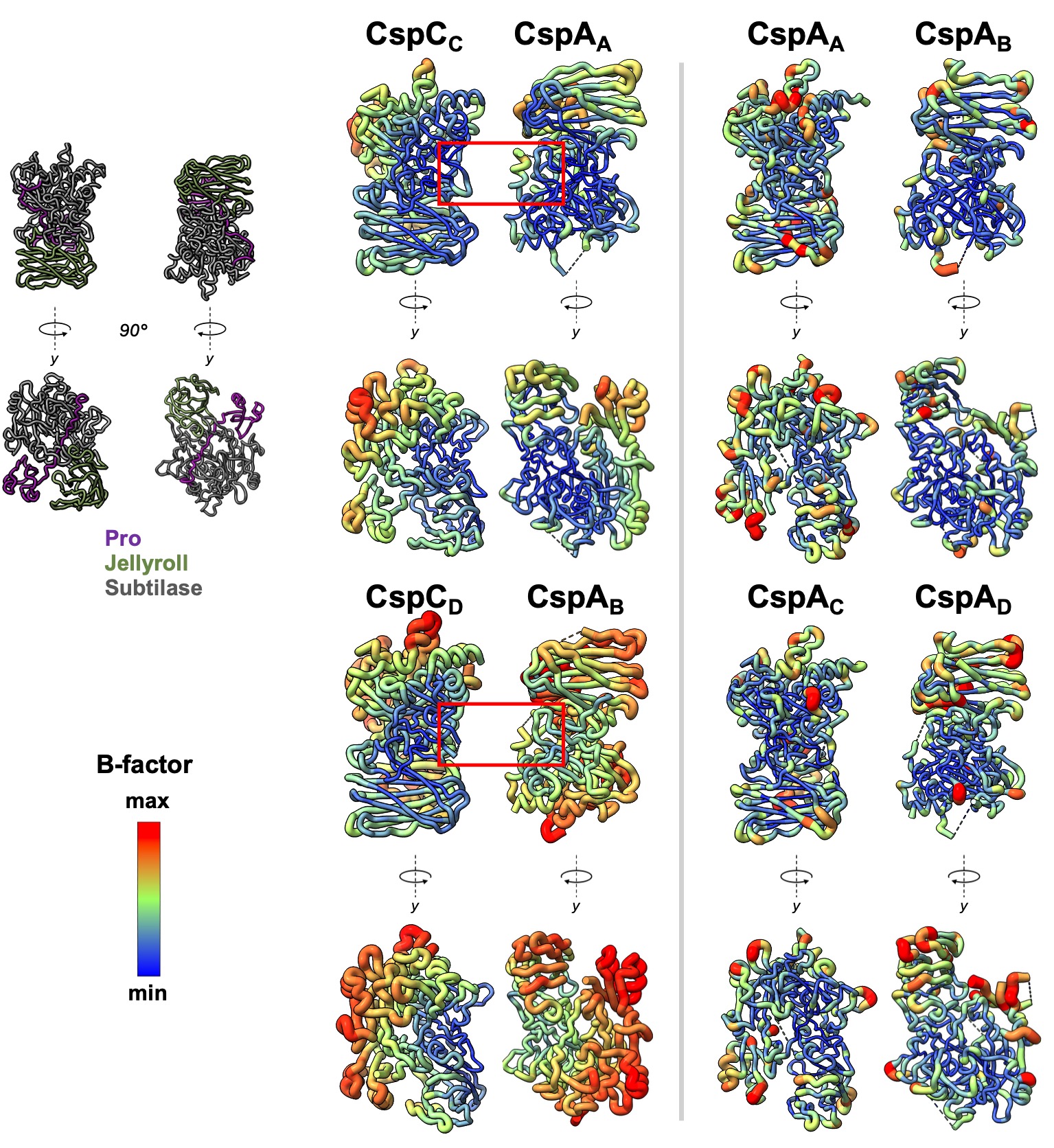

Supplement: S21 Fig — Protomers for the CspC:CspA heterodimer (left) and the CspA homodimer (right) colored and rendered by B-factor in ChimeraX reveal dynamic regions across the complexes. The CspC protomer’s subtilase and prodomains display high B-factors relative to the rest of the complex. Likewise, the CspA prodomain exhibits high B-factors in both the hetero- and homodimer. Interestingly, the CspC/CspA heterodimer displays a wider B-factor range than does the CspA homodimer. Furthermore, the two CspC:CspA heterodimers in the asymmetric unit exhibit strikingly different B-factor distributions. The C/A heterodimer has a more ordered CspA protomer than does the D/B heterodimer. On the other hand, B-factor distributions are generally similar across the two CspA asymmetric unit homodimers. The red box indicates the regions containing the central salt bridges of the CspC:CspA heterodimer (Figs 2C and 3A). (JPG) [file pbio.3003610.s026.jpg]
