# Supplementary material for: The CspC:CspA heterodimer transduces germinant and co-germinant signals during Clostridioides difficile spore germination
Source: PLoS Biol. 2026 Feb 2;24(2):e3003610. doi: 10.1371/journal.pbio.3003610 (PMC12880746; doi:10.1371/journal.pbio.3003610)
Supplement: S1 Raw Images — (DOCX) [file pbio.3003610.s028.docx]

SDS-PAGE gels

Figure 1A (left):


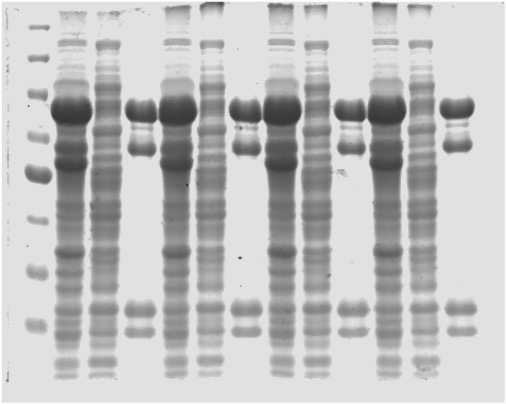


SDS-PAGE gel of co-affinity purifications of CspC-CPD-His_6_ as the bait and untagged CspB and CspA as the prey stained with Coomassie and visualized using an Odyssey LiCOR CLx. Ladder from top to bottom (kDa): 250, 130, 95, 70, 55, 43, 34, 26. Lanes from left to right: induced fraction, cleared lysate, elution. Lanes 5 – 13 not applicable to study.

Figure 1A (right):


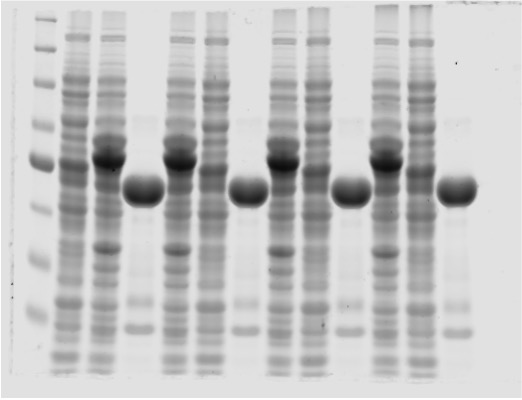


SDS-PAGE gel of co-affinity purifications of GFP-CPD-His_6_ as the bait and untagged CspB and CspA as the prey stained with Coomassie and visualized using an Odyssey LiCOR CLx. Ladder from top to bottom (kDa): 250, 130, 95, 70, 55, 43, 34, 26. Lanes from left to right: induced fraction, cleared lysate, elution. Lanes 5 – 13 not applicable to study.

Figure 1B (top):


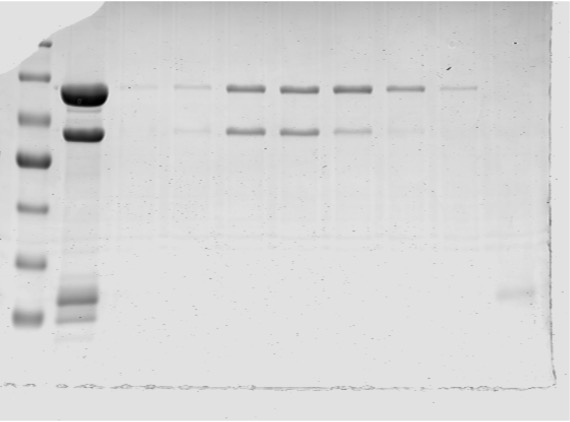


SEC elution fractions resolved using SDS-PAGE, stained with Coomassie, and visualized using an Odyssey LiCOR CLx. Ladder from top to bottom (kDa): 250, 130, 95, 70, 55, 43, 34, 26. Lanes from left to right: input, fractions (mL): 11.5-12, 12-12.5, 12.5-13, 13-13.5, 13.5-14, 14-14.5, 14.5-15.

Figure 1B (center):


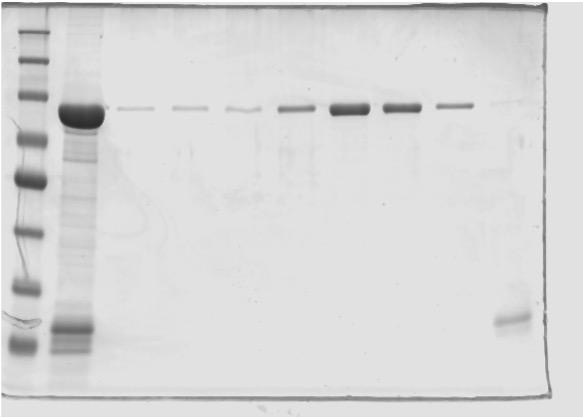


SEC elution fractions resolved using SDS-PAGE, stained with Coomassie, and visualized using an Odyssey LiCOR CLx. Ladder from top to bottom (kDa): 250, 130, 95, 70, 55, 43, 34, 26. Lanes from left to right: input, fractions (mL): 11.5-12, 12-12.5, 12.5-13, 13-13.5, 13.5-14, 14-14.5, 14.5-15.

Figure 1B (bottom):


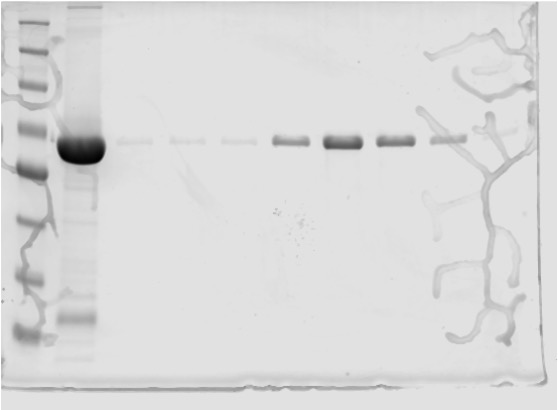


SEC elution fractions resolved using SDS-PAGE, stained with Coomassie, and visualized using an Odyssey LiCOR CLx. Ladder from top to bottom (kDa): 250, 130, 95, 70, 55, 43, 34, 26. Lanes from left to right: input, fractions (mL): 11.5-12, 12-12.5, 12.5-13, 13-13.5, 13.5-14, 14-14.5, 14.5-15.

Figure 1C (top):


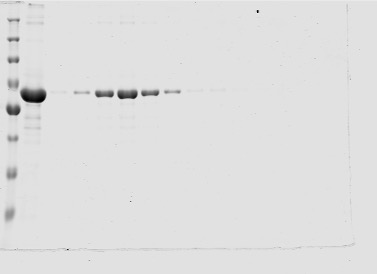


SEC elution fractions resolved using SDS-PAGE, stained with Coomassie, and visualized using an Odyssey LiCOR CLx. Ladder from top to bottom (kDa): 250, 130, 95, 70, 55, 43, 34, 26. Lanes from left to right: input, fractions (mL): 12-12.5, 12.5-13, 13-13.5, 13.5-14, 14-14.5, 14.5-15, 15-15.5, 15.5-16, 16-16.5, 16.5-17.

Figure 1C (center):


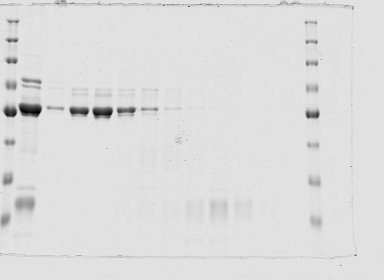


SEC elution fractions resolved using SDS-PAGE, stained with Coomassie, and visualized using an Odyssey LiCOR CLx. Ladder from top to bottom (kDa): 250, 130, 95, 70, 55, 43, 34, 26. Lanes from left to right: input, fractions (mL): 12-12.5, 12.5-13, 13-13.5, 13.5-14, 14-14.5, 14.5-15, 15-15.5, 15.5-16, 16-16.5, 16.5-17, ladder.

Figure 1C (bottom):


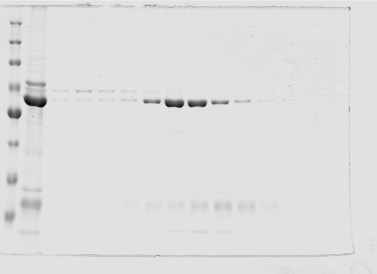


SEC elution fractions resolved using SDS-PAGE, stained with Coomassie, and visualized using an Odyssey LiCOR CLx. Ladder from top to bottom (kDa): 250, 130, 95, 70, 55, 43, 34, 26. Lanes from left to right: input, fractions (mL): 12-12.5, 12.5-13, 13-13.5, 13.5-14, 14-14.5, 14.5-15, 15-15.5, 15.5-16, 16-16.5, 16.5-17.

Figure 1D (CspA – mixed):


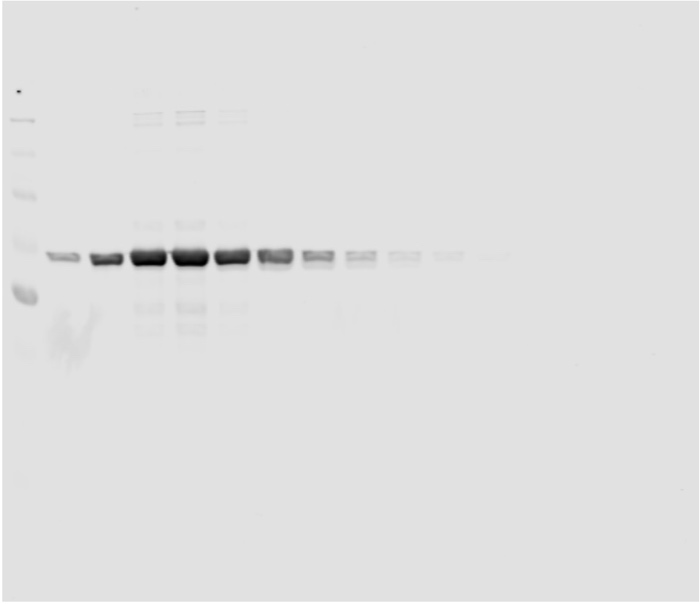


SEC elution fractions resolved using SDS-PAGE, stained with Coomassie, and visualized using an Odyssey LiCOR CLx. Ladder from top to bottom (kDa): 250, 130, 95, 70, 55. Lanes from left to right: fractions (mL): 12-12.5, 12.5-13, 13-13.5, 13.5-14, 14-14.5, 14.5-15, 15-15.5, 15.5-16, 16-16.5, 16.5-17.

Figure 1D (CspC – mixed):


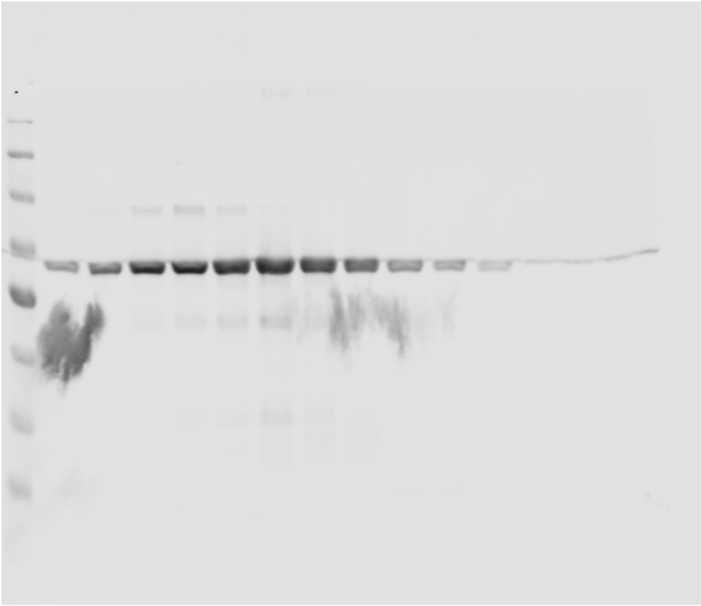


SEC elution fractions resolved using SDS-PAGE, stained with Coomassie, and visualized using an Odyssey LiCOR CLx. Ladder from top to bottom (kDa): 250, 130, 95, 70, 55, 43, 34, 26. Lanes from left to right: fractions (mL): 12-12.5, 12.5-13, 13-13.5, 13.5-14, 14-14.5, 14.5-15, 15-15.5, 15.5-16, 16-16.5, 16.5-17.

Figure 1D (CspA – individual):


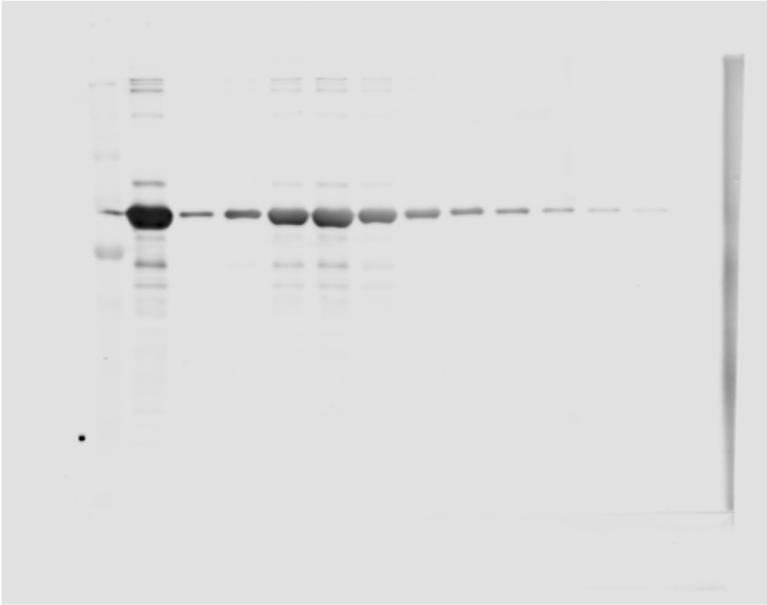


SEC elution fractions resolved using SDS-PAGE, stained with Coomassie, and visualized using an Odyssey LiCOR CLx. Ladder from top to bottom (kDa): 250, 130, 95, 70, 55, 43. Lanes from left to right: input, fractions (mL): 12-12.5, 12.5-13, 13-13.5, 13.5-14, 14-14.5, 14.5-15, 15-15.5, 15.5-16, 16-16.5, 16.5-17.

Figure 1D (CspC – individual):


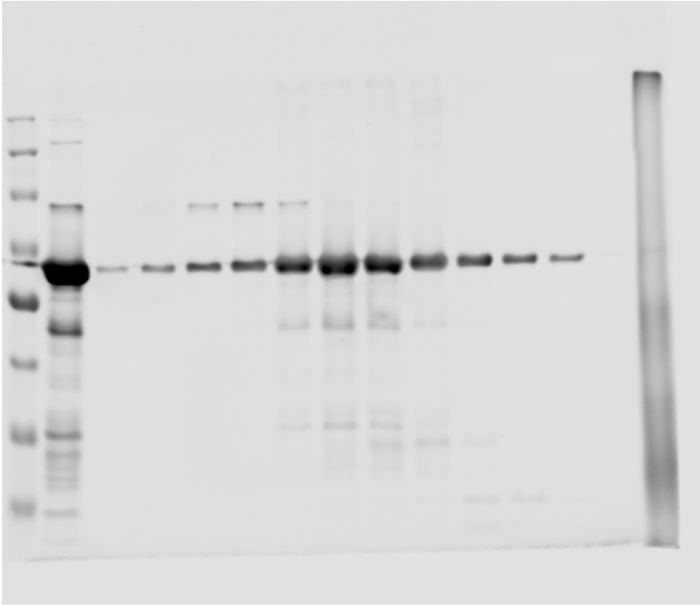


SEC elution fractions resolved using SDS-PAGE, stained with Coomassie, and visualized using an Odyssey LiCOR CLx. Ladder from top to bottom (kDa): 250, 130, 95, 70, 55, 43, 34, 26. Lanes from left to right: input, fractions (mL): 12-12.5, 12.5-13, 13-13.5, 13.5-14, 14-14.5, 14.5-15, 15-15.5, 15.5-16, 16-16.5, 16.5-17.

Figure 6A (left):


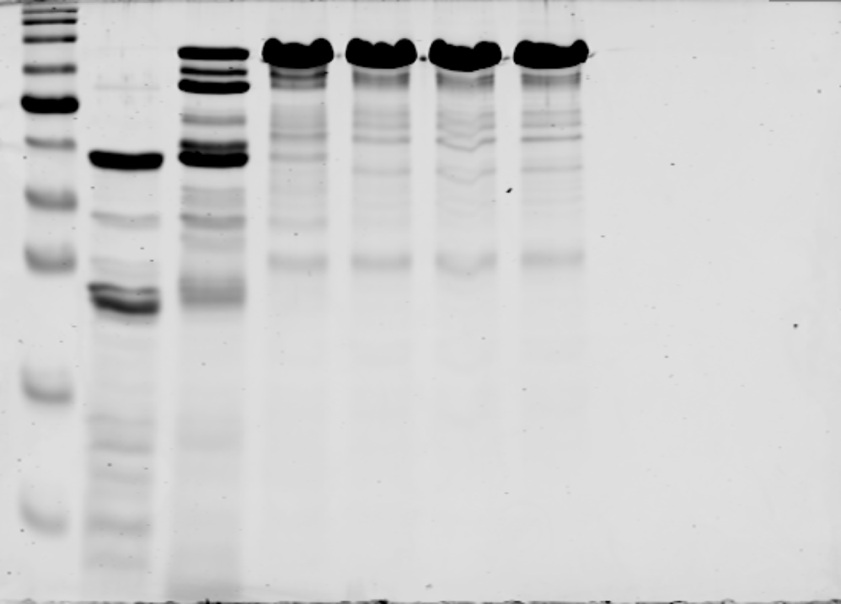


Limited proteolysis resolved using SDS-PAGE, stained with Coomassie, and visualized using an Odyssey LiCOR CLx. Ladder from top to bottom (kDa): 250, 130, 95, 70, 55, 43, 34, 26, 15, 11. Lanes from left to right: chymotrypsin (µg/mL): 40, 4, 0.4, 0.04, 0.004, 0.

Figure 6A (center):


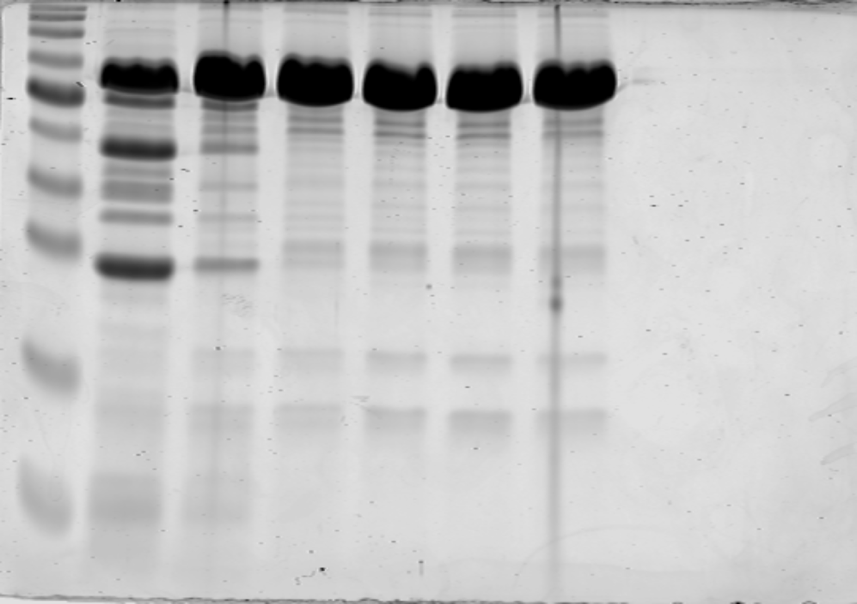


Limited proteolysis resolved using SDS-PAGE, stained with Coomassie, and visualized using an Odyssey LiCOR CLx. Ladder from top to bottom (kDa): 250, 130, 95, 70, 55, 43, 34, 26, 15, 11. Lanes from left to right: chymotrypsin (µg/mL): 40, 4, 0.4, 0.04, 0.004, 0.

Figure 6A (right):


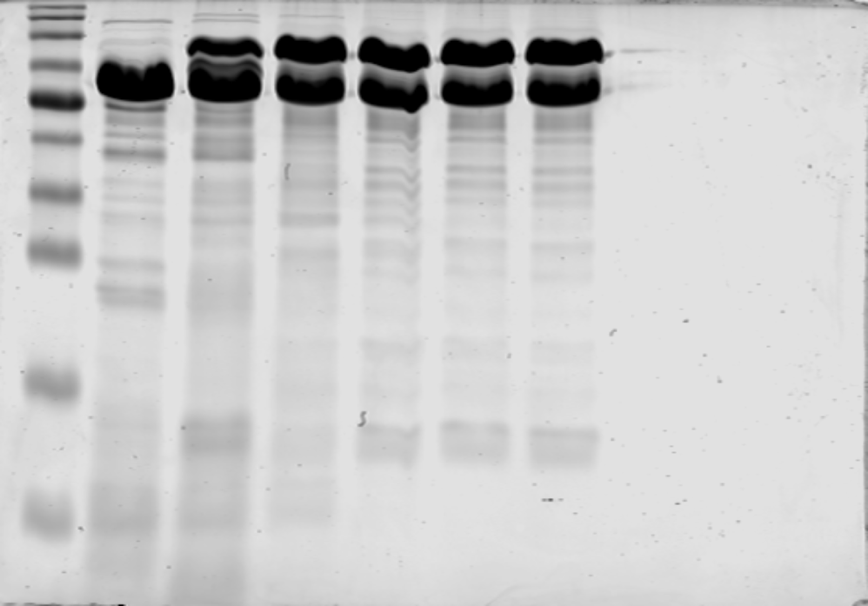


Limited proteolysis resolved using SDS-PAGE, stained with Coomassie, and visualized using an Odyssey LiCOR CLx. Ladder from top to bottom (kDa): 250, 130, 95, 70, 55, 43, 34, 26, 15, 11. Lanes from left to right: chymotrypsin (µg/mL): 40, 4, 0.4, 0.04, 0.004, 0.

Figure 6B:


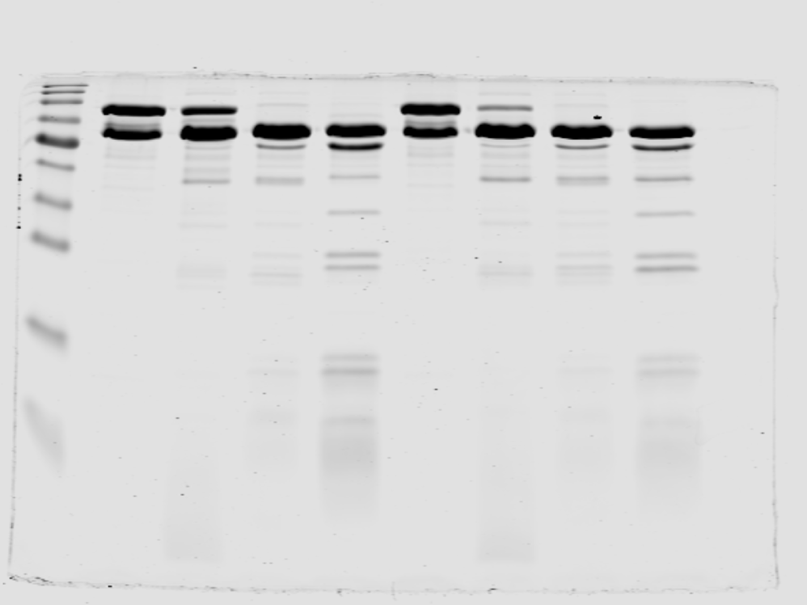


Limited proteolysis resolved using SDS-PAGE, stained with Coomassie, and visualized using an Odyssey LiCOR CLx. Ladder from top to bottom (kDa): 250, 130, 95, 70, 55, 43, 34, 26, 15, 11. Lanes from left to right: chymotrypsin (µg/mL): 0, 4, 40, 400, 0 + 1 mM TA & 1 mM glycine, 4 + 1 mM TA & 1 mM glycine, 40 + 1 mM TA & 1 mM glycine, 400 + 1 mM TA & 1 mM glycine.

Figure S1:


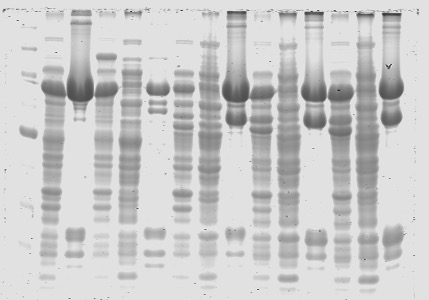


Co-affinity purifications resolved using SDS-PAGE, stained with Coomassie, and visualized using an Odyssey LiCOR CLx. Ladder from top to bottom (kDa): 250, 130, 95, 70, 55, 43, 34, 26. Lanes from left to right: lanes 2 – 3 not applicable to study, CspC-CPD-His_6_ + CspBA: input, cleared lysate, elution; CspC-CPD-His_6_ + CspA + CspB: input, cleared lysate, elution. Lanes 10 – 15 not applicable to study.

Figure S2 (top):


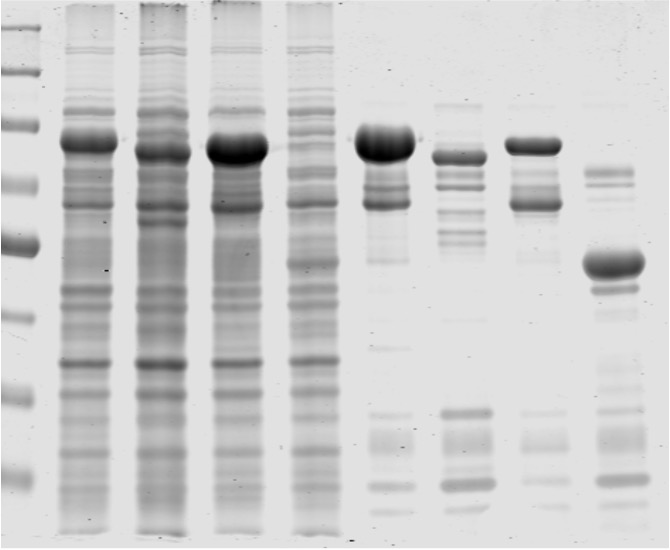


Co-affinity purifications resolved using SDS-PAGE, stained with Coomassie, and visualized using an Odyssey LiCOR CLx. Ladder from top to bottom (kDa): 250, 130, 95, 70, 55, 43, 34, 26 (ladder cut off on the left during imaging, but MW was not obscured). Lanes from left to right: untagged CspA + bait (input): CspA-CPD-His_6_, CspB-CPD-His_6_, CspC-CPD-His_6_, GFP-CPD-His_6_; untagged CspA + bait (elution): CspA-CPD-His_6_, CspB-CPD-His_6_, CspC-CPD-His_6_, GFP-CPD-His_6_.

Figure S2 (center):


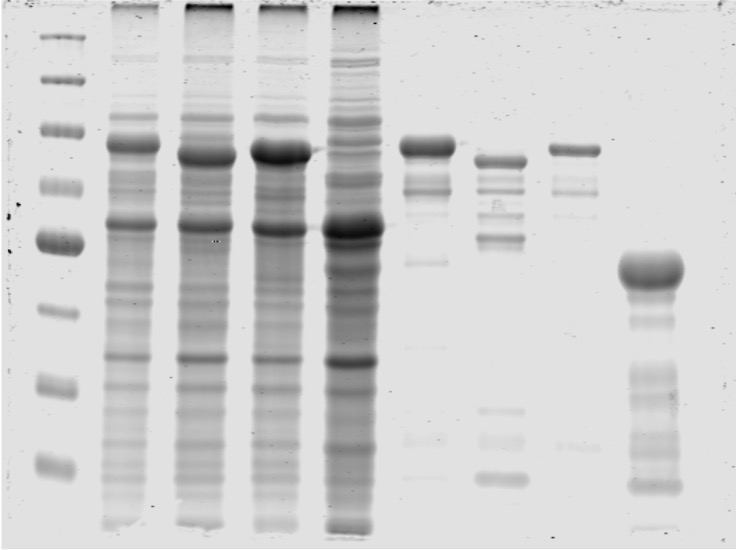


Co-affinity purifications resolved using SDS-PAGE, stained with Coomassie, and visualized using an Odyssey LiCOR CLx. Ladder from top to bottom (kDa): 250, 130, 95, 70, 55, 43, 34. Lanes from left to right: untagged CspB + bait (input): CspA-CPD-His_6_, CspB-CPD-His_6_, CspC-CPD-His_6_, GFP-CPD-His_6_; untagged CspB + bait (elution): CspA-CPD-His_6_, CspB-CPD-His_6_, CspC-CPD-His_6_, GFP-CPD-His_6_.

Figure S2 (bottom):


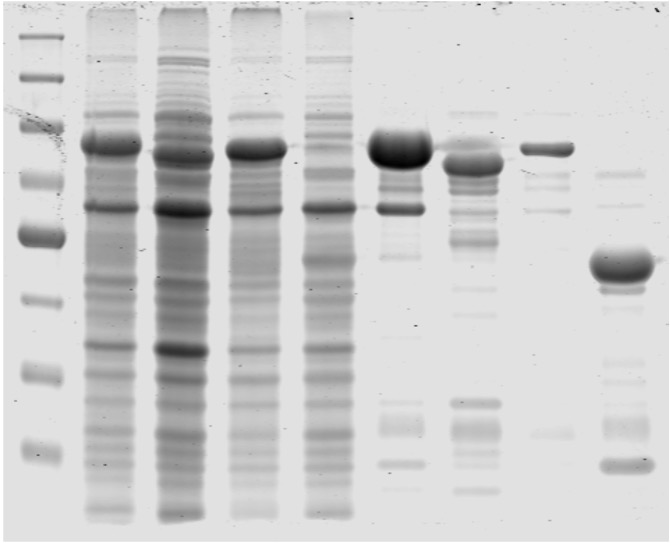


Co-affinity purifications resolved using SDS-PAGE, stained with Coomassie, and visualized using an Odyssey LiCOR CLx. Ladder from top to bottom (kDa): 250, 130, 95, 70, 55, 43, 34, 26. Lanes from left to right: untagged CspC + bait (input): CspA-CPD-His_6_, CspB-CPD-His_6_, CspC-CPD-His_6_, GFP-CPD-His_6_; untagged CspC + bait (elution): CspA-CPD-His_6_, CspB-CPD-His_6_, CspC-CPD-His_6_, GFP-CPD-His_6_.

Figure S10A (anti-CspC):


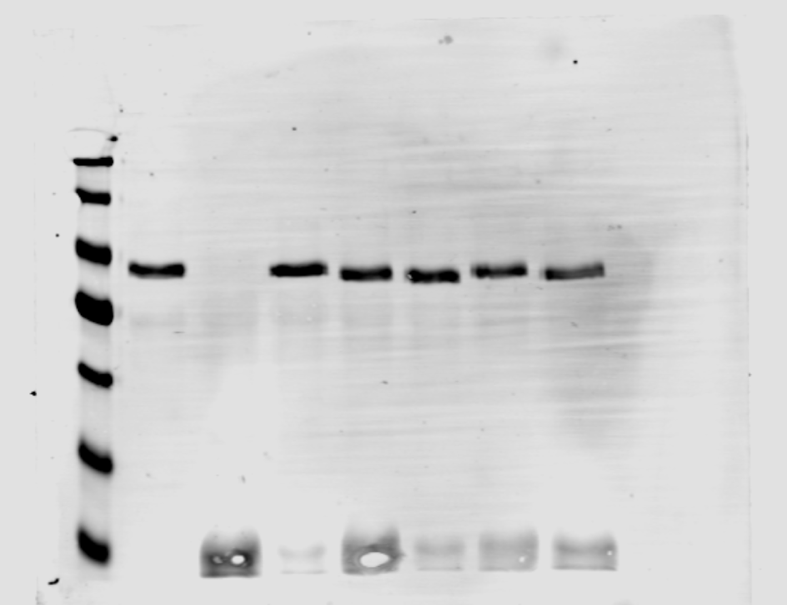


Western blot visualized using an Odyssey LiCOR CLx (2ºAb 680, excitation 700 nm). Ladder from top to bottom (kDa): 250, 130, 95, 70, 55, 43, 34, 26. Lanes from left to right: WT, *∆cspC*, *∆cspC/cspC*, *∆cspC/cspC*_D429A_, *∆cspC/cspC*_D429K_, *∆cspC/cspC*_R456A_, *∆cspC/cspC*_D429A/R456A_.

Figure S10A (anti-CspA):


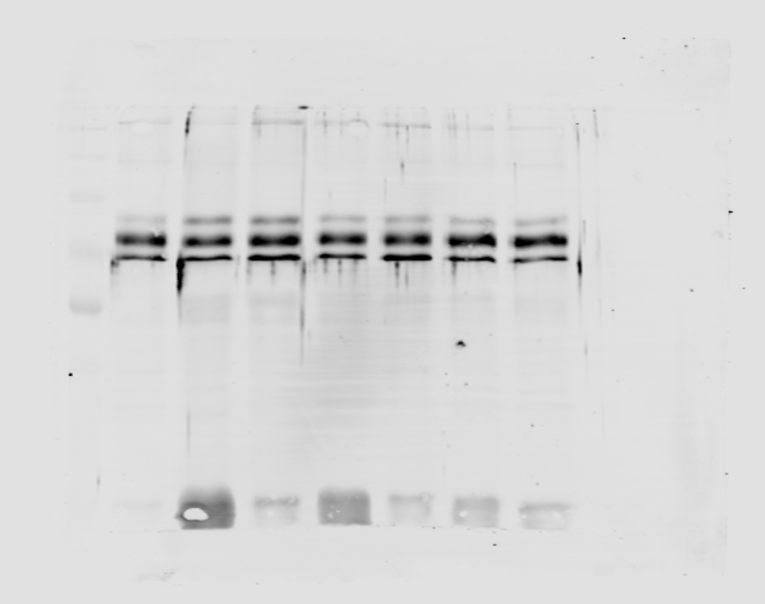


Western blot visualized using an Odyssey LiCOR CLx (2ºAb IRD800, excitation 800 nm). Ladder from top to bottom (kDa): 250, 130, 95, 70, 55. Lanes from left to right: WT, *∆cspC*, *∆cspC/cspC*, *∆cspC/cspC*_D429A_, *∆cspC/cspC*_D429K_, *∆cspC/cspC*_R456A_, *∆cspC/cspC*_D429A/R456A_.

Figure S10A (anti-CspB):


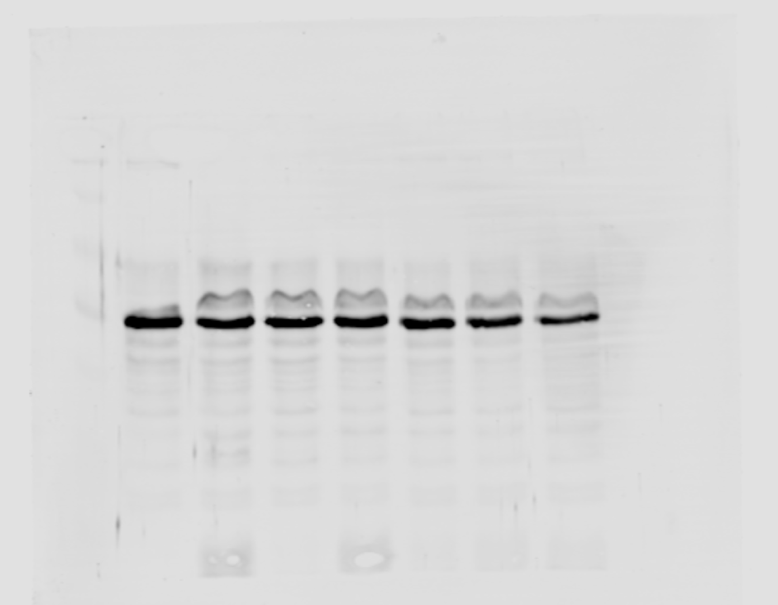


Western blot visualized using an Odyssey LiCOR CLx (2ºAb IRD800, excitation 800 nm). Ladder from top to bottom (kDa): 250, 130, 95, 70, 55. Lanes from left to right: WT, *∆cspC*, *∆cspC/cspC*, *∆cspC/cspC*_D429A_, *∆cspC/cspC*_D429K_, *∆cspC/cspC*_R456A_, *∆cspC/cspC*_D429A/R456A_.

Figure S10 (anti-SleC):


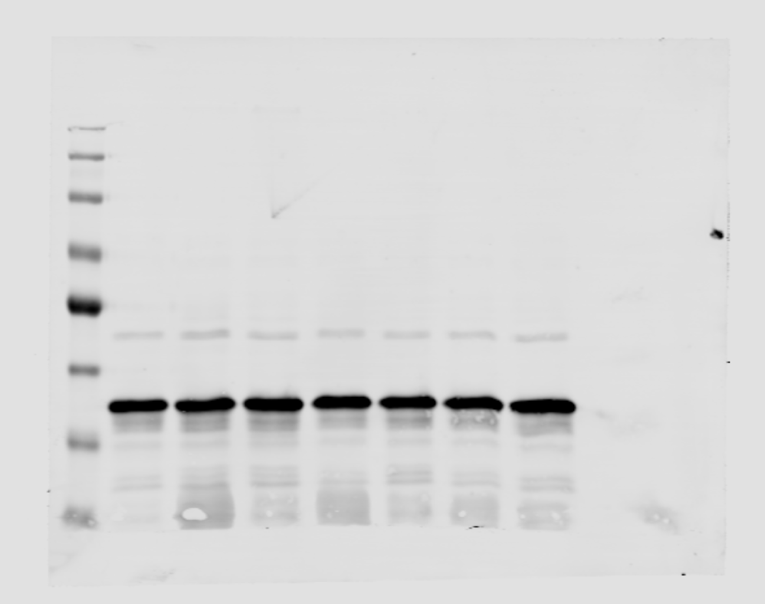


Western blot visualized using an Odyssey LiCOR CLx (2ºAb IRD680, excitation 700 nm). Ladder from top to bottom (kDa): 250, 130, 95, 70, 55, 43, 34, 26. Lanes from left to right: WT, *∆cspC*, *∆cspC/cspC*, *∆cspC/cspC*_D429A_, *∆cspC/cspC*_D429K_, *∆cspC/cspC*_R456A_, *∆cspC/cspC*_D429A/R456A_.

Figure S11A (anti-CspC):


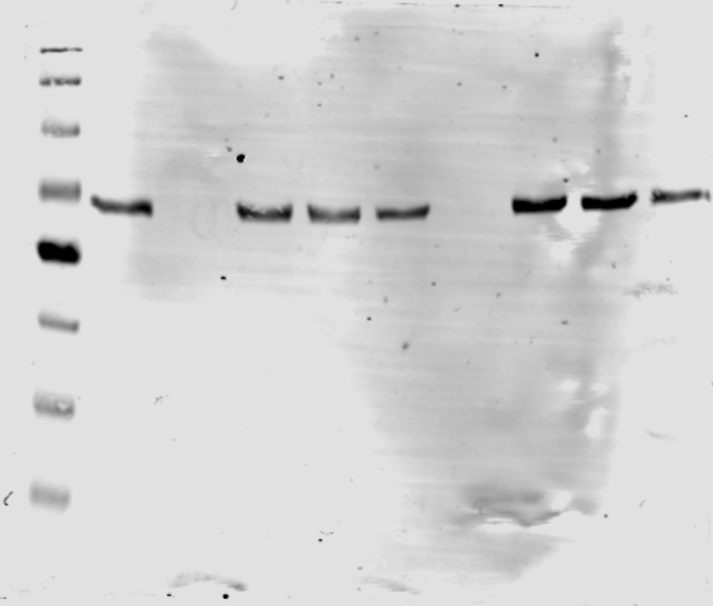


Western blot visualized using an Odyssey LiCOR CLx (2ºAb IRD680, excitation 700 nm). Ladder from top to bottom (kDa): 250, 130, 95, 70, 55, 43, 34, 26. Lanes from left to right: WT, *∆cspC*, *∆cspC/cspC*, *∆cspC/cspC*_Q516A/T540A_, *∆cspC/cspC*_Q516E/T520E_, *∆cspBAC, ∆cspBAC/cspBAC*, *∆cspBAC/cspBA*_R896A_ *cspC*_Q516A/T520A_ (*3xAla*), *∆cspBAC/cspBA*_R896E_ *cspC*_Q516E/T520E_ (*3xGlu*).

Figure S11A (anti-CspA):


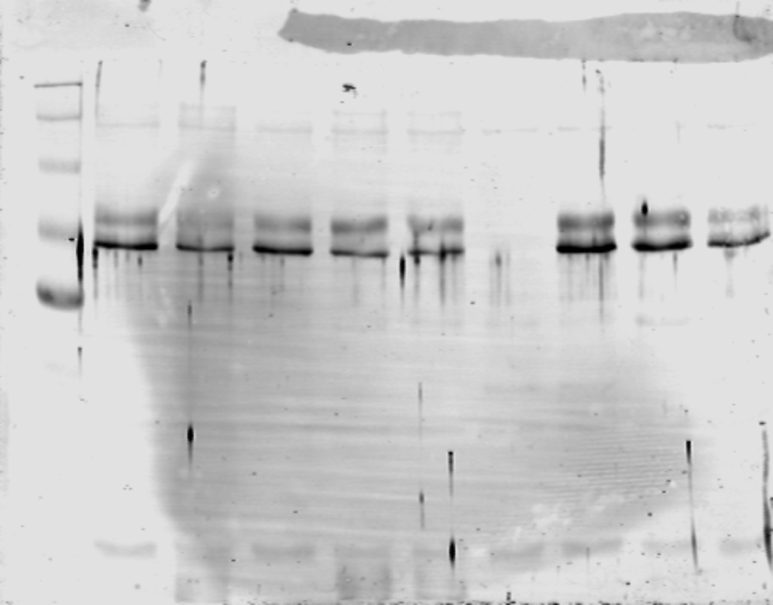


Western blot visualized using an Odyssey LiCOR CLx (2ºAb IRD800, excitation 800 nm). Ladder from top to bottom (kDa): 250, 130, 95, 70, 55. Lanes from left to right: WT, *∆cspC*, *∆cspC/cspC*, *∆cspC/cspC*_Q516A/T540A_, *∆cspC/cspC*_Q516E/T520E_, *∆cspBAC, ∆cspBAC/cspBAC*, *∆cspBAC/cspBA*_R896A_ *cspC*_Q516A/T520A_ (*3xAla*), *∆cspBAC/cspBA*_R896E_ *cspC*_Q516E/T520E_ (*3xGlu*).

Figure S11A (anti-CspB):


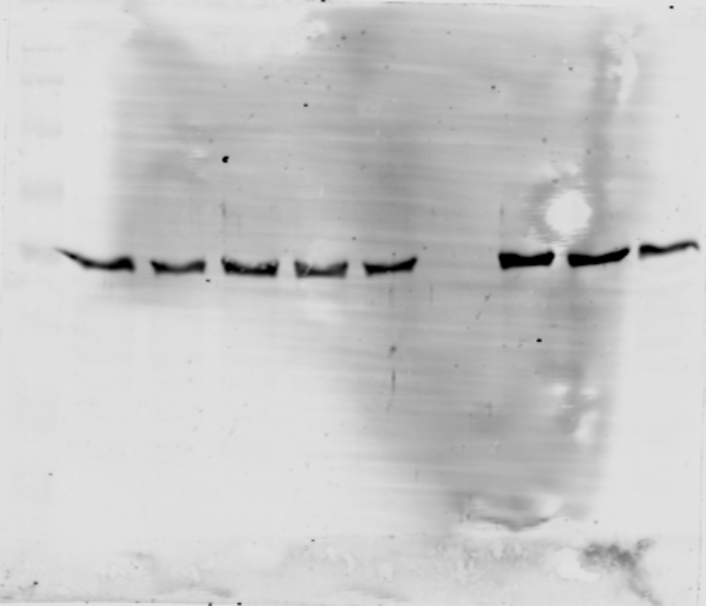


Western blot visualized using an Odyssey LiCOR CLx (2ºAb IRD800, excitation 800 nm). Ladder from top to bottom (kDa): 250, 130, 95, 70, 55. Lanes from left to right: WT, *∆cspC*, *∆cspC/cspC*, *∆cspC/cspC*_Q516A/T540A_, *∆cspC/cspC*_Q516E/T520E_, *∆cspBAC, ∆cspBAC/cspBAC*, *∆cspBAC/cspBA*_R896A_ *cspC*_Q516A/T520A_ (*3xAla*), *∆cspBAC/cspBA*_R896E_ *cspC*_Q516E/T520E_ (*3xGlu*).

Figure S11A (anti-SleC):


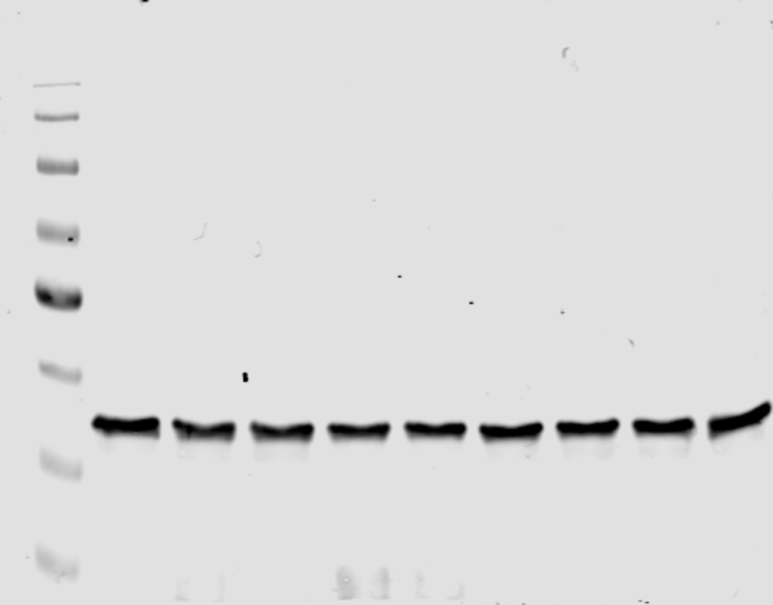


Western blot visualized using an Odyssey LiCOR CLx (2ºAb IRD680, excitation 700 nm). Ladder from top to bottom (kDa): 250, 130, 95, 70, 55, 43, 34, 26. Lanes from left to right: WT, *∆cspC*, *∆cspC/cspC*, *∆cspC/cspC*_Q516A/T540A_, *∆cspC/cspC*_Q516E/T520E_, *∆cspBAC, ∆cspBAC/cspBAC*, *∆cspBAC/cspBA*_R896A_ *cspC*_Q516A/T520A_ (*3xAla*), *∆cspBAC/cspBA*_R896E_ *cspC*_Q516E/T520E_ (*3xGlu*).

Figure S11B & S20A (anti-CspC):


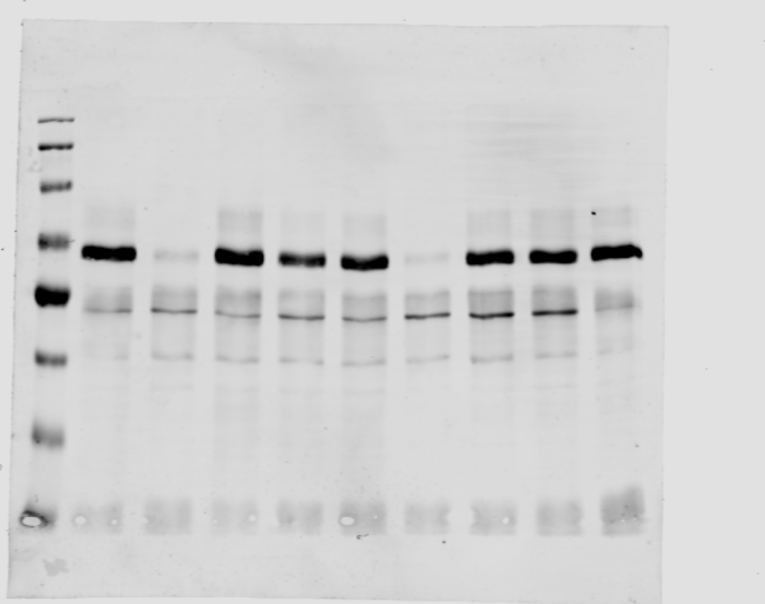


Western blot visualized using an Odyssey LiCOR CLx (2ºAb IRD680, excitation 700 nm). Ladder from top to bottom (kDa): 250, 130, 95, 70, 55, 43, 34, 26. Lanes from left to right: WT, *∆cspBAC*, *∆cspBAC/cspBAC*, *∆cspBAC/cspBA*_D1008A/R1036A_ *cspC*_Q516E/T540E_ (*4x mut*), WT, *∆cspBA*, *∆cspBA/cspBA*, *∆cspBA/cspBA*_Q1090A_. Final lane not applicable to study.

Figure S11B & S20A (anti-CspA):


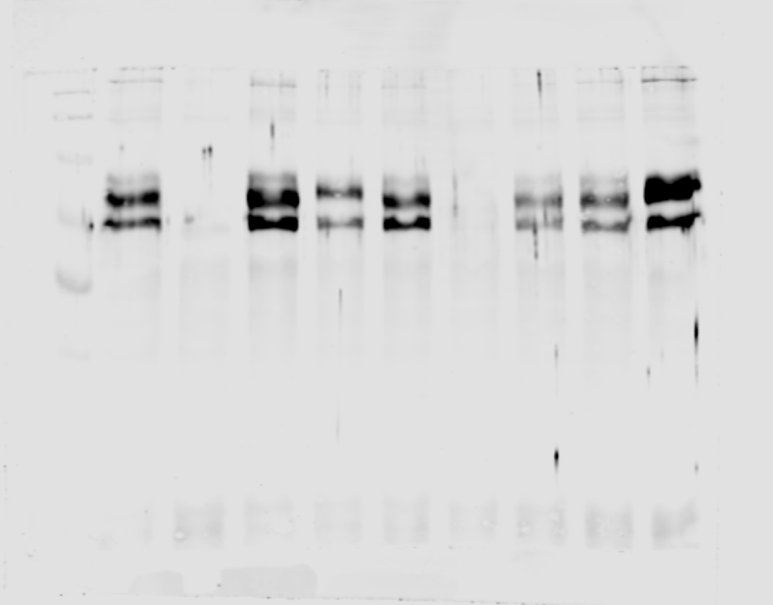


Western blot visualized using an Odyssey LiCOR CLx (2ºAb IRD800, excitation 800 nm). Ladder from top to bottom (kDa): 250, 130, 95, 70, 55. Lanes from left to right: WT, *∆cspBAC*, *∆cspBAC/cspBAC*, *∆cspBAC/cspBA*_D1008A/R1036A_ *cspC*_Q516E/T540E_ (*4x mut*), WT, *∆cspBA*, *∆cspBA/cspBA*, *∆cspBA/cspBA*_Q1090A_. Final lane not applicable to study.

Figure S11B &S20A (anti-CspB):


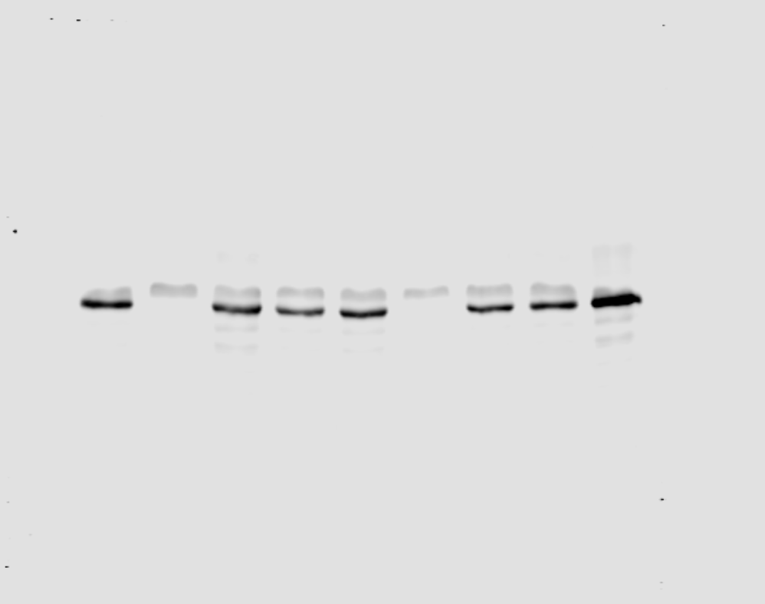


Western blot visualized using an Odyssey LiCOR CLx (2ºAb IRD800, excitation 800 nm). Ladder from top to bottom (kDa): 250, 130, 95, 70, 55. Lanes from left to right: WT, *∆cspBAC*, *∆cspBAC/cspBAC*, *∆cspBAC/cspBA*_D1008A/R1036A_ *cspC*_Q516E/T540E_ (*4x mut*), WT, *∆cspBA*, *∆cspBA/cspBA*, *∆cspBA/cspBA*_Q1090A_. Final lane not applicable to study.

Figure S11B & S20A (anti-SleC):


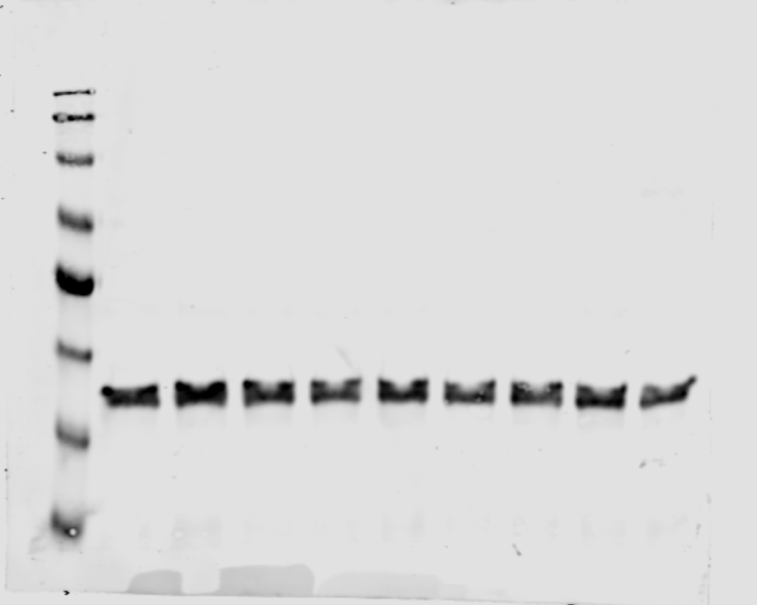


Western blot visualized using an Odyssey LiCOR CLx (2ºAb IRD680, excitation 700 nm). Ladder from top to bottom (kDa): 250, 130, 95, 70, 55, 43, 34, 26. Lanes from left to right: WT, *∆cspBAC*, *∆cspBAC/cspBAC*, *∆cspBAC/cspBA*_D1008A/R1036A_ *cspC*_Q516E/T540E_ (*4x mut*), WT, *∆cspBA*, *∆cspBA/cspBA*, *∆cspBA/cspBA*_Q1090A_. Final lane not applicable to study.

Figure S12A:


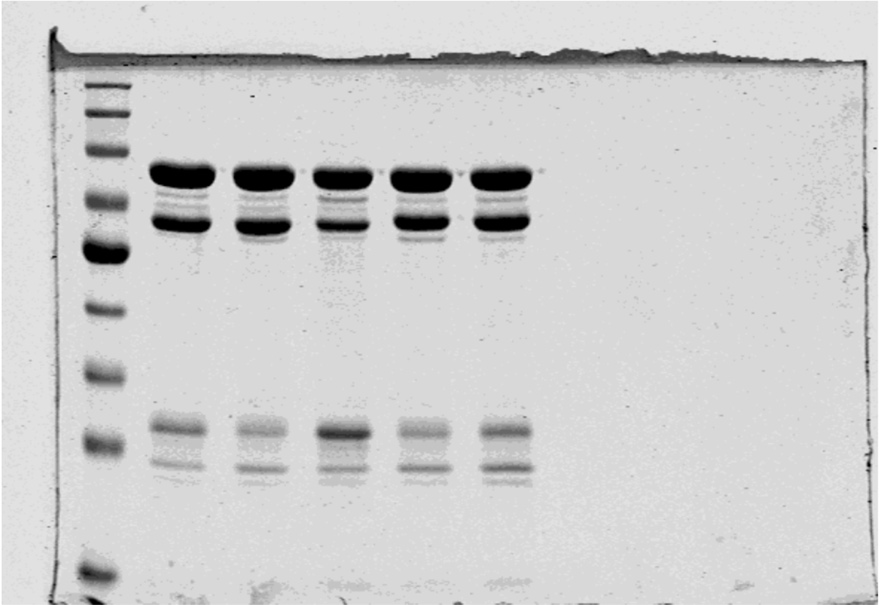


SDS-PAGE of CspC-CPD-His_6_ + CspA co-affinity purification inputs for SEC purification, stained with Coomassie and visualized using an Odyssey LiCOR CLx. Ladder from top to bottom (kDa): 250, 130, 95, 70, 55, 43, 34, 26, 15. Lanes from left to right: WT, CspC_Q516A/T520A_-CPD-His_6_ + CspA, CspC_Q516E/T520E_-CPD-His_6_ + CspA, CspC_Q516A/T520A_-CPD-His_6_ + CspA_R896A_, CspC_Q516E/T520E_-CPD-His_6_ + CspA_R896E_.

Figure S12B & S14E (top):


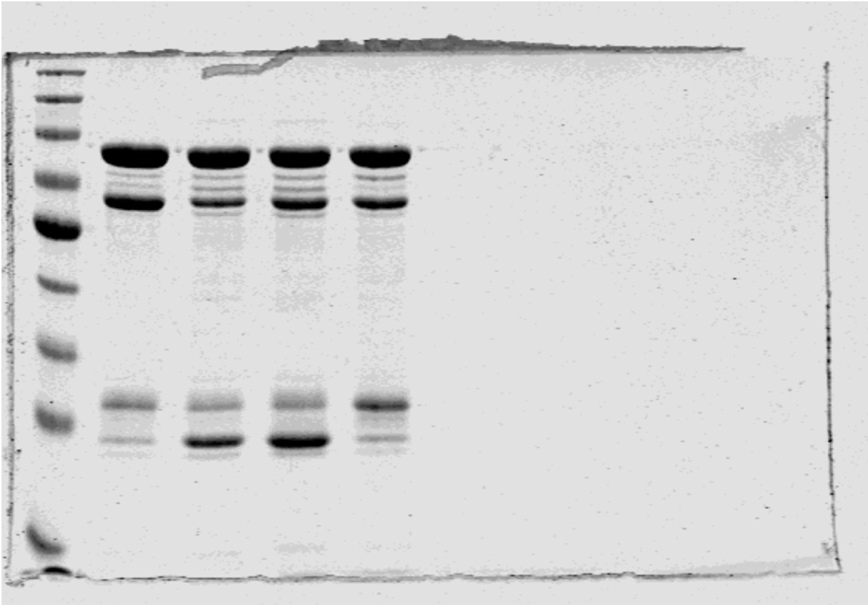


SDS-PAGE of CspC-CPD-His_6_ + CspA co-affinity purification inputs for SEC purification, stained with Coomassie and visualized using an Odyssey LiCOR CLx. Ladder from top to bottom (kDa): 250, 130, 95, 70, 55, 43, 34, 26, 15. Lanes from left to right: WT, CspC_Q516E/T520E_-CPD-His_6_ + CspA_D1008A/R1036A_, CspC-CPD-His_6_ + CspA_D1008A/R1036A_, CspC_Q516E/T520E_-CPD-His_6_ + CspA.

Figure S14E (bottom):


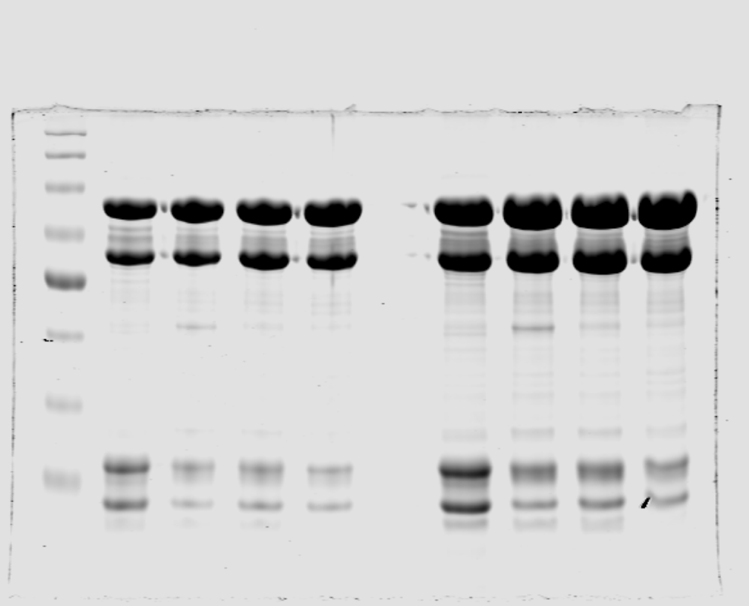


SDS-PAGE of CspC-CPD-His_6_ + CspA co-affinity purification inputs for SEC purification, stained with Coomassie and visualized using an Odyssey LiCOR CLx. Ladder from top to bottom (kDa): 250, 130, 95, 70, 55, 43, 34, 26, 15. Lanes from left to right: WT, CspC_Q516E/T520E_-CPD-His_6_ + CspA_D1008A/R1036A_, CspC-CPD-His_6_ + CspA_D1008A/R1036A_, CspC_Q516E/T520E_-CPD-His_6_ + CspA. Lanes 6 – 10 not applicable to study.

Figure S15 (anti-CspC):


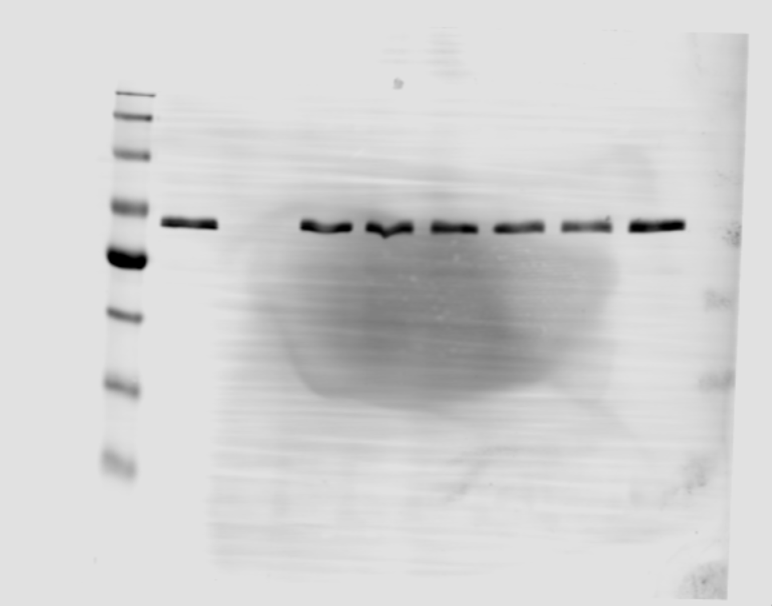


Western blot visualized using an Odyssey LiCOR CLx (2ºAb IRD680, excitation 700 nm). Ladder from top to bottom (kDa): 250, 130, 95, 70, 55, 43, 34, 26. Lanes from left to right: WT, *∆cspBA*, *∆cspBA/cspBA*, *∆cspBA/cspBA*_R896A_, *∆cspBA/cspBA*_R896E_, *∆cspBA/cspBA*_Q1094E/T1098E_, *∆cspBA/cspBA*_R896A/Q1094A/T1098A_ (*3xAla*), *∆cspBA/cspBA*_R896E/Q1094E_.

Figure S15 (anti-CspA):


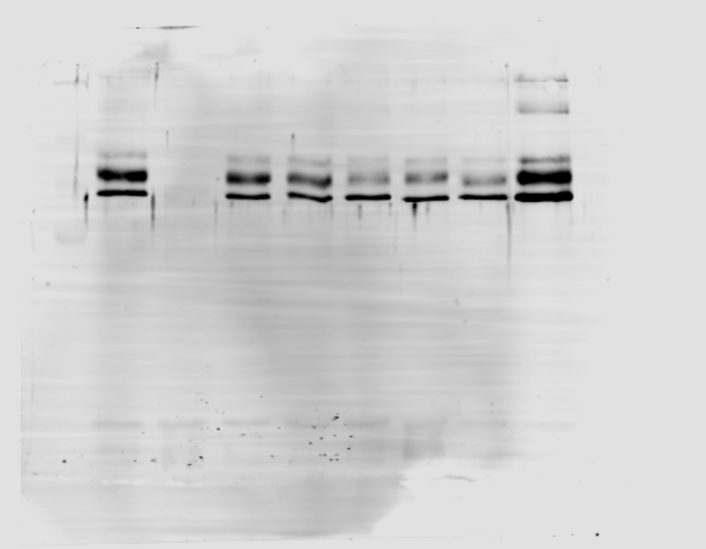


Western blot visualized using an Odyssey LiCOR CLx (2ºAb IRD800, excitation 800 nm). Ladder from top to bottom (kDa): 250, 130, 95, 70, 55, 43, 34, 26. Lanes from left to right: WT, *∆cspBA*, *∆cspBA/cspBA*, *∆cspBA/cspBA*_R896A_, *∆cspBA/cspBA*_R896E_, *∆cspBA/cspBA*_Q1094E/T1098E_, *∆cspBA/cspBA*_R896A/Q1094A/T1098A_ (*3xAla*), *∆cspBA/cspBA*_R896E/Q1094E_.

Figure S15 (anti-CspB):


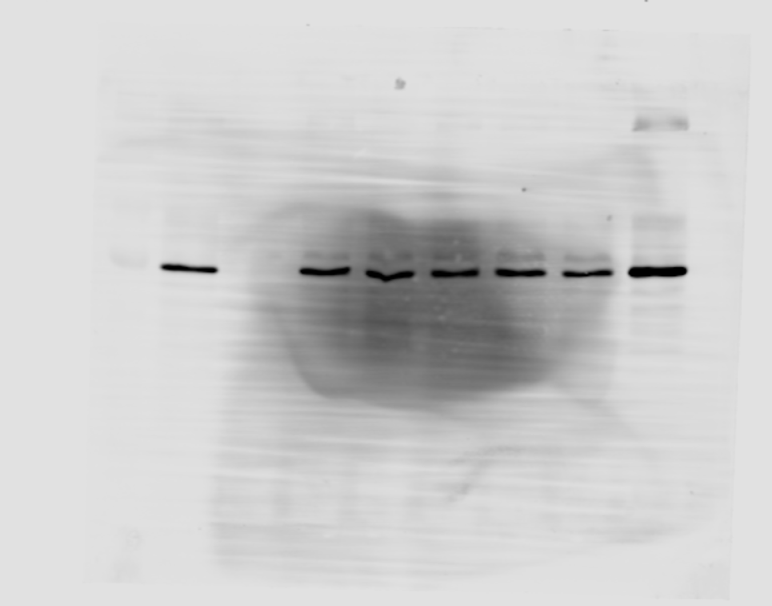


Western blot visualized using an Odyssey LiCOR CLx (2ºAb IRD800, excitation 800 nm). Ladder from top to bottom (kDa): 250, 130, 95, 70, 55, 43, 34, 26. Lanes from left to right: WT, *∆cspBA*, *∆cspBA/cspBA*, *∆cspBA/cspBA*_R896A_, *∆cspBA/cspBA*_R896E_, *∆cspBA/cspBA*_Q1094E/T1098E_, *∆cspBA/cspBA*_R896A/Q1094A/T1098A_ (*3xAla*), *∆cspBA/cspBA*_R896E/Q1094E_.

Figure S15 (anti-SleC):


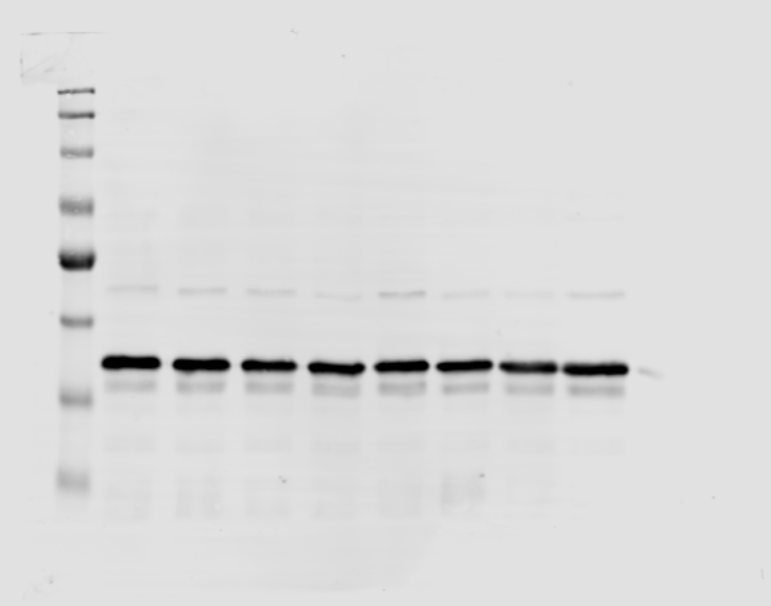


Western blot visualized using an Odyssey LiCOR CLx (2ºAb IRD680, excitation 700 nm). Ladder from top to bottom (kDa): 250, 130, 95, 70, 55, 43, 34, 26. Lanes from left to right: WT, *∆cspBA*, *∆cspBA/cspBA*, *∆cspBA/cspBA*_R896A_, *∆cspBA/cspBA*_R896E_, *∆cspBA/cspBA*_Q1094E/T1098E_, *∆cspBA/cspBA*_R896A/Q1094A/T1098A_ (*3xAla*), *∆cspBA/cspBA*_R896E/Q1094E_.

Figure S17 (top):


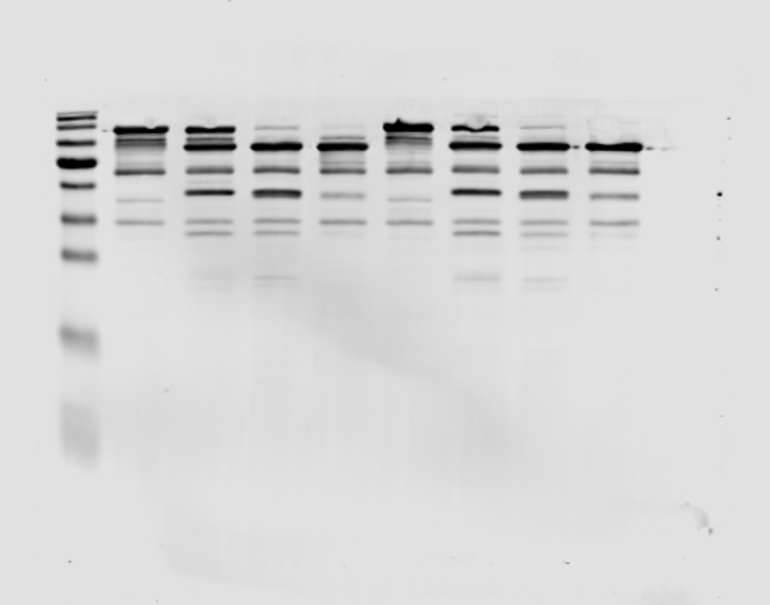


Western blot visualized using an Odyssey LiCOR CLx (2ºAb IRD680, excitation 700 nm). Ladder from top to bottom (kDa): 250, 130, 95, 70, 55, 43, 34, 26, 15, 11. Lanes from left to right: chymotrypsin (µg/mL): 0, 4, 40, 400, 0 + 1 mM TA & 1 mM glycine, 4 + 1 mM TA & 1 mM glycine, 40 + 1 mM TA & 1 mM glycine, 400 + 1 mM TA & 1 mM glycine.

Figure S17 (center):


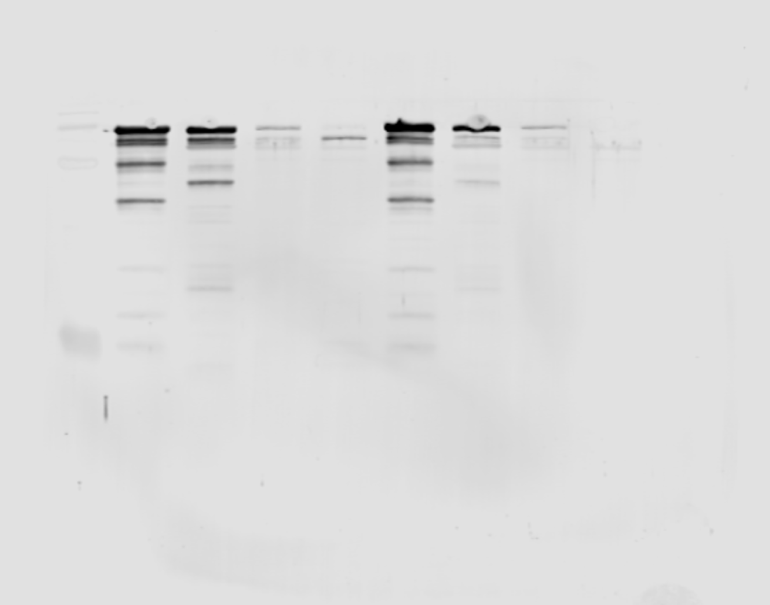


Western blot visualized using an Odyssey LiCOR CLx (2ºAb IRD800, excitation 800 nm). Ladder from top to bottom (kDa): 130, 95, 55, 15. Lanes from left to right: chymotrypsin (µg/mL): 0, 4, 40, 400, 0 + 1 mM TA & 1 mM glycine, 4 + 1 mM TA & 1 mM glycine, 40 + 1 mM TA & 1 mM glycine, 400 + 1 mM TA & 1 mM glycine.

Figure S17 (bottom):


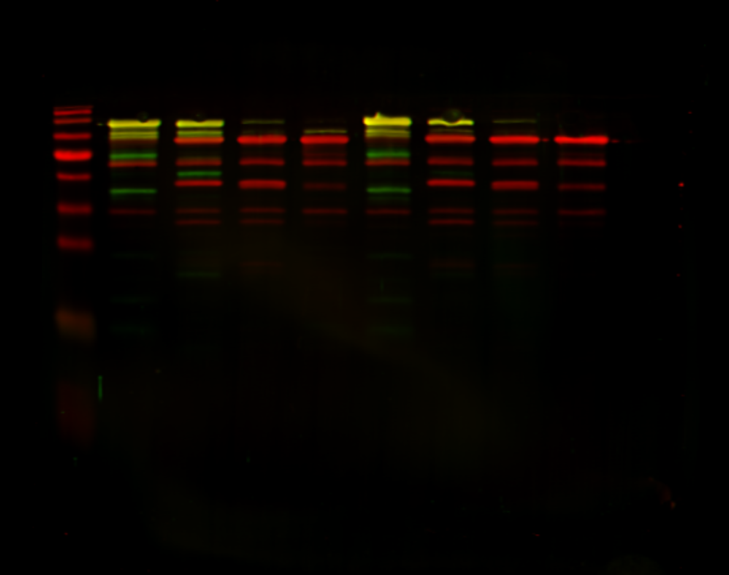


Western blot visualized using an Odyssey LiCOR CLx (2ºAb (red) = IRD680, excitation 700 nm; 2ºAb (green) = IRD800, excitation 800nm). Ladder from top to bottom (kDa): 250, 130, 95, 70, 55, 43, 34, 26, 15. Lanes from left to right: chymotrypsin (µg/mL): 0, 4, 40, 400, 0 + 1 mM TA & 1 mM glycine, 4 + 1 mM TA & 1 mM glycine, 40 + 1 mM TA & 1 mM glycine, 400 + 1 mM TA & 1 mM glycine.

Figure S18A:


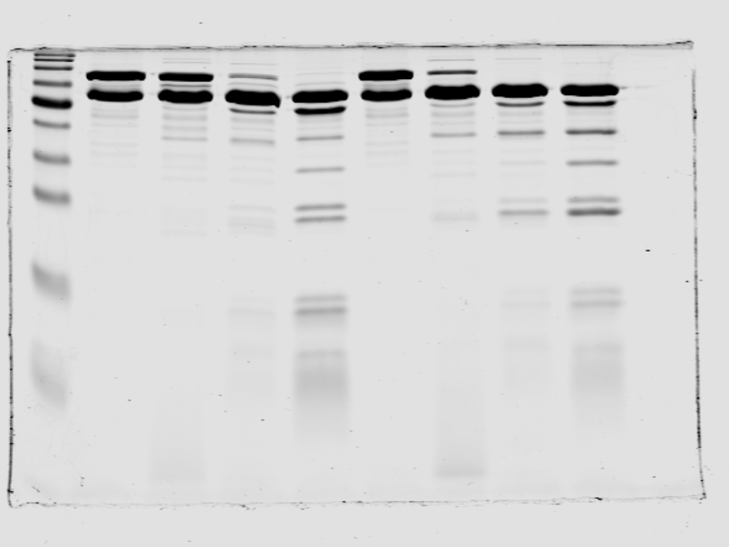


Limited proteolysis resolved using SDS-PAGE, stained with Coomassie, and visualized using an Odyssey LiCOR CLx. Ladder from top to bottom (kDa): 250, 130, 95, 70, 55, 43, 34, 26, 15, 11. Lanes from left to right: chymotrypsin (µg/mL): 0, 4, 40, 400, 0 + 1 mM TA & 1 mM glycine, 4 + 1 mM TA & 1 mM glycine, 40 + 1 mM TA & 1 mM glycine, 400 + 1 mM TA & 1 mM glycine.

Figure S18B:


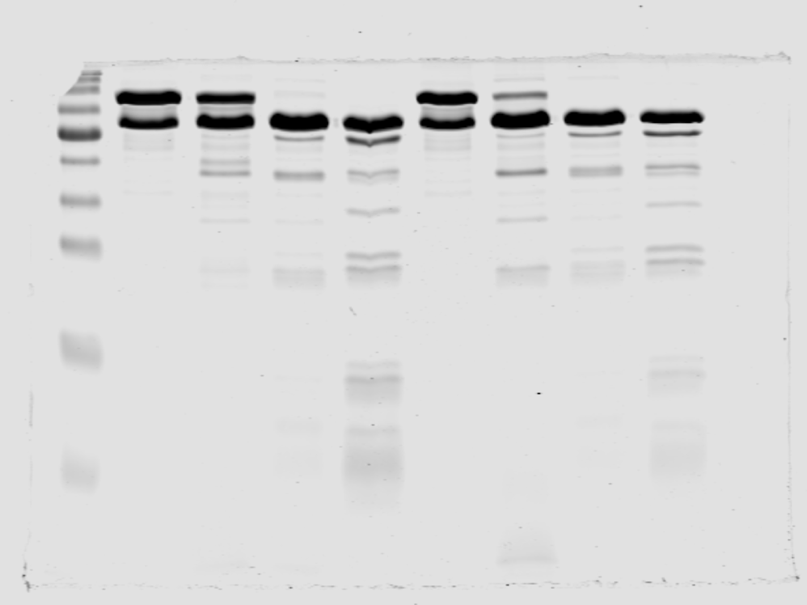


Limited proteolysis resolved using SDS-PAGE, stained with Coomassie, and visualized using an Odyssey LiCOR CLx. Ladder from top to bottom (kDa): 250, 130, 95, 70, 55, 43, 34, 26, 15, 11. Lanes from left to right: chymotrypsin (µg/mL): 0, 4, 40, 400, 0 + 1 mM TA & 1 mM glycine, 4 + 1 mM TA & 1 mM glycine, 40 + 1 mM TA & 1 mM glycine, 400 + 1 mM TA & 1 mM glycine.

Figure S18C:


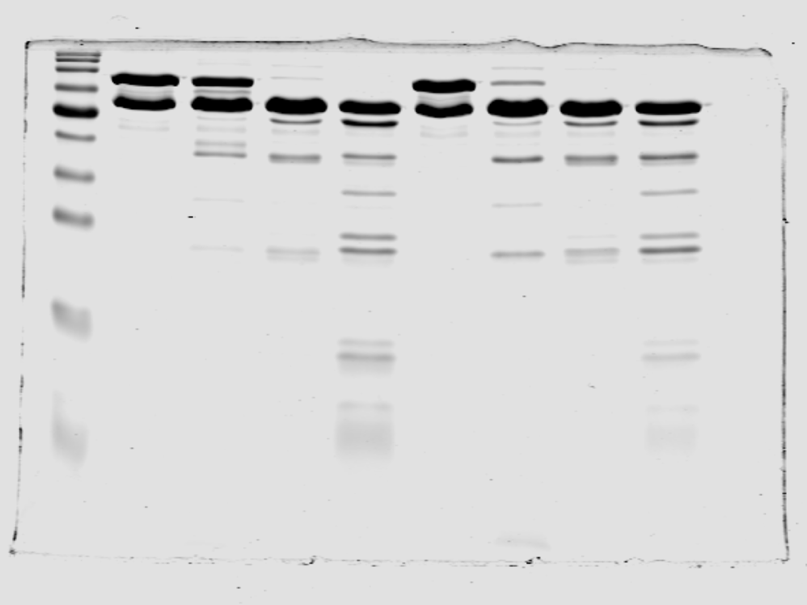


Limited proteolysis resolved using SDS-PAGE, stained with Coomassie, and visualized using an Odyssey LiCOR CLx. Ladder from top to bottom (kDa): 250, 130, 95, 70, 55, 43, 34, 26, 15, 11. Lanes from left to right: chymotrypsin (µg/mL): 0, 4, 40, 400, 0 + 1 mM TA & 1 mM glycine, 4 + 1 mM TA & 1 mM glycine, 40 + 1 mM TA & 1 mM glycine, 400 + 1 mM TA & 1 mM glycine.
